# Structural Characterization of Lithium and Sodium Bulky Bis(silyl)amide Complexes

**DOI:** 10.3390/molecules23051138

**Published:** 2018-05-10

**Authors:** Hannah M. Nicholas, Conrad A. P. Goodwin, Jon G. C. Kragskow, Selena J. Lockyer, David P. Mills

**Affiliations:** School of Chemistry, The University of Manchester, Oxford Road, Manchester M13 9PL, UK; hannah.nicholas@manchester.ac.uk (H.M.N.); conrad.goodwin@manchester.ac.uk (C.A.P.G.); jon.kragskow@student.manchester.ac.uk (J.G.C.K.); selena.lockyer@postgrad.manchester.ac.uk (S.J.L.)

**Keywords:** bis(silyl)amide, ligand, lithium, sodium

## Abstract

Alkali metal amides are vital reagents in synthetic chemistry and the bis(silyl)amide {N(SiMe_3_)_2_} (N′′) is one of the most widely-utilized examples. Given that N′′ has provided landmark complexes, we have investigated synthetic routes to lithium and sodium bis(silyl)amides with increased steric bulk to analyse the effects of R-group substitution on structural features. To perform this study, the bulky bis(silyl)amines {HN(Si^t^BuMe_2_)(SiMe_3_)}, {HN(Si^i^Pr_3_)(SiMe_3_)}, {HN(Si^t^BuMe_2_)_2_}, {HN(Si^i^Pr_3_)(Si^t^BuMe_2_)} and {HN(Si^i^Pr_3_)_2_} (**1**) were prepared by literature procedures as colourless oils; on one occasion crystals of **1** were obtained. These were treated separately with ^n^BuLi to afford the respective lithium bis(silyl)amides [Li{μ-N(Si^t^BuMe_2_)(SiMe_3_)}]_2_ (**2**), [Li{μ-N(Si^i^Pr_3_)(SiMe_3_)}]_2_ (**3**), [Li{N(Si^t^BuMe_2_)_2_}{μ-N(Si^t^BuMe_2_)_2_}Li(THF)] (**4**), [Li{N(Si^i^Pr_3_)(Si^t^BuMe_2_)}(DME)] (**6**) and [Li{N(Si^i^Pr_3_)_2_}(THF)] (**7**) following workup and recrystallization. On one occasion during the synthesis of **4** several crystals of the ‘ate’ complex [Li_2_{μ-N(Si^t^BuMe_2_)_2_}(μ-^n^Bu)]_2_ (**5**) formed and a trace amount of [Li{N(Si^i^Pr_3_)_2_}(THF)_2_] (**8**) was identified during the recrystallization of **7**. The reaction of {HN(Si^t^BuMe_2_)_2_} with NaH in the presence of 2 mol % of NaO^t^Bu gave crystals of [Na{μ-N(Si^t^BuMe_2_)_2_}(THF)]_2_ (**9-THF**), whilst [Na{N(Si^i^Pr_3_)_2_}(C_7_H_8_)] (**10**) was prepared by deprotonation of **1** with ^n^BuNa. The solid-state structures of **1**–**10** were determined by single crystal X-ray crystallography, whilst **2**–**4**, **7**, **9** and **10** were additionally characterized by NMR and FTIR spectroscopy and elemental microanalysis.

## 1. Introduction

Alkali metal amides, {MNR_2_} (M = Li-Cs), are ubiquitous reagents in synthetic chemistry and their steric and electronic properties are readily tuned by R-group substitution [1,2,3]. Their popularity with synthetic chemists can be attributed to a combination of their strong basicity, low nucleophilicity, hydrocarbon solvent solubility and facile preparation from commercially available starting materials [4]. Lithium amides are more frequently utilized than the heavier group 1 congeners as they can be synthesized directly from the parent amine and ^n^BuLi and used in situ; the heavy alkali metal amides tend to be less soluble in hydrocarbons and additionally suffer from decomposition pathways in non-coordinating solvents [1,2,3,4].

The bulky bis(silyl)amide {N(SiMe_3_)_2_} (N′′) is relatively soft due to negative hyperconjugation provided by Si atoms and its alkali metal salts have been used extensively as ligand transfer reagents in the preparation of landmark low-coordinate s-, p-, d- and f-block complexes by salt metathesis reactions with metal/metalloid halide precursors [4,5,6,7]. Although these reagents are often used in situ, the solvent-free solid-state structures of the lighter group 1 salts LiN′′ [8,9], NaN′′ [10] and KN′′ [11] have been reported previously. Interestingly, although synthetic routes to alkali metal bis(silyl)amides with larger R groups, {MN(SiR_3_)_2_} (R = Me, ^t^Bu, ^i^Pr, etc.), were reported 40 years ago [12], there was only one structurally characterized wholly aliphatic group 1 bis(silyl)amide, [K{μ-N(Si^t^BuMe_2_)(SiMe_3_)}]_2_ [13], reported using these methodologies before 2014.

**Figure 1:**
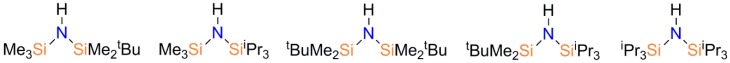
The bulky bis(silyl)amines utilized herein (l-r): {HN(SiMe_2_^t^Bu)(SiMe_3_)}, {HN(Si^i^Pr_3_)(SiMe_3_)}, {HN(SiMe_2_^t^Bu)_2_}, {HN(SiPr^i^_3_)(SiMe_2_^t^Bu)} and {HN(SiPr^i^_3_)_2_} (**1**).

Recently, our group disclosed synthetic routes to a series of bulky bis(silyl)amines based upon this previous work (Figure 1) [14]. We have utilized the potassium salts of these ligands to synthesize a catalogue of low-coordinate s- and f-block complexes by salt metathesis methodologies [15,16,17,18,19,20,21,22]. In these previous studies, we found that the potassium bis(silyl)amides generally react with metal iodide precursors to give target complexes in moderate crystalline yields but for metal chloride precursors poor yields have resulted. The elimination of potassium iodide is advantageous as it is insoluble in most organic solvents. We envisaged that lithium and sodium ligand transfer agents may provide improved yields of products with metal chloride precursors, as had been seen previously in N′′ chemistry [4,5,6,7]. Whilst these lithium and sodium bulky bis(silyl)amides can be used in situ in future, we report their synthesis, isolation and structural characterization herein.

## 2. Results

### 2.1. Synthesis

**Scheme 1:**
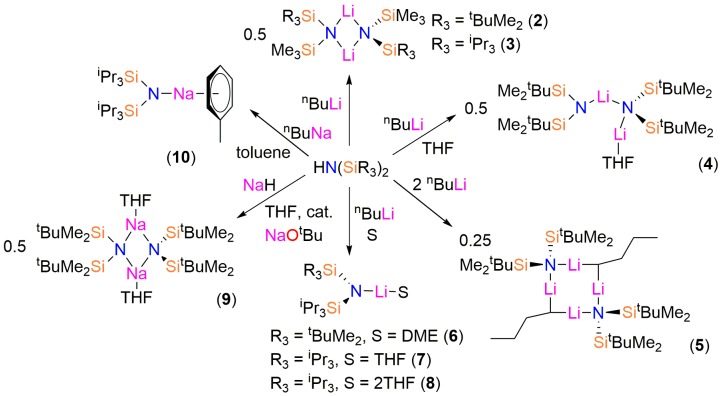
Synthesis of the lithium and sodium bis(silyl)amide complexes **2**–**10**. The structures presented are those determined by single crystal X-ray crystallography; variable desolvation for **4**, **9** and **10** occurs when samples are exposed to dynamic vacuum.

The bis(silyl)amines {HN(SiMe_2_^t^Bu)(SiMe_3_)}, {HN(Si^i^Pr_3_)(SiMe_3_)}, {HN(SiMe_2_^t^Bu)_2_}, {HN(SiPr^i^_3_)(SiMe_2_^t^Bu)} and {HN(SiPr^i^_3_)_2_} (**1**) were prepared as colourless oils following published procedures using liquid ammonia, ^n^BuLi and the parent trialkylsilylchlorides [12,13,14]. During the synthesis of **1** crystals suitable for single crystal X-ray diffraction were grown and the solid-state structure was determined (see below). The lithium bis(silyl)amides [Li{μ-N(Si^t^BuMe_2_)(SiMe_3_)}]_2_ (**2**), [Li{μ-N(Si^i^Pr_3_)(SiMe_3_)}]_2_ (**3**), [Li{N(Si^t^BuMe_2_)_2_}{μ-N(Si^t^BuMe_2_)_2_}Li(THF)] (**4**), [Li{N(Si^i^Pr_3_)(Si^t^BuMe_2_)}(DME)] (**6**) and [Li{N(Si^i^Pr_3_)_2_}(THF)] (**7**) were isolated by recrystallization from various solvents following straightforward synthesis from the parent amine and ^n^BuLi, using analogous procedures to the synthesis of LiN′′ [8,9] (Scheme 1). On one occasion during the synthesis of **4** several crystals of the ‘ate’ complex [Li_2_{μ-N(Si^t^BuMe_2_)_2_}(μ-^n^Bu)]_2_ (**5**) formed and a trace amount of [Li{N(Si^i^Pr_3_)_2_}(THF)_2_] (**8**) was identified during the recrystallization of **7**.

As symmetrical bis(silyl)amides have found most synthetic utility to date [4,5,6,7] we also targeted sodium salts of {HN(SiMe_2_^t^Bu)_2_} and **1**. We found that the reaction of {HN(Si^t^BuMe_2_)_2_} with NaH in toluene was sluggish, so adapting a procedure reported by Arnold for the improved synthesis of NaN′′ [23] we added 2 mol % of NaO^t^Bu and refluxed the reaction mixture for 16 h. Crystals of [Na{μ-N(Si^t^BuMe_2_)_2_}(THF)]_2_ (**9-THF**) were obtained following treatment with THF and recrystallization from pentane. Similarly, the reaction of **1** with NaH was also slow; on this occasion, the sodium bis(silyl)amide [Na{N(Si^i^Pr_3_)_2_}(C_7_H_8_)] (**10**) was synthesized by deprotonation of **1** with ^n^BuNa [24] in hexane, followed by recrystallization from toluene.

As **5**, **6** and **8** were only isolated in trace amounts no supporting data could be obtained to support the solid-state structures (see below). However, for **2**–**4**, **7**, **9** and **10** the crystalline yields were sufficient to carry out analysis of bulk sample purity by multinuclear NMR spectroscopy and elemental analysis. Low carbon values were consistently obtained in microanalysis experiments of **4** and **7**. As <5% protic impurities were seen in the ^1^H-NMR spectra of these samples, we attribute this observation to the formation of silicon carbides; we have commented previously that this can lead to incomplete combustion of complexes of these bis(silyl)amides [17,18,19,20,21,22].

### 2.2. Spectroscopy

The ^1^H and ^13^C{^1^H}-NMR spectra for **2**–**4**, **7**, **9** and **10** are similar to those previously obtained for the respective potassium congeners [13,14] but vary by the solvents that are coordinated to the alkali metals due to crystallization conditions. For example, THF is seen in the solid-state structures of **4**, **7**, **8** and **9-THF**, whilst toluene is seen in the structure of **10** (see below). Interestingly, when these crystalline samples were dried in vacuo they desolvated to various degrees based on integrals in the ^1^H-NMR spectra. In the case of **7** no desolvation was observed, for **4** and **10** approximately half of the solvent was removed and for **9-THF** complete desolvation occurred when the sample was heated in vacuo to give **9**. Variable solvent coordination was previously seen in the ^1^H and ^13^C{^1^H}-NMR spectra of [K{μ-N(Si^t^BuMe_2_)(SiMe_3_)}]_2_ [13] and [K{μ-N(Si^t^BuMe_2_)(SiMe_3_)}(C_7_H_8_)]_2_ [14] and the desolvation of strongly bound solvent molecules under vacuum is commonly seen in alkali metal bis(silyl)amide chemistry [4,5,6]. Only one signal is observed for each methine and methyl group in the ^1^H-NMR spectra of **2**–**4**, **7**, **9** and **10** in C_6_D_6_ at 298 K, indicating that any asymmetry observed in the solid state (see below) is not maintained in solution. Variable degrees of aggregation in non-coordinating solvents is a general feature of s-block N′′ chemistry [4,5,6]; approximate sizes of such aggregates have routinely been studied by ^1^H-DOSY-NMR spectroscopy but an investigation of the solution dynamics of the complexes herein are beyond the scope of this study, which focuses on solid state structural characterization.

The ^29^Si{^1^H}-NMR spectra (δ_Si_: −13.66 and −5.89 (**2**); −12.03 and −1.38 (**3**); −6.15 (**4**); −10.39 (**7**); −11.67 (**9**); −13.82 (**10**)) are diagnostic of the number of silicon environments and these signals are all upfield of their respective potassium congeners (δ_Si_: −20.60 and −12.31, [K{μ-N(Si^t^BuMe_2_)(SiMe_3_)}(C_7_H_8_)]_2_; −22.80 and −9.57, [K{μ-N(Si^i^Pr_3_)(SiMe_3_)}]_∞_; −15.72, [{K[μ-N(Si^t^BuMe_2_)_2_]}_2_(C_7_H_8_)]_∞_; −17.69 and −13.82, [K{N(SiPr^i^_3_)(Si^t^BuMe_2_)}]_∞_; −16.31, [K{N(SiPr^i^_3_)_2_}]_∞_) [14]. The significant difference in ^29^Si chemical shifts shows that the alkali metals remain coordinated by amides in solution. ^7^Li{^1^H}-NMR spectroscopy was also performed for the lithium salts and broad signals at similar chemical shifts were observed (δ_Li_: 1.16 (**2**); 1.47 (**3**); 1.05 (**4**); 1.00 (**7**)); this also correlates with dynamic aggregation processes occurring in solution. Finally, FTIR spectra were obtained for **2**–**4**, **7**, **9** and **10**; as expected these did not exhibit any remarkable features so we do not comment on these further.

### 2.3. Structural Characterization

**Figure 2:**
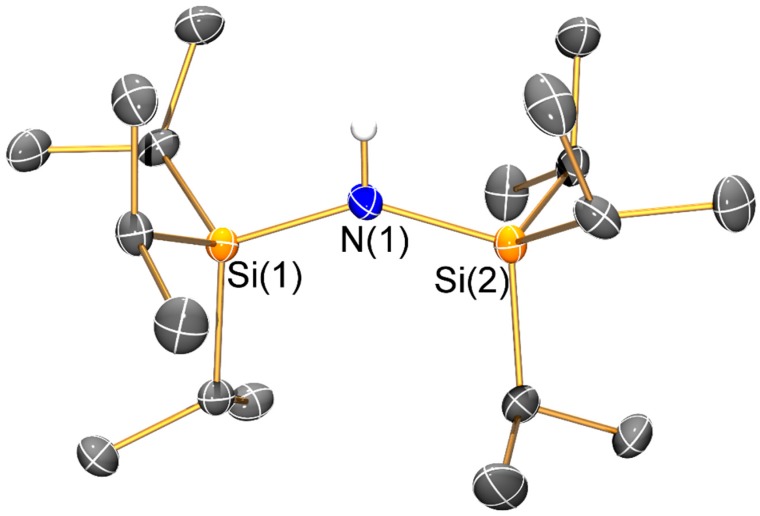
Molecular structure of {HN(Si^i^Pr_3_)_2_} (**1**) with selective atom labelling. Displacement ellipsoids set at 30% probability level and hydrogen atoms apart from H1 omitted for clarity.

**Figure 3:**
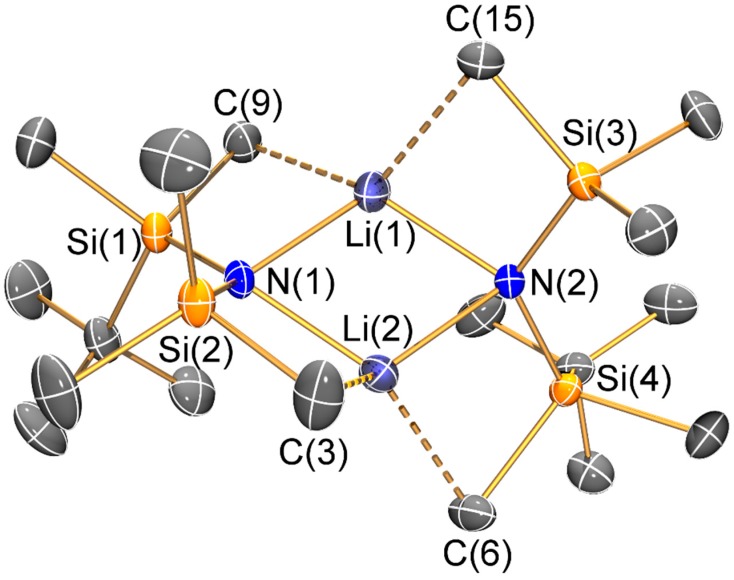
Molecular structure of [Li{μ-N(Si^t^BuMe_2_)(SiMe_3_)}]_2_ (**2**) with selective atom labelling. Displacement ellipsoids set at 30% probability level and hydrogen atoms omitted for clarity.

**Figure 4:**
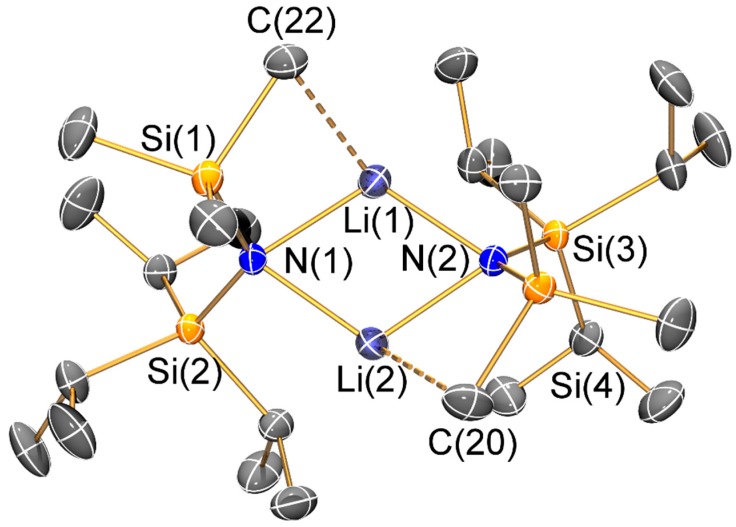
Molecular structure of [Li{μ-N(Si^i^Pr_3_)(SiMe_3_)}]_2_ (**3**) with selective atom labelling. Displacement ellipsoids set at 30% probability level and hydrogen atoms omitted for clarity.

**Figure 5:**
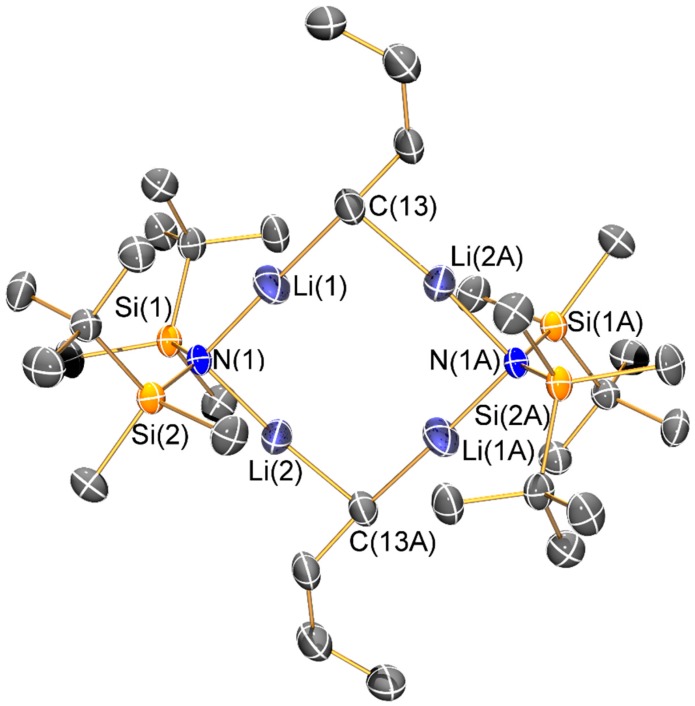
Molecular structure of [Li_2_{μ-N(Si^t^BuMe_2_)_2_}(μ-^n^Bu)]_2_ (**5**) with selective atom labelling. Displacement ellipsoids set at 30% probability level and hydrogen atoms omitted for clarity. Symmetry operation to generate equivalent atoms: *x*, *y*, *z*.

**Figure 6:**
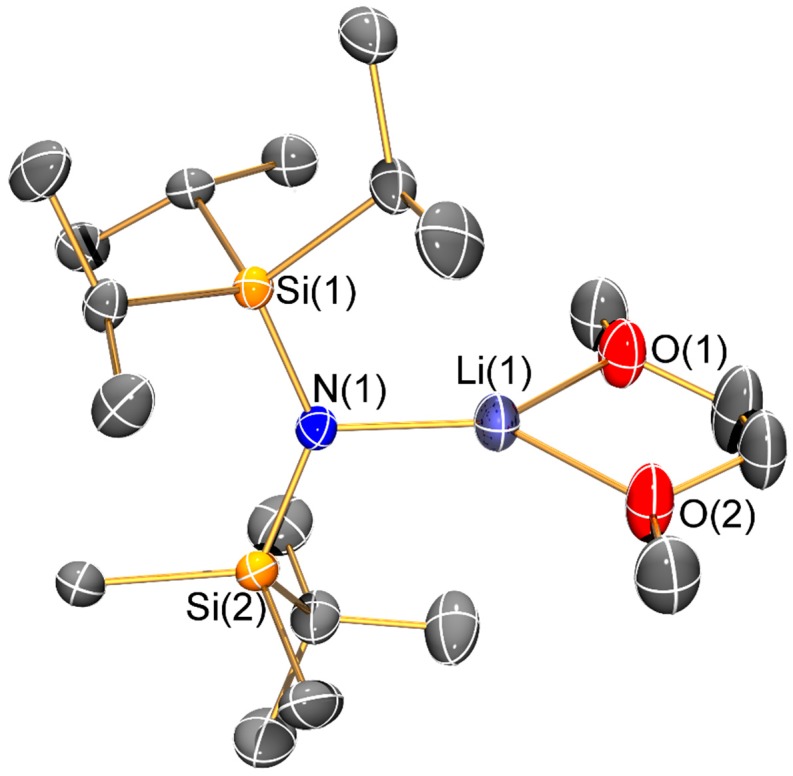
Molecular structure of [Li{N(Si^i^Pr_3_)(Si^t^BuMe_2_)}(DME)] (**6**) with selective atom labelling. Displacement ellipsoids set at 30% probability level and hydrogen atoms omitted for clarity.

**Figure 7:**
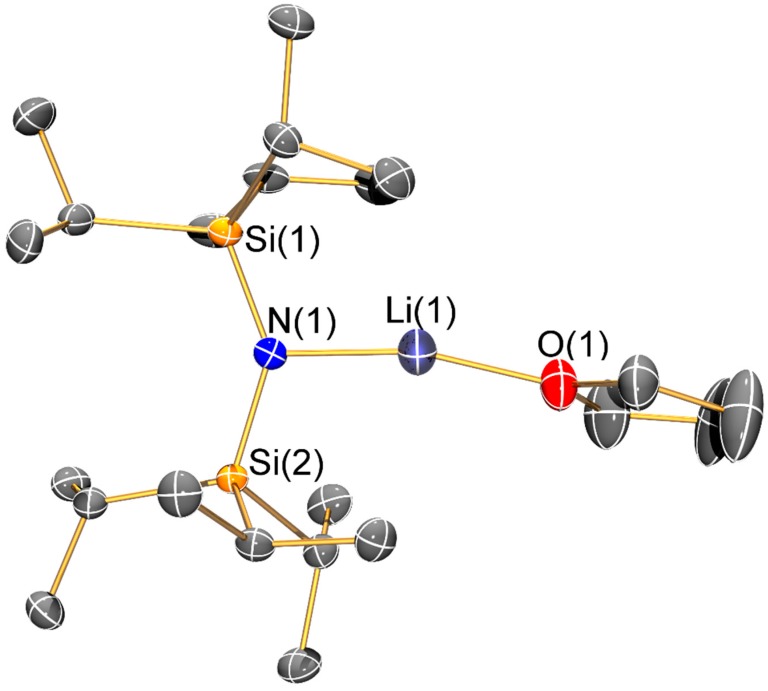
Molecular structure of [Li{N(Si^i^Pr_3_)_2_}(THF)] (**7**) with selective atom labelling. Displacement ellipsoids set at 30% probability level and hydrogen atoms omitted for clarity.

**Figure 8:**
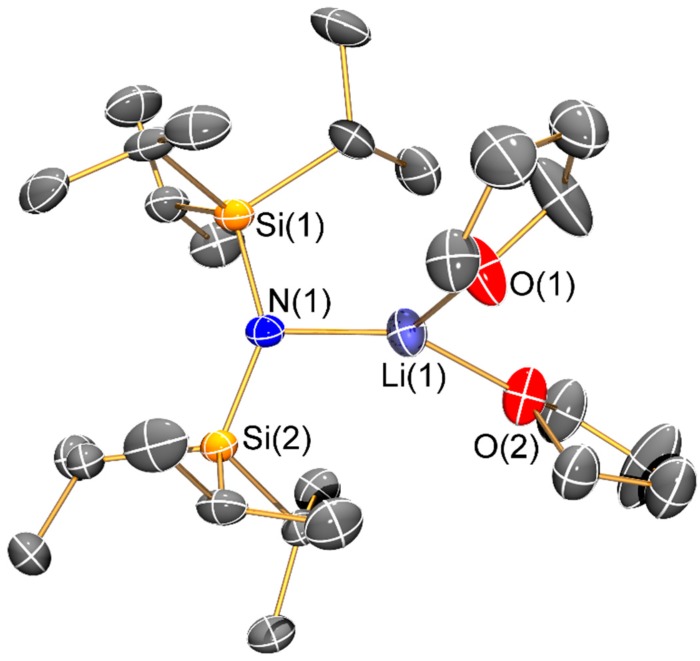
Molecular structure of [Li{N(Si^i^Pr_3_)_2_}(THF)_2_] (**8**) with selective atom labelling. Displacement ellipsoids set at 30% probability level and hydrogen atoms omitted for clarity.

**Figure 9:**
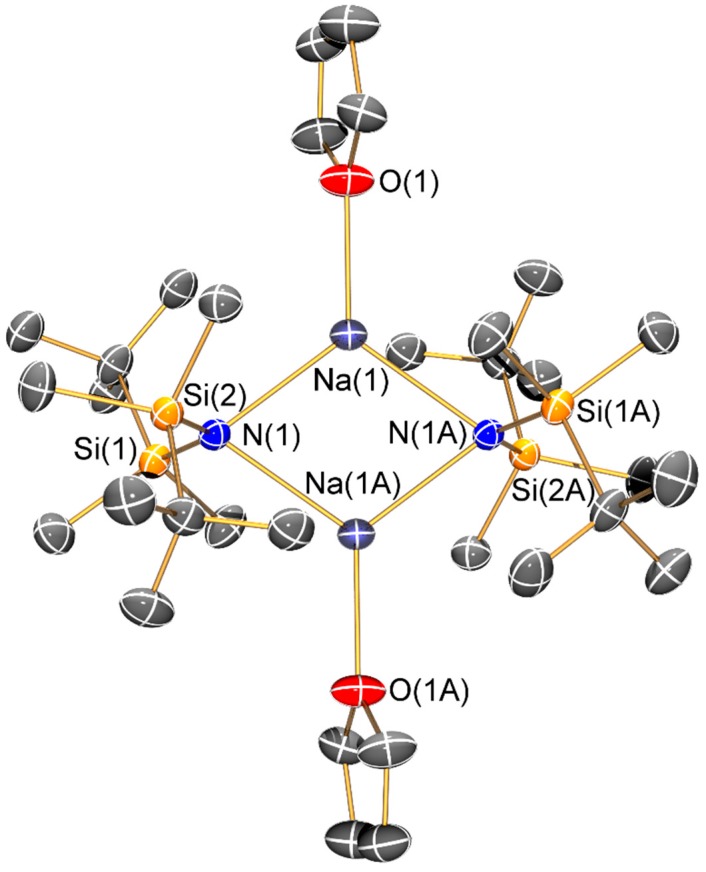
Molecular structure of [Na{μ-N(Si^t^BuMe_2_)_2_}(THF)]_2_ (**9-THF**) with selective atom labelling. Displacement ellipsoids set at 30% probability level and hydrogen atoms omitted for clarity. Symmetry operation to generate equivalent atoms: *x*, *y*, *z*.

**Figure 10:**
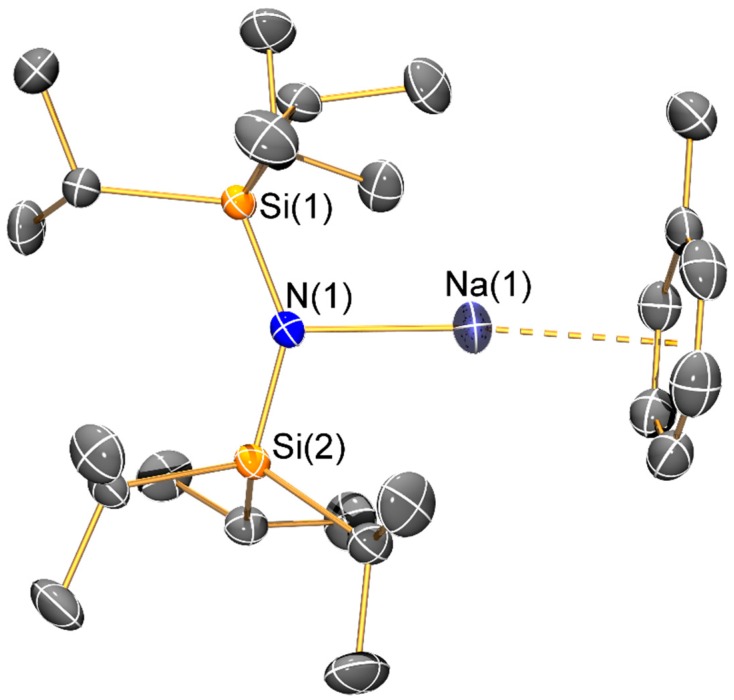
Molecular structure of [Na{N(Si^i^Pr_3_)_2_}(C_7_H_8_)] (**10**) with selective atom labelling. Displacement ellipsoids set at 30% probability level and hydrogen atoms omitted for clarity.

The solid-state structures of **1**–**10** were determined by single crystal XRD (**1**–**3** and **5**–**10** are depicted in Figure 2, Figure 3, Figure 4, Figure 5, Figure 6, Figure 7, Figure 8, Figure 9 and Figure 10 and selected bond lengths and angles are compiled in Table 1; see Supplementary Materials Tables S1–S2 for selected crystallographic data and Figure S29 for the structure of **4**). The dataset for **4** has low completeness, which resulted from the incorrect symmetry being identified in the pre-experiment, so the data collection strategy was insufficient. Unfortunately, this was not identified until after the crystal was removed from the cryostream and the sensitivity of the crystals to desolvation precluded recollection. Therefore, we only include the single crystal XRD data for **4** in the Supplementary Materials to show the identity of the complex and do not comment on this incomplete dataset further.

The mean N–Si distance [1.736(2) Å] of **1** is similar to that previously seen for the electron diffraction structure of HN′′ (1.735(12) Å) [25] and the single crystal X-ray structure of the aromatic bulky bis(silyl)amine HN(SiPh_3_)_2_ (mean N–Si: 1.722(4) Å) [26]; however, the Si–N–Si angle of **1** (145.43(8)°) is less bent than in both HN′′ (125.5(2)°) [25] and HN(SiPh_3_)_2_ (136.1(2)°) [26]. As with the unsolvated dimeric Li salts **2** and **3**, the variation of Si–N–Si angles (**2**: 125.3(2)°; **3**: 127.9(2)°) can be attributed to steric effects. Both **2** and **3** exhibit bridging bis(silyl)amide ligands and planar Li_2_N_2_ cores, which contrasts with the trimeric Li_3_N_3_ structure of [Li(μ-N′′)]_3_ [8]; though it is noteworthy that dimeric [Li(μ-N′′)]_2_ is well-known to dominate in aliphatic solutions [27]. Despite this structural difference, the Li–N bond lengths (**2**: 2.002(10) Å mean; **3**: 2.030(8) Å mean) are similar to those previously reported for [Li(μ-N′′)]_3_ (range 1.983(12)–2.022(13) Å) [8]. As a result of the dimeric structures adopted by **2** and **3**, a number of close Li···C electrostatic contacts are observed: for **2** there are four Li···C distances < 2.5 Å (range 2.378(6)–2.427(6) Å), whilst for **3** there are only two close Li···C contacts (2.378(6) and 2.392(7) Å); again, these differences can be attributed to variable ligand steric effects.

The structure of the ‘ate’ complex **5** can be viewed as a distorted Li_4_ square motif, with the edges alternately bridged by two bis(silyl)amides and two *n*-butyl anions. The ability of N′′ to bridge metal centres is well-documented [4,5,6]; we have previously only seen this mode for the bulkier {N(Si^t^BuMe_2_)_2_} ligand on a limited number of occasions to date [14,19]. Structurally characterized complexes containing unsupported bridging *n*-butyl anions are rare [28,29,30] and to the best of our knowledge this is the first example where two lithium amides are bridged by *n*-butyl anions. The Li–C distances in **5** (2.037(9) Å) are comparable to these previous examples, for example, [(L)Li(µ-^n^Bu)Mg(L)] (L = {2-Me_3_SiNH-6-MeC_5_H_3_N}, Li–C: 2.04(5) Å) [28], whilst the mean Li–N distance (1.97(2) Å) is similar to that seen in **2** and **3**.

The solvated monomeric lithium bis(silyl)amides **6**–**8** will be discussed together. The three-coordinate lithium centre of **6** is chelated by a molecule of DME; this is noteworthy as monomeric [Li(N′′)(DME)] was predicted to be the most stable structure in DME solutions of LiN′′ but this was not observed in solution or the solid state and dimeric [Li(μ-N′′)(κ^1^-DME)]_2_ was identified by single crystal XRD [31]. We attribute the adoption of a monomeric structure in **6** to steric effects; this leads to a relatively short Li–N bond (1.894(13) Å) compared to the mean Li–N distances in [Li(μ-N′′)(κ^1^-DME)]_2_ (2.032(8) Å) [31]. For the most sterically demanding bis(silyl)amide, one molecule of THF is seen in two-coordinate **7**, whilst three-coordinate **8** contains two THF donors. Whilst these monomeric structures differ from that of dimeric [Li(μ-N′′)(THF)]_2_ [32,33], they are comparable to the structure of the *tris*-THF solvated potassium congener, [K{N(SiPr^i^_3_)_2_}(THF)_3_] [14]. The Li–N distance is shorter in **7** (1.893(4) Å) than in **8** (1.953(6) Å) and the same trend is seen for the Li–O distances (**7**: 1.866(3) Å; **8**: 1.972(7) Å mean); these differences are due to the variable degree of solvation in the two complexes.

The sodium bis(silyl)amide **9-THF** is dimeric in the solid state, with each three-coordinate sodium centre coordinated by two bridging amides and a pendant THF molecule to give a planar Na_2_N_2_ core. The same motif was previously seen in [Na(μ-N′′)(THF)]_2_ [34], indicating that whilst the increased steric bulk in **9-THF** has effected an increase in the respective Na–O and Na–N distances (2.267(2) and 2.399(2) Å in [Na(μ-N′′)(THF)]_2_ versus 2.328(3) Å 2.490(2) Å in **9-THF**), this is not sufficient to induce a major structural change. In contrast, **10** was synthesized by an alternative synthetic route and was not exposed to coordinating solvents, so it crystallized as a discrete monomer with the sodium centre coordinated by a terminal bis(silyl)amide ligand and a molecule of toluene. As π-arene interactions are more common for the softer, heavier alkali metals, the structure of **10** (Na···Ph_centroid_: 2.527(2) Å) can be viewed as a combination of the arene-free potassium congener [K{N(SiPr^i^_3_)_2_}]_∞_ and dimeric toluene-bound [{K[μ-N(Si^t^BuMe_2_)_2_]}_2_(C_7_H_8_)]_∞_ (K···Ph_centroid_: 3.385(2) Å) [14], though like solvent-free NaN′′ [10] these literature examples are aggregated in the solid state, which is not the case for **10**. The Na–N bond length of **10** (2.261(2) Å) is shorter than that seen for dimeric **9-THF**, which we attribute to the terminal bis(silyl)amide binding mode adopted in **10**.

## 3. Discussion

The focus of this study has been a structural investigation of lithium and sodium salts; thus, we have adjusted solvent systems only in order to grow crystals suitable for characterization by XRD. We were not able to use the same solvent system for all complexes, which precluded some internal comparisons of the adopted structures but as a considerable number of solvated alkali metal N′′ complexes are known [4,5,6] these could all be rationalized. Indeed, it is evident that structural differences with changes in steric bulk for the complexes herein correlate with those seen previously for potassium salts of the same ligands [14]. A comparison of the solid-state structures of lithium N′′ complexes with the bulkier bis(silyl)amide lithium complexes herein shows that increased silyl group size gives decreased aggregation, tending towards monomeric structures for the largest silyl groups. A detailed analysis of the aggregation of these complexes in various solvents is beyond the scope of this report but could provide interesting comparisons to the better-understood solution behaviour of alkali metal N′′ complexes [4,5,6]. Indeed, the isolation of **5** indicates that structurally complex aggregates can form.

Although the isolated crystalline yields of lithium and sodium bis(silylamides) herein were uniformly low, analysis of the ^1^H-NMR spectra of reaction mixtures indicated that the products **2**–**4**, **6**, **7**, **9-THF** and **10** were synthesized in nearly quantitative yields, so these can be used in situ in future reactivity studies. The synthesis of lithium salts is facile, with ^n^BuLi effecting deprotonation at room temperature; sodium salt preparation is less straightforward as either refluxing conditions or the use of a non-standard reagent such as ^n^BuNa is required. We envisage that these lighter congeners will find synthetic utility as alternative ligand transfer agents to the potassium bis(silyl)amides that we have used extensively in the preparation of f-block complexes [14,15,16,17,18,19,20,21]. Whilst the potassium salts have proved effective in salt metathesis reactions with metal iodide precursors due to facile potassium iodide elimination, we envisage that the lithium and sodium reagents herein could give improved yields when metal chlorides are used as starting materials.

## 4. Materials and Methods

### General Information

All manipulations were carried out using standard Schlenk and glovebox techniques under an atmosphere of dry argon. Hexane, diethyl ether, toluene and THF were passed through columns containing activated alumina and molecular sieves and were degassed before use. Solvents were stored over potassium mirrors, with the exception of THF, which was stored over activated 4 Å molecular sieves. C_6_D_6_ (Cambridge Isotope Laboratories) was dried over potassium, vacuum-transferred and degassed by three freeze-pump-thaw cycles. {HN(Si^t^BuMe_2_)(SiMe_3_)} [13], {HN(Si^i^Pr_3_)(SiMe_3_)} [14], {HN(Si^t^BuMe_2_)_2_} [14], {HN(Si^i^Pr_3_)(Si^t^BuMe_2_)} [14], {HN(Si^i^Pr_3_)_2_} (**1**) [14] and ^n^BuNa [24] were prepared according to published procedures. ^n^BuLi (2.5 M in hexane) was transferred to a J. Young tap-appended ampoule and was used as received. NaH was purchased as a 60% dispersion in mineral oil and was washed three times with pentane and dried in vacuo before use. All other solid reagents were purchased and dried for 4 h in vacuo prior to use. ^1^H, ^13^C{^1^H}, ^29^Si{^1^H} and ^7^Li{^1^H}-NMR spectra were recorded on a Bruker DPX400 spectrometer operating at 400.1, 100.6, 79.5 and 155.5 MHz or a Bruker AV500 operating at 500.2, 125.8 and 99.4, respectively; chemical shifts are relative to TMS (^1^H, ^13^C, ^29^Si) or external aqueous 1.0 M LiCl solution (^7^Li). Most ATR-FTIR spectra were recorded as microcrystalline powders using a Bruker Tensor 27 spectrometer but the FTIR spectrum of **2** was recorded as a Nujol mull in KBr discs on a Perkin Elmer Spectrum RX1 spectrometer. Elemental microanalyses were carried out by Mr Stephen Boyer at London Metropolitan University Elemental Analysis Service or Mr Martin Jennings and Mrs Anne Davies at The University of Manchester School of Chemistry Microanalysis Service.

*Synthesis of [Li{μ-N(Si^t^BuMe_2_)(SiMe_3_)}]_2_* (**2**): ^n^BuLi (2.0 mL, 5.0 mmol, 2.5 M in hexane) was added dropwise to a pre-cooled (−78 °C) solution of {HN(Si^t^BuMe_2_)(SiMe_3_)} (1.00 g, 4.9 mmol) in hexane (10 mL). The pale yellow reaction mixture was slowly warmed to room temperature and stirred for 16 h. The solution was reduced in volume to ca. 2 mL in vacuo and stored at −20 °C for 16 h to give colourless blocks of **2** (0.16 g, 16%). Anal. Calcd. for C_18_H_48_N_2_Li_2_Si_4_: C, 51.63; H, 11.56; N, 6.69. Found: C, 51.50; H, 11.46; N, 6.57. Upon warming to room temperature, the crystals melted. THF (2 mL) was added and volatiles were removed *in vacuo* to give a white solid. NMR spectroscopic data was collected on the resultant THF adduct, with ^1^H integrals giving an empirical formula of [Li{μ-N(Si^t^BuMe_2_)(SiMe_3_)}(THF)]*_n_* (**2-THF**). ^1^H-NMR (400.1 MHz, C_6_D_6_, 298 K): δ 0.27 (s, 6H, Si^t^Bu(C*H*_3_)_2_), 0.35 (s, 9H, Si(C*H*_3_)_3_), 1.15 (s, 9H, Si(C(C*H*_3_)_3_), 1.28 (m, 4H, OCH_2_C*H*_2_), 3.57 (m, 4H, OC*H*_2_CH_2_). ^13^C{^1^H}-NMR (100.6 MHz, C_6_D_6_, 298 K): δ 1.79 (s, Si^t^Bu(*C*H_3_)_2_), 7.26 (s, Si(*C*H_3_)_3_), 20.74 (s, Si*C*(CH_3_)_3_), 25.47 (s, OCH_2_*C*H_2_), 29.02 (s, SiC(*C*H_3_)_3_), 69.17 (s, O*C*H_2_CH_2_). ^29^Si{^1^H}-NMR (99.4 MHz, C_6_D_6_, 298 K): δ −13.66 (s, *Si*^t^BuMe_2_), −5.89 (s, *Si*Me_3_). ^7^Li{^1^H}-NMR (155.5 MHz, C_6_D_6_, 298 K): δ 1.16 (s). FTIR (Nujol, $\bar{\nu }$/cm^−1^): 1176 (m), 933 (m), 867 (w), 839 (m), 719 (w), 666 (w).

*Synthesis of [Li{μ-N(Si^i^Pr_3_)(SiMe_3_)}]_2_* (**3**): ^n^BuLi (1.1 mL, 2.8 mmol, 2.5 M in hexane) was added dropwise to a pre-cooled (−78 °C) solution of {HN(Si^i^Pr_3_)(SiMe_3_)} (0.70 g, 2.9 mmol) in hexane (10 mL). The pale yellow reaction mixture was slowly warmed to room temperature, stirred for 16 h and volatiles were removed in vacuo. The resultant white solid was dissolved in hot hexane (2.3 mL) and stored at −20 °C for 16 h to give colourless blocks of **3** (0.553 g, 77%). Anal. Calcd. for C_24_H_60_Li_2_N_2_Si_4_: C, 57.32; H, 12.04; N, 5.57. Found: C, 57.18; H, 12.12; N, 5.61. ^1^H-NMR (500.2 MHz, C_6_D_6_, 298 K): δ 0.27 (s, 18H, Si(C*H*_3_)_3_), 0.99 (sept, 6H, *J*_HH_ = 7.5 Hz, SiC*H*(CH_3_)_2_), 1.15 (d, 36H, *J*_HH_ = 7.5 Hz, SiCH(C*H*_3_)_2_). ^13^C{^1^H}-NMR (125.8 MHz, C_6_D_6_, 298 K): δ 6.19 (s, Si(*C*H_3_)_3_), 16.34 (s, Si(*C*H(CH_3_)_2_), 20.21 (s, SiCH(*C*H_3_)_2_). ^29^Si{^1^H}-NMR (99.4 MHz, C_6_D_6_, 298 K): δ −12.03 (s, *Si*^i^Pr_3_), −1.38 (s, *Si*Me_3_). ^7^Li{^1^H}-NMR (155.5 MHz, C_6_D_6_, 298 K): δ 1.47 (s). FTIR (ATR, microcrystalline, $\bar{\nu }$/cm^−1^): 2938 (s), 2864 (s), 1466 (s), 1391 (m), 1249 (m), 1010 (m), 878 (m), 844 (s), 754 (m), 685 (s), 649 (s), 615 (s), 590 (s), 514 (s), 513 (s), 464 (s), 420 (s).

*Synthesis of [Li{N(Si^t^BuMe_2_)_2_}{μ-N(Si^t^BuMe_2_)_2_}Li(THF)]* (**4**) *and [Li_2_{μ-N(Si^t^BuMe_2_)_2_}(μ-^n^Bu)]_2_* (**5**): ^n^BuLi (10.2 mL, 25.5 mmol, 2.5 M in hexane) was added dropwise to a pre-cooled (−78 °C) solution of {HN(Si^t^BuMe_2_)_2_} (6.273 g, 25.5 mmol) in hexane (10 mL). The pale yellow reaction mixture was allowed to warm to room temperature and was stirred for 16 h. Volatiles were removed in vacuo in a 40 °C oil bath to remove any residual {HN(Si^t^BuMe_2_)_2_}. The resultant white solid was dissolved in pentane (5 mL) and THF (1 drop) and stored at −80 °C for 16 h to give colourless blocks of **4** (2.137 g, 29%). On one occasion during recrystallization of **4** several crystals of **5** formed but no other characterization data could be obtained due to the low yield. The sample of **4** was dried for 1 h in vacuo, which removed approximately half of the coordinated THF according to integrals in the ^1^H-NMR spectrum. Data for **4**: Anal. Calcd. for C_26_H_64_Li_2_N_2_O_0.5_Si_4_: C, 56.26; H, 11.62; N, 5.05. Found: C, 55.03; H, 11.95; N, 4.87. ^1^H-NMR (400.1 MHz, C_6_D_6_, 298 K): δ 0.23 (s, 24H, Si^t^Bu(C*H*_3_)_2_), 1.08 (s, 36H, SiC(C*H*_3_)_3_)), 1.24 (m, 2H, OCH_2_C*H*_2_), 3.19 (m, 2H, OC*H*_2_CH_2_). ^13^C{^1^H}-NMR (100.6 MHz, C_6_D_6_, 298 K): δ 1.65 (s, Si^t^Bu(*C*H_3_)_2_), 20.60 (s, Si*C*(CH_3_)_3_), 25.39 (OCH_2_*C*H_2_), 28.64 (s, SiC(*C*H_3_)_3_) 69.27 (O*C*H_2_CH_2_). ^29^Si{^1^H}-NMR (79.5 MHz, C_6_D_6_, 298 K): δ −6.15 (s, *Si*^t^BuMe_2_). ^7^Li{^1^H}-NMR (155.5 MHz, C_6_D_6_, 298 K): δ 1.05 (s). FTIR (ATR, microcrystalline, $\bar{\nu }$/cm^−1^): 2939 (m), 2851 (s), 1578 (s), 1467 (s), 1386 (s), 1358 (s), 1248 (s), 1205 (s), 1121 (s), 1034 (s), 1000 (s), 935 (s), 820 (s), 785 (m), 625 (s), 564 (s), 430 (s).

*Synthesis of [Li{N(Si^i^Pr_3_)(Si^t^BuMe_2_)}(DME)]* (**6**): ^n^BuLi (13.9 mL, 34.8 mmol, 2.5 M in hexane) was added dropwise to a pre-cooled (−78 °C) solution of {HN(Si^i^Pr_3_)(Si^t^BuMe_2_)} (9.112 g, 31.68 mmol) in diethyl ether (30 mL). The yellow reaction mixture was allowed to warm to room temperature and was stirred for 15 min. Volatiles were removed in vacuo in a 40 °C oil bath to remove residual {HN(Si^i^Pr_3_)(Si^t^BuMe_2_)}. Hexane (20 mL) was added to the resultant yellow-orange tacky solid to form an orange reaction mixture with a white precipitate. The mixture was stirred for 16 h, filtered and the remaining solids extracted with hexane (2 × 10 mL). The combined extracts were concentrated to ca. 20 mL and stored at −20 °C for 16 h to give a white powder. This was isolated and a portion (0.977 g) was dissolved in pentane (1 mL) with 1 drop of DME and stored at −20 °C to give several crystals of **6**.

*Synthesis of [Li{N(Si^i^Pr_3_)_2_}(THF)]* (**7**) *and [Li{N(Si^i^Pr_3_)_2_}(THF)_2_]* (**8**): ^n^BuLi (1.6 mL, 4.0 mmol, 2.5 M in hexane) was added dropwise to a pre-cooled (−78 °C) solution of {HN(Si^i^Pr_3_)_2_} (1.32 g, 4.0 mmol) in THF (20 mL). The yellow reaction mixture was allowed to warm to room temperature, stirred for 1 h and volatiles were removed in vacuo. Hexane (20 mL) was added to the resultant orange solid and the solution was filtered, concentrated to ca. 10 mL and stored at −20 °C for 16 h to give colourless blocks of **7** (0.0848 g, 10%). On one occasion, several crystals of **8** were observed. Data for **7**: Anal. Calcd. for C_22_H_50_LiNOSi_2_: C, 64.81; H, 12.37; N, 3.44. Found: C, 62.69; H, 12.61; N, 3.53. ^1^H-NMR (400.1 MHz, C_6_D_6_, 298 K): δ 1.02 (m, 4H, OCH_2_C*H*_2_), 1.15 (sept, 12H, *J*_HH_ = 7.5 Hz, SiC*H*(CH_3_)_2_), 1.34 (d, 36H, *J*_HH_ = 7.5 Hz, SiCH(C*H*_3_)_2_), 3.11 (m, 4H, OC*H*_2_CH_2_). ^13^C{^1^H}-NMR (100.6 MHz, C_6_D_6_, 298 K): δ 16.39 (s, Si*C*H(CH_3_)_2_), 20.52 (s, SiCH(*C*H_3_)_2_), 25.37 (s, OCH_2_*C*H_2_), 69.11 (s, O*C*H_2_CH_2_). ^29^Si{^1^H}-NMR (79.5 MHz, C_6_D_6_, 298 K): δ −10.39 (s, *Si*^i^Pr_3_). ^7^Li{^1^H}-NMR (155.5 MHz, C_6_D_6_, 298 K): δ 1.00 (s). FTIR (ATR, microcrystalline, $\bar{\nu }$/cm^−1^): 2940 (s), 2888 (s), 1462 (s), 1382 (s), 1243 (s), 1214 (s), 1110 (s), 1066 (s), 1032 (s), 1001 (s), 981 (s), 942 (s), 879 (s), 821 (s), 787 (s), 715 (m), 651 (m), 554 (m), 520 (s), 504 (s), 467 (s), 448 (s), 414 (s).

*Synthesis of [Na{N(Si^t^BuMe_2_)_2_}]_n_* (**9**) *and [Na{μ-N(Si^t^BuMe_2_)_2_}(THF)]_2_* (**9-THF**): {HN(Si^t^BuMe_2_)_2_} (20.095 g, 82.17 mmol) in toluene (10 mL) was added to a pre-cooled (−78 °C) suspension of NaH (2.357 g, 98.6 mmol) and 2 mol % of NaO^t^Bu (0.158 g, 1.64 mmol) in toluene (30 mL). The grey reaction mixture was allowed to warm to room temperature and stirred for 24 h. THF (10 mL) was added and the reaction mixture was refluxed for 16 h and filtered. Volatiles were removed in vacuo in a 40 °C oil bath to give a pale yellow oil. Pentane (20 mL) was added and the solution was stored at −20 °C for 16 h to give large colourless blocks of **9-THF** contaminated with {HN(Si^t^BuMe_2_)_2_}. Anal. Calcd. For C_32_H_76_N_2_O_2_Si_4_: C, 56.60; H, 11.29; N, 4.13. Found: C, 53.19; H, 11.50; N, 4.67. The crystals were heated in vacuo at 60 °C for 1 h to give a colourless oil, which solidified when cooled to room temperature. The resultant white solid was washed with pentane (40 mL) to give **9** as a white powder (6.477 g, 23%). Data for **9**: ^1^H-NMR (400.1 MHz, C_6_D_6_, 298 K): δ 0.09 (s, 12H, Si^t^Bu(C*H*_3_)_2_), 1.10 (s, 18H, SiC(C*H*_3_)_3_). ^13^C{^1^H}-NMR (100.6 MHz, C_6_D_6_, 298 K): δ 2.62 (s, Si^t^Bu(*C*H_3_)_2_), 20.57 (s, Si*C*(CH_3_)_3_), 28.69 (s, SiC(*C*H_3_)_3_). ^29^Si{^1^H}-NMR (79.5 MHz, C_6_D_6_, 298 K): δ −11.67 (s, *Si*^i^Pr_3_). FTIR (ATR, microcrystalline, $\bar{\nu }$/cm^−1^): 2941 (m), 2890 (s), 2850 (s), 1470 (m), 1385 (s), 1348 (m), 1247 (s), 1199 (s), 1059 (s), 1002 (s), 935 (s), 821 (s), 802 (s), 783 (s), 747 (s), 644 (s), 605 (s), 565 (s), 523 (s), 419 (s).

*Synthesis of [Na{N(Si^i^Pr_3_)_2_}(C_7_H_8_)] (**10**)*: {HN(Si^i^Pr_3_)_2_} (0.99 g, 3.0 mmol) in hexane (10 mL) was added dropwise to a precooled (−78 °C) solution of ^n^BuNa (0.24 g, 3.0 mmol) in hexane (10 mL). The reaction mixture was allowed to warm to room temperature and was stirred overnight. Volatiles were removed in vacuo and the resultant white solid was extracted with toluene (10 mL) and filtered. The solution was concentrated to ca. 1 mL and stored at −20 °C for 16 h to give colourless needles of **10** (0.043 g, 3%). These crystals were dried for 1 h in vacuo, which removed approximately half of the coordinated toluene according to integrals in the ^1^H-NMR spectrum. Anal. Calcd. for C_21.5_H_46_NNaSi_2_: C, 64.92; H, 11.66; N, 3.52. Found: C, 64.85; H, 11.81; N, 3.17. ^1^H-NMR (400.1 MHz, C_6_D_6_, 298 K): δ 0.92 (br, 6H, SiC*H*(CH_3_)_2_), 1.22 (br, 36H, SiCH(C*H*_3_)_2_), 2.11 (br, 1.5H, C*H*_3_Ph), 7.03 (br, 2.5H, Ph-*H*). ^13^C{^1^H}-NMR (100.6 MHz, C_6_D_6_, 298 K): δ 16.87 (s, Si*C*H(CH_3_)_2_), 19.34 (s, *C*H_3_Ph), 20.62 (s, SiCH(*C*H_3_)_2_), Ph-*C* not observed. ^29^Si{^1^H}-NMR (79.5 MHz, C_6_D_6_, 298 K): δ −13.82 (s, *Si*^i^Pr_3_). FTIR (ATR, microcrystalline, $\bar{\nu }$/cm^−1^): 2912 (m), 2802 (s), 1461 (s), 1382 (m), 1240 (s), 1134 (s), 1067 (s), 1040 (s), 1000 (s), 979 (s), 943 (s), 914 (s), 878 (s), 746 (s), 718 (s), 655 (s), 628 (s), 611 (s), 541 (s), 504 (s), 457 (s), 428 (s), 407 (s).

## Figures and Tables

**Table 1:** Selected bond lengths (Å) and angles (°) for **1**–5 and **6**–**10** (M = Li, **6**–**8**; Na, **9**–**10**).

**Distance (Å)/Angle (°)**	**1**	**2**	**3**	**4**	**5**
N(1)–Si(1)	1.7348(12)	1.701(2)	1.711(2)	1.712(4)	1.714(4)
N(1)–Si(2)	1.7370(12)	1.704(2)	1.707(2)	1.728(4)	1.713(5)
Li(1)–N(1)	-	2.015(5)	2.022(4)	1.974(10)	1.986(9)
Li(1)–N(2)	-	1.986(5)	2.032(4)	-	-
Li(2)–N(1)	-	1.982(5)	2.036(4)	2.026(8)	1.946(12)
Li(2)–N(2)	-	2.023(5)	2.030(4)	1.915(9)	-
N(2)–Si(3)	-	1.706(2)	1.708(2)	1.667(4)	-
N(2)–Si(4)	-	1.701(2)	1.705(2)	1.679(3)	-
Li(1)–O(1)	-	-	-	1.891(8)	-
Li(1)–C(13)	-	-	-	-	2.037(9)
Li···C distances < 2.5 Å	-	2.378(6)	2.378(6)	-	-
	-	2.392(7)	2.392(7)	-	-
	-	2.393(7)	-	-	-
	-	2.427(6)	-	-	-
Si(1)–N(1)–Si(2)	145.43(8)	125.33(12)	127.91(11)	121.0(2)	131.0(3)
Si(3)–N(2)–Si(4)	-	125.29(13)	127.87(11)	144.2(2)	-
N(1)–Li(1)–N(2)	-	109.1(2)	110.1(2)	-	
N(1)–Li(2)–N(2)	-	109.0(2)	110.0(2)	160.2(4)	-
Li(1)–N(1)–Li(2)	-	71.0(2)	69.8(2)	97.6(3)	95.7(5)
Li(1)–N(2)–Li(2)	-	70.8(2)	69.5(2)	-	-
N(1)–Li(1)–O(1)	-	-	-	155.4(5)	-
N(1)–Li(1)–C(13)	-	-	-	-	171.9(5)
N(1A)–Li(2A)–C(13)	-	-	-	-	173.9(7)
Li(1)–C(13)–Li(2A)	-	-	-	-	91.7(4)
**Distance (Å)/Angle (°)**	**6**	**7**	**8**	**9-THF**	**10**
N(1)–Si(1)	1.681(2)	1.6761(13)	1.686(2)	1.697(3)	1.6709(12)
N(1)–Si(2)	1.679(5)	1.6805(13)	1.681(2)	1.702(3)	1.6710(13)
M(1)–N(1)	1.894(13)	1.893(4)	1.953(5)	2.490(2)	2.2608(14)
M(1)–N(2)	-	-	-	-	-
M(2)–N(1)	-	-	-	-	-
M(2)–N(2)	-	-	-	-	-
M(1)–O(1)	1.984(5)	1.866(3)	1.987(5)	2.328(3)	-
M(1)–O(2)	1.958(3)	-	1.957(5)	-	-
Na(1)···C(19)	-	-	-	-	2.912(2)
Na(1)···C(20)	-	-	-	-	2.815(2)
Na(1)···C(21)	-	-	-	-	2.801(2)
Na(1)···C(22)	-	-	-	-	2.870(2)
Na(1)···C(23)	-	-	-	-	2.935(2)
Na(1)···C(24)	-	-	-	-	2.956(2)
Na(1)···Ph_centroid_	-	-	-	-	2.527(2)
Si(1)–N(1)–Si(2)	135.08(13)	142.07(8)	143.06(13)	124.18(14)	144.09(8)
N(1)–Na(1)–N(1A)	-	-	-	108.47(7)	-
Na(1)–N(1)–Na(1A)	-	-	-	71.53(7)	-
N(1)–M(1)–O(1)	143.4(3)	168.7(2)	132.5(3)	126.51(7)	-
N(1)–M(1)–O(2)	133.6(2)	-	130.7(3)	-	-
O(1)–M(1)–O(2)	82.8(2)	-	96.6(2)	-	-

## Supplementary Materials

The following are available online, Figures S1–S28: NMR and FTIR spectra of **2**–**4**, **7**, **9** and **10**, Figure S29: solid state structure of **4**, Tables S1–S4: Selected crystallographic data for **1**–**10**. Research data files supporting this publication are available from Mendeley Data at doi:10.17632/h4nvm4xsx5.1. CCDC 1832798-1832807 contain the supplementary crystal data for this article. These data can be obtained free of charge from the Cambridge Crystallographic Data Centre via www.ccdc.cam.ac.uk/data_request/cif.
