# Supplementary material for: Structural Characterization of Lithium and Sodium Bulky Bis(silyl)amide Complexes
Source: Molecules. 2018 May 10;23(5):1138. doi: 10.3390/molecules23051138 (PMC6100173; doi:10.3390/molecules23051138)

checkCIF/PLATON report

Structure factors have been supplied for datablock(s) 1, 10, 2, 3, 4, 5, 6, 7, 8, 9-THF

THIS REPORT IS FOR GUIDANCE ONLY. IF USED AS PART OF A REVIEW PROCEDURE FOR PUBLICATION, IT SHOULD NOT REPLACE THE EXPERTISE OF AN EXPERIENCED CRYSTALLOGRAPHIC REFEREE.

No syntax errors found. CIF dictionary Interpreting this report

Datablock: 1

```
Bond precision:  C-C = 0.0020 A          Wavelength=1.54184

Cell:           a=8.81051(9)    b=13.36759(16)      c=18.58518(19)
                alpha=90        beta=99.1278(10)    gamma=90
Temperature:    150 K

                 Calculated                 Reported
Volume           2161.16(4)                 2161.16(4)
Space group      P 21/n                     P 1 21/n 1
Hall group       -P 2yn                     -P 2yn
Moiety formula   C18 H43 N Si2              C18 H43 N Si2
Sum formula      C18 H43 N Si2              C18 H43 N Si2
Mr               329.71                     329.71
Dx,g cm-3        1.013                      1.013
Z                4                          4
Mu (mm-1)        1.436                      1.436
F000             744.0                      744.0
F000'            747.36
h,k,lmax         10,16,22                   10,16,22
Nref             3965                       3938
Tmin,Tmax        0.866,0.931                0.279,1.000
Tmin'            0.866

Correction method= # Reported T Limits: Tmin=0.279 Tmax=1.000
AbsCorr = MULTI-SCAN

Data completeness= 0.993          Theta(max)= 68.229

R(reflections)= 0.0399( 3762)     wR2(reflections)= 0.1124( 3938)

S = 1.066                  Npar= 209
```

The following ALERTS were generated. Each ALERT has the format
test-name_ALERT_alert-type_alert-level.
Click on the hyperlinks for more details of the test.

Alert level C

```
PLAT420_ALERT_2_C D-H Without Acceptor       N1       --H1             Please Check
PLAT911_ALERT_3_C Missing FCF Refl Between Thmin & STh/L=    0.600          22 Report
PLAT918_ALERT_3_C Reflection(s) with I(obs) much Smaller I(calc) .           3 Check
PLAT922_ALERT_1_C wR2 in the CIF and FCF Differ by ..............       0.0013 Check
PLAT923_ALERT_1_C S    Values in the CIF and FCF Differ by .......       0.013 Check
PLAT939_ALERT_3_C Large Value of Not (SHELXL) Weight Optimized S .       13.57 Check
```

Alert level G

```
PLAT002_ALERT_2_G Number of Distance or Angle Restraints on AtSite           5 Note
PLAT007_ALERT_5_G Number of Unrefined Donor-H Atoms .............            1 Report
PLAT176_ALERT_4_G The CIF-Embedded .res File Contains SADI Records           2 Report
PLAT301_ALERT_3_G Main Residue  Disorder .............(Resd  1  )          10% Note
PLAT860_ALERT_3_G Number of Least-Squares Restraints ............            2 Note
PLAT912_ALERT_4_G Missing # of FCF Reflections Above STh/L=  0.600           5 Note
PLAT978_ALERT_2_G Number C-C Bonds with Positive Residual Density.           6 Info
```

```
   0 ALERT level A = Most likely a serious problem - resolve or explain
   0 ALERT level B = A potentially serious problem, consider carefully
   6 ALERT level C = Check. Ensure it is not caused by an omission or oversight
   7 ALERT level G = General information/check it is not something unexpected

   2 ALERT type 1 CIF construction/syntax error, inconsistent or missing data
   3 ALERT type 2 Indicator that the structure model may be wrong or deficient
   5 ALERT type 3 Indicator that the structure quality may be low
   2 ALERT type 4 Improvement, methodology, query or suggestion
   1 ALERT type 5 Informative message, check
```

Datablock: 2

```
Bond precision:   C-C = 0.0043 A          Wavelength=0.71073

Cell:           a=10.2374(8)       b=11.9719(8)      c=12.6730(7)
                alpha=83.508(5)    beta=76.177(6)    gamma=68.182(7)
Temperature:    100 K
```

	Calculated	Reported
Volume	1399.69(18)	1399.69(18)
Space group	P -1	P -1
Hall group	-P 1	-P 1
Moiety formula	C18 H48 Li2 N2 Si4	C18 H48 Li2 N2 Si4
Sum formula	C18 H48 Li2 N2 Si4	C18 H48 Li2 N2 Si4
Mr	418.82	418.82
Dx,g cm-3	0.994	0.994
Z	2	2
Mu (mm-1)	0.217	0.217
F000	464.0	464.0
F000'	464.72	
h,k,lmax	12,14,15	12,14,15
Nref	5139	5102
Tmin,Tmax	0.950,0.970	0.921,0.967
Tmin'	0.916	

Correction method= # Reported T Limits: Tmin=0.921 Tmax=0.967
AbsCorr = ANALYTICAL

Data completeness= 0.993 Theta(max)= 25.348

R(reflections)= 0.0505(3815) wR2(reflections)= 0.1214(5102)

S = 1.034 Npar= 282

The following ALERTS were generated. Each ALERT has the format
test-name_ALERT_alert-type_alert-level.
Click on the hyperlinks for more details of the test.

Alert level B

PLAT049_ALERT_1_B Calculated Density Less Than 1.0 gcm-3 .... 0.9938 Check

Author Response: This compound is a Li salt. Densities below 1.0 are a common problem for compounds such as this. Inspection of the data with PLATON suggests no missing symmetry elements.

Alert level C

PLAT241_ALERT_2_C High 'MainMol' Ueq as Compared to Neighbors of C7 Check
PLAT340_ALERT_3_C Low Bond Precision on C-C Bonds .... 0.00433 Ang.
PLAT906_ALERT_3_C Large K Value in the Analysis of Variance ...... 2.134 Check
PLAT910_ALERT_3_C Missing # of FCF Reflection(s) Below Theta(Min). 5 Note
PLAT911_ALERT_3_C Missing FCF Refl Between Thmin & STh/L= 0.600 28 Report

---

Alert level G

PLAT002_ALERT_2_G Number of Distance or Angle Restraints on AtSite 11 Note
PLAT003_ALERT_2_G Number of Uiso or Uij Restrained non-H Atoms ... 7 Report

```
PLAT066_ALERT_1_G Predicted and Reported Tmin&Tmax Range Identical          ? Check
PLAT176_ALERT_4_G The CIF-Embedded .res File Contains SADI Records          1 Report
PLAT178_ALERT_4_G The CIF-Embedded .res File Contains SIMU Records          1 Report
PLAT301_ALERT_3_G Main Residue  Disorder ..............(Resd  1  )        12% Note
PLAT343_ALERT_2_G Unusual          Angle Range in Main Residue for         C5 Check
PLAT343_ALERT_2_G Unusual          Angle Range in Main Residue for         C7 Check
PLAT343_ALERT_2_G Unusual          Angle Range in Main Residue for        C14 Check
PLAT343_ALERT_2_G Unusual          Angle Range in Main Residue for        C16 Check
PLAT793_ALERT_4_G Model has Chirality at Si1         (Centro SPGR)          S Verify
PLAT860_ALERT_3_G Number of Least-Squares Restraints .............         90 Note
PLAT912_ALERT_4_G Missing # of FCF Reflections Above STh/L=  0.600          5 Note
PLAT978_ALERT_2_G Number C-C Bonds with Positive Residual Density.          1 Info
```

```
   0 ALERT level A = Most likely a serious problem - resolve or explain
   1 ALERT level B = A potentially serious problem, consider carefully
   5 ALERT level C = Check. Ensure it is not caused by an omission or oversight
  14 ALERT level G = General information/check it is not something unexpected

   2 ALERT type 1 CIF construction/syntax error, inconsistent or missing data
   8 ALERT type 2 Indicator that the structure model may be wrong or deficient
   6 ALERT type 3 Indicator that the structure quality may be low
   4 ALERT type 4 Improvement, methodology, query or suggestion
   0 ALERT type 5 Informative message, check
```

Datablock: 3

```
Bond precision:  C-C = 0.0037 A          Wavelength=0.71073

Cell:           a=12.4492(4)      b=16.4469(6)     c=17.6144(6)
                alpha=67.932(3)   beta=80.537(3)   gamma=89.926(3)
Temperature:    150 K

                 Calculated                 Reported
Volume           3289.6(2)                  3289.7(2)
Space group      P -1                       P -1
Hall group       -P 1                       -P 1
Moiety formula   C24 H60 Li2 N2 Si4         C24 H60 Li2 N2 Si4
Sum formula      C24 H60 Li2 N2 Si4         C24 H60 Li2 N2 Si4
Mr               502.98                     502.98
Dx,g cm-3        1.016                      1.016
Z                4                          4
Mu (mm-1)        0.194                      0.194
F000             1120.0                     1120.0
F000’            1121.50
h,k,lmax         14,19,21                   14,19,21
Nref             12032                      11975
Tmin,Tmax        0.940,0.968                0.365,1.000
Tmin’            0.895
```

Correction method= # Reported T Limits: Tmin=0.365 Tmax=1.000
AbsCorr = GAUSSIAN

Data completeness= 0.995 Theta(max)= 25.349

R(reflections)= 0.0443(9260) wR2(reflections)= 0.1082(11975)

S = 1.031 Npar= 613

The following ALERTS were generated. Each ALERT has the format
test-name_ALERT_alert-type_alert-level.
Click on the hyperlinks for more details of the test.

Alert level C

```
PLAT094_ALERT_2_C Ratio of Maximum / Minimum Residual Density ....       2.06 Report
PLAT230_ALERT_2_C Hirshfeld Test Diff for   Si1      --C1        .        5.6 s.u.
PLAT230_ALERT_2_C Hirshfeld Test Diff for   C1       --C2        .        5.7 s.u.
PLAT241_ALERT_2_C High   'MainMol' Ueq as Compared to Neighbors of         C2 Check
PLAT910_ALERT_3_C Missing # of FCF Reflection(s) Below Theta(Min).          5 Note
PLAT911_ALERT_3_C Missing FCF Refl Between Thmin & STh/L=    0.600         48 Report
PLAT978_ALERT_2_C Number C-C Bonds with Positive Residual Density.          0 Info
```

Alert level G

```
PLAT154_ALERT_1_G The s.u.'s on the Cell Angles are Equal ..(Note)      0.003 Degree
PLAT303_ALERT_2_G Full Occupancy Atom H2C       with # Connections       2.00 Check
PLAT303_ALERT_2_G Full Occupancy Atom H10C      with # Connections       2.00 Check
PLAT303_ALERT_2_G Full Occupancy Atom H14C      with # Connections       2.00 Check
PLAT303_ALERT_2_G Full Occupancy Atom H22C      with # Connections       2.00 Check
PLAT303_ALERT_2_G Full Occupancy Atom H38A      with # Connections       2.00 Check
PLAT343_ALERT_2_G Unusual          Angle Range in Main Residue for        C10 Check
PLAT343_ALERT_2_G Unusual          Angle Range in Main Residue for        C22 Check
PLAT793_ALERT_4_G Model has Chirality at C1          (Centro SPGR)          S Verify
PLAT793_ALERT_4_G Model has Chirality at C13         (Centro SPGR)          S Verify
PLAT793_ALERT_4_G Model has Chirality at C37         (Centro SPGR)          R Verify
PLAT912_ALERT_4_G Missing # of FCF Reflections Above STh/L=  0.600          5 Note
PLAT913_ALERT_3_G Missing # of Very Strong Reflections in FCF ....          1 Note
```

---

0 **ALERT level A** = Most likely a serious problem - resolve or explain
0 **ALERT level B** = A potentially serious problem, consider carefully
7 **ALERT level C** = Check. Ensure it is not caused by an omission or oversight
13 **ALERT level G** = General information/check it is not something unexpected

1 ALERT type 1 CIF construction/syntax error, inconsistent or missing data
12 ALERT type 2 Indicator that the structure model may be wrong or deficient
3 ALERT type 3 Indicator that the structure quality may be low
4 ALERT type 4 Improvement, methodology, query or suggestion
0 ALERT type 5 Informative message, check

Datablock: 4

Bond precision: C-C = 0.0071 A Wavelength=0.71073

Cell:	a=16.2998(17)	b=12.2405(7)	c=20.6644(19)
	alpha=90	beta=112.441(12)	gamma=90
Temperature:	100 K		

	Calculated	Reported
Volume	3810.7(7)	3810.7(7)
Space group	P 21/c	P 1 21/c 1
Hall group	-P 2ybc	-P 2ybc
Moiety formula	C28 H68 Li2 N2 O Si4	C28 H68 Li2 N2 O Si4
Sum formula	C28 H68 Li2 N2 O Si4	C28 H68 Li2 N2 O Si4
Mr	575.08	575.08
Dx,g cm-3	1.002	1.002
Z	4	4
Mu (mm-1)	0.176	0.176
F000	1280.0	1280.0
F000'	1281.58	
h,k,lmax	19,14,24	19,14,24
Nref	6973	5777
Tmin,Tmax	0.943,0.968	0.167,1.000
Tmin'	0.911	

Correction method= # Reported T Limits: Tmin=0.167 Tmax=1.000
AbsCorr = MULTI-SCAN

Data completeness= 0.828 Theta(max)= 25.349

R(reflections)= 0.0714(3543) wR2(reflections)= 0.1846(5777)

S = 1.001 Npar= 354

The following ALERTS were generated. Each ALERT has the format
test-name_ALERT_alert-type_alert-level.
Click on the hyperlinks for more details of the test.

Alert level A

PLAT029_ALERT_3_A _diffrn_measured_fraction_theta_full value Low . 0.831 Why?

Author Response: Incorrect symmetry identified during pre-experiment (orthorhombic vs. monoclinic) which was not identified until after collection. The sensitivity of the compound precluded recollection and it is included only for completeness in the publication to show the identity of the compound.

Alert level B

PLAT911_ALERT_3_B Missing FCF Refl Between Thmin & STh/L= 0.600 1162 Report

Author Response: Incorrect symmetry identified during pre-experiment (orthorhombic vs. monoclinic) which was not identified until after collection. The sensitivity of the compound precluded recollection and it is included only for completeness in the publication to show the identity of the compound.

Alert level C

```
PLAT340_ALERT_3_C Low Bond Precision on  C-C Bonds ...............    0.00707 Ang.
PLAT906_ALERT_3_C Large K Value in the Analysis of Variance ......      2.281 Check
PLAT910_ALERT_3_C Missing # of FCF Reflection(s) Below Theta(Min).          6 Note
PLAT978_ALERT_2_C Number C-C Bonds with Positive Residual Density.          0 Info
```

Alert level G

```
PLAT912_ALERT_4_G Missing # of FCF Reflections Above STh/L=  0.600         29 Note
```

```
   1 ALERT level A = Most likely a serious problem - resolve or explain
   1 ALERT level B = A potentially serious problem, consider carefully
   4 ALERT level C = Check. Ensure it is not caused by an omission or oversight
   1 ALERT level G = General information/check it is not something unexpected

   0 ALERT type 1 CIF construction/syntax error, inconsistent or missing data
   1 ALERT type 2 Indicator that the structure model may be wrong or deficient
   5 ALERT type 3 Indicator that the structure quality may be low
   1 ALERT type 4 Improvement, methodology, query or suggestion
   0 ALERT type 5 Informative message, check
```

Datablock: 5

```
Bond precision:   C-C = 0.0077 A          Wavelength=0.71073

Cell:          a=9.1355(11)       b=11.0906(18)     c=12.644(2)
               alpha=63.996(16)   beta=77.951(12)   gamma=68.950(13)
Temperature:   150 K
```

	Calculated	Reported
Volume	1072.5(3)	1072.5(3)
Space group	P -1	P -1
Hall group	-P 1	-P 1
Moiety formula	C32 H78 Li4 N2 Si4	C32 H78 Li4 N2 Si4
Sum formula	C32 H78 Li4 N2 Si4	C32 H78 Li4 N2 Si4
Mr	631.08	631.08
Dx,g cm-3	0.977	0.977
Z	1	1
Mu (mm-1)	0.159	0.159
F000	352.0	352.0
F000'	352.39	
h,k,lmax	11,13,15	11,13,15
Nref	3913	3865
Tmin,Tmax	0.952,0.973	0.580,1.000
Tmin'	0.948	

Correction method= # Reported T Limits: Tmin=0.580 Tmax=1.000
AbsCorr = GAUSSIAN

Data completeness= 0.988 Theta(max)= 25.350

R(reflections)= 0.0806(2719) wR2(reflections)= 0.2168(3865)

S = 1.117 Npar= 218

The following ALERTS were generated. Each ALERT has the format
test-name_ALERT_alert-type_alert-level.
Click on the hyperlinks for more details of the test.

Alert level B

PLAT049_ALERT_1_B Calculated Density Less Than 1.0 gcm-3 .... 0.9771 Check

Author Response: This compound is a Li salt. Densities below 1.0 are a common problem for compounds such as this. Inspection of the data with PLATON suggests no missing symmetry elements.

Alert level C

```
PLAT340_ALERT_3_C Low Bond Precision on  C-C Bonds ...............    0.00767 Ang.
PLAT906_ALERT_3_C Large K Value in the Analysis of Variance ......      8.651 Check
PLAT906_ALERT_3_C Large K Value in the Analysis of Variance ......      2.055 Check
PLAT911_ALERT_3_C Missing FCF Refl Between Thmin & STh/L=    0.600        47 Report
PLAT934_ALERT_3_C Number of (Iobs-Icalc)/SigmaW > 10 Outliers ....         1 Check
PLAT977_ALERT_2_C Check Negative Difference Density on H13D           -0.48 eA-3
PLAT977_ALERT_2_C Check Negative Difference Density on H13B           -0.47 eA-3
PLAT978_ALERT_2_C Number C-C Bonds with Positive Residual Density.        0 Info
```

Alert level G

```
PLAT002_ALERT_2_G Number of Distance or Angle Restraints on AtSite          7 Note
PLAT003_ALERT_2_G Number of Uiso or Uij Restrained non-H Atoms ...          7 Report
PLAT176_ALERT_4_G The CIF-Embedded .res File Contains SADI Records          3 Report
PLAT178_ALERT_4_G The CIF-Embedded .res File Contains SIMU Records          1 Report
PLAT301_ALERT_3_G Main Residue  Disorder ..............(Resd  1  )        14% Note
PLAT860_ALERT_3_G Number of Least-Squares Restraints .............         81 Note
PLAT910_ALERT_3_G Missing # of FCF Reflection(s) Below Theta(Min).          1 Note
```

---

0 **ALERT level A** = Most likely a serious problem - resolve or explain
1 **ALERT level B** = A potentially serious problem, consider carefully
8 **ALERT level C** = Check. Ensure it is not caused by an omission or oversight
7 **ALERT level G** = General information/check it is not something unexpected

1 ALERT type 1 CIF construction/syntax error, inconsistent or missing data
5 ALERT type 2 Indicator that the structure model may be wrong or deficient
8 ALERT type 3 Indicator that the structure quality may be low
2 ALERT type 4 Improvement, methodology, query or suggestion
0 ALERT type 5 Informative message, check

Datablock: 6

Bond precision: C-C = 0.0043 A Wavelength=0.71073

Cell:	a=17.6846(12)	b=9.6929(6)	c=30.5856(17)
	alpha=90	beta=104.603(7)	gamma=90
Temperature:	150 K		

	Calculated	Reported
Volume	5073.5(6)	5073.5(6)
Space group	P 21/c	P 1 21/c 1
Hall group	-P 2ybc	-P 2ybc
Moiety formula	C19 H46 Li N O2 Si2	C19 H46 Li N O2 Si2
Sum formula	C19 H46 Li N O2 Si2	C19 H46 Li N O2 Si2
Mr	383.69	383.69
Dx,g cm-3	1.005	1.005
Z	8	8
Mu (mm-1)	0.150	0.150
F000	1712.0	1712.0
F000’	1713.79	
h,k,lmax	22,12,38	22,12,38
Nref	10398	10309
Tmin,Tmax	0.982,0.985	0.449,1.000
Tmin’	0.956	

Correction method= # Reported T Limits: Tmin=0.449 Tmax=1.000
AbsCorr = MULTI-SCAN

```
Data completeness= 0.991          Theta(max)= 26.396

R(reflections)= 0.0571( 6928)     wR2(reflections)= 0.1419( 10309)

S = 1.020                Npar= 477
```

The following ALERTS were generated. Each ALERT has the format
test-name_ALERT_alert-type_alert-level.
Click on the hyperlinks for more details of the test.

Alert level C

```
PLAT241_ALERT_2_C High   'MainMol' Ueq as Compared to Neighbors of        C17 Check
PLAT340_ALERT_3_C Low Bond Precision on  C-C Bonds ...............    0.00425 Ang.
PLAT360_ALERT_2_C Short  C(sp3)-C(sp3) Bond  C17      - C18      .       1.42 Ang.
PLAT906_ALERT_3_C Large K Value in the Analysis of Variance ......      4.448 Check
PLAT911_ALERT_3_C Missing FCF Refl Between Thmin & STh/L=    0.600         41 Report
PLAT921_ALERT_1_C R1  in the CIF and FCF Differ by ...............     0.0015 Check
PLAT922_ALERT_1_C wR2 in the CIF and FCF Differ by ...............     0.0031 Check
PLAT923_ALERT_1_C S    Values in the CIF and FCF Differ by .......      0.023 Check
PLAT977_ALERT_2_C Check Negative Difference Density on H17B             -0.35 eA-3
PLAT978_ALERT_2_C Number C-C Bonds with Positive Residual Density.          0 Info
```

Alert level G

```
PLAT003_ALERT_2_G Number of Uiso or Uij Restrained non-H Atoms ...         50 Report
PLAT187_ALERT_4_G The CIF-Embedded .res File Contains RIGU Records          1 Report
PLAT860_ALERT_3_G Number of Least-Squares Restraints .............        396 Note
PLAT910_ALERT_3_G Missing # of FCF Reflection(s) Below Theta(Min).          4 Note
PLAT912_ALERT_4_G Missing # of FCF Reflections Above STh/L=  0.600         44 Note
PLAT913_ALERT_3_G Missing # of Very Strong Reflections in FCF ....          1 Note
```

---

0 **ALERT level A** = Most likely a serious problem - resolve or explain
0 **ALERT level B** = A potentially serious problem, consider carefully
10 **ALERT level C** = Check. Ensure it is not caused by an omission or oversight
6 **ALERT level G** = General information/check it is not something unexpected

3 ALERT type 1 CIF construction/syntax error, inconsistent or missing data
5 ALERT type 2 Indicator that the structure model may be wrong or deficient
6 ALERT type 3 Indicator that the structure quality may be low
2 ALERT type 4 Improvement, methodology, query or suggestion
0 ALERT type 5 Informative message, check

Datablock: 7

```
Bond precision:  C-C = 0.0024 A          Wavelength=1.54184

Cell:           a=16.30145(13)   b=20.68971(16)     c=16.01395(13)
                alpha=90         beta=100.9135(8)   gamma=90
Temperature:    100 K
```

	Calculated	Reported
Volume	5303.38(7)	5303.38(7)
Space group	P 21/c	P 1 21/c 1
Hall group	-P 2ybc	-P 2ybc
Moiety formula	C22 H50 Li N O Si2	C22 H50 Li N O Si2
Sum formula	C22 H50 Li N O Si2	C22 H50 Li N O Si2
Mr	407.75	407.75
Dx,g cm-3	1.021	1.021
Z	8	8
Mu (mm-1)	1.272	1.272
F000	1824.0	1824.0
F000'	1831.66	
h,k,lmax	19,24,19	19,24,19
Nref	9708	9689
Tmin,Tmax	0.738,0.835	0.492,1.000
Tmin'	0.570	

Correction method= # Reported T Limits: Tmin=0.492 Tmax=1.000
AbsCorr = GAUSSIAN

Data completeness= 0.998 Theta(max)= 68.247

R(reflections)= 0.0395(8531) wR2(reflections)= 0.1080(9689)

S = 1.048 Npar= 528

The following ALERTS were generated. Each ALERT has the format
test-name_ALERT_alert-type_alert-level.
Click on the hyperlinks for more details of the test.

Alert level C

```
PLAT220_ALERT_2_C Non-Solvent  Resd 2  C   Ueq(max)/Ueq(min) Range        4.0 Ratio
PLAT241_ALERT_2_C High   'MainMol' Ueq as Compared to Neighbors of        Li1 Check
PLAT241_ALERT_2_C High   'MainMol' Ueq as Compared to Neighbors of        C42 Check
PLAT242_ALERT_2_C Low    'MainMol' Ueq as Compared to Neighbors of        C41 Check
PLAT412_ALERT_2_C Short Intra XH3 .. XHn     H18A     ..H10B             1.88 Ang.
PLAT911_ALERT_3_C Missing FCF Refl Between Thmin & STh/L=    0.600         14 Report
PLAT977_ALERT_2_C Check Negative Difference Density on H42B             -0.36 eA-3
PLAT977_ALERT_2_C Check Negative Difference Density on H43A             -0.36 eA-3
PLAT978_ALERT_2_C Number C-C Bonds with Positive Residual Density.          0 Info
```

Alert level G

```
PLAT002_ALERT_2_G Number of Distance or Angle Restraints on AtSite          5 Note
PLAT003_ALERT_2_G Number of Uiso or Uij Restrained non-H Atoms ...         56 Report
PLAT142_ALERT_4_G s.u. on b - Axis Small or Missing .............     0.00016 Ang.
PLAT143_ALERT_4_G s.u. on c - Axis Small or Missing .............     0.00013 Ang.
PLAT176_ALERT_4_G The CIF-Embedded .res File Contains SADI Records          2 Report
PLAT187_ALERT_4_G The CIF-Embedded .res File Contains RIGU Records          1 Report
PLAT301_ALERT_3_G Main Residue  Disorder ..............(Resd  1  )         7% Note
PLAT860_ALERT_3_G Number of Least-Squares Restraints .............        408 Note
PLAT912_ALERT_4_G Missing # of FCF Reflections Above STh/L=  0.600          6 Note
```

PLAT913_ALERT_3_G Missing # of Very Strong Reflections in FCF .... 1 Note

---

0 **ALERT level A** = Most likely a serious problem - resolve or explain
0 **ALERT level B** = A potentially serious problem, consider carefully
9 **ALERT level C** = Check. Ensure it is not caused by an omission or oversight
10 **ALERT level G** = General information/check it is not something unexpected

0 ALERT type 1 CIF construction/syntax error, inconsistent or missing data
10 ALERT type 2 Indicator that the structure model may be wrong or deficient
4 ALERT type 3 Indicator that the structure quality may be low
5 ALERT type 4 Improvement, methodology, query or suggestion
0 ALERT type 5 Informative message, check

Datablock: 8

Bond precision: C-C = 0.0044 A Wavelength=0.71073

Cell:	a=10.5111(5)	b=15.9508(6)	c=18.7477(10)
	alpha=85.086(4)	beta=84.850(4)	gamma=83.646(4)
Temperature:	150 K		

	Calculated	Reported
Volume	3102.2(3)	3102.2(3)
Space group	P -1	P -1
Hall group	-P 1	-P 1
Moiety formula	C26 H58 Li N O2 Si2	C26 H58 Li N O2 Si2
Sum formula	C26 H58 Li N O2 Si2	C26 H58 Li N O2 Si2
Mr	479.85	479.85
Dx,g cm-3	1.027	1.027
Z	4	4
Mu (mm-1)	0.134	0.134
F000	1072.0	1072.0
F000’	1072.96	
h,k,lmax	12,19,22	12,19,22
Nref	11366	11272
Tmin,Tmax	0.944,0.971	0.881,0.933
Tmin’	0.944	

Correction method= # Reported T Limits: Tmin=0.881 Tmax=0.933
AbsCorr = ANALYTICAL

Data completeness= 0.992 Theta(max)= 25.350

R(reflections)= 0.0576(8029) wR2(reflections)= 0.1545(11272)

S = 1.025 Npar= 676

The following ALERTS were generated. Each ALERT has the format
test-name_ALERT_alert-type_alert-level.
Click on the hyperlinks for more details of the test.

Alert level C

```
PLAT241_ALERT_2_C High   'MainMol' Ueq as Compared to Neighbors of        C38 Check
PLAT242_ALERT_2_C Low    'MainMol' Ueq as Compared to Neighbors of        C37 Check
PLAT313_ALERT_2_C Oxygen with Three Covalent Bonds (rare) ........         O3 Check
PLAT313_ALERT_2_C Oxygen with Three Covalent Bonds (rare) ........         O4 Check
PLAT313_ALERT_2_C Oxygen with Three Covalent Bonds (rare) ........         O2 Check
PLAT340_ALERT_3_C Low Bond Precision on  C-C Bonds ...............    0.00445 Ang.
PLAT910_ALERT_3_C Missing # of FCF Reflection(s) Below Theta(Min).         10 Note
PLAT911_ALERT_3_C Missing FCF Refl Between Thmin & STh/L=    0.600         80 Report
```

Alert level G

```
PLAT002_ALERT_2_G Number of Distance or Angle Restraints on AtSite         19 Note
PLAT003_ALERT_2_G Number of Uiso or Uij Restrained non-H Atoms ...         72 Report
PLAT154_ALERT_1_G The s.u.'s on the Cell Angles are Equal ..(Note)      0.004 Degree
PLAT176_ALERT_4_G The CIF-Embedded .res File Contains SADI Records          3 Report
PLAT187_ALERT_4_G The CIF-Embedded .res File Contains RIGU Records          1 Report
PLAT301_ALERT_3_G Main Residue  Disorder ..............(Resd  1  )        22% Note
PLAT301_ALERT_3_G Main Residue  Disorder ..............(Resd  2  )         3% Note
PLAT860_ALERT_3_G Number of Least-Squares Restraints .............        600 Note
PLAT912_ALERT_4_G Missing # of FCF Reflections Above STh/L=  0.600          6 Note
PLAT978_ALERT_2_G Number C-C Bonds with Positive Residual Density.          2 Info
```

0 **ALERT level A** = Most likely a serious problem - resolve or explain
0 **ALERT level B** = A potentially serious problem, consider carefully
8 **ALERT level C** = Check. Ensure it is not caused by an omission or oversight
10 **ALERT level G** = General information/check it is not something unexpected

1 ALERT type 1 CIF construction/syntax error, inconsistent or missing data
8 ALERT type 2 Indicator that the structure model may be wrong or deficient
6 ALERT type 3 Indicator that the structure quality may be low
3 ALERT type 4 Improvement, methodology, query or suggestion
0 ALERT type 5 Informative message, check

Datablock: 9-THF

Bond precision: C-C = 0.0045 A   Wavelength=0.71073

Cell:	a=9.9185(9)	b=10.8375(7)	c=11.0792(7)
	alpha=98.833(5)	beta=103.526(6)	gamma=109.936(7)
Temperature:	150 K		

	Calculated	Reported
Volume	1052.45(15)	1052.44(14)
Space group	P -1	P -1
Hall group	-P 1	-P 1
Moiety formula	C32 H76 N2 Na2 O2 Si4	C32 H76 N2 Na2 O2 Si4
Sum formula	C32 H76 N2 Na2 O2 Si4	C32 H76 N2 Na2 O2 Si4
Mr	679.29	679.28
Dx,g cm-3	1.072	1.072
Z	1	1
Mu (mm-1)	0.189	0.189
F000	376.0	376.0
F000’	376.47	
h,k,lmax	12,13,13	12,13,13
Nref	4334	4318
Tmin,Tmax	0.912,0.973	0.316,1.000
Tmin’	0.903	

Correction method= # Reported T Limits: Tmin=0.316 Tmax=1.000
AbsCorr = GAUSSIAN

Data completeness= 0.996 Theta(max)= 26.420

R(reflections)= 0.0594(3108) wR2(reflections)= 0.1561(4318)

S = 1.029 Npar= 250

The following ALERTS were generated. Each ALERT has the format
test-name_ALERT_alert-type_alert-level.
Click on the hyperlinks for more details of the test.

Alert level C

```
PLAT340_ALERT_3_C Low Bond Precision on  C-C Bonds ...............     0.0045 Ang.
PLAT921_ALERT_1_C R1  in the CIF and FCF Differ by ...............     0.0020 Check
PLAT922_ALERT_1_C wR2 in the CIF and FCF Differ by ...............     0.0042 Check
PLAT923_ALERT_1_C S    Values in the CIF and FCF Differ by .......      0.029 Check
```

Alert level G

```
PLAT002_ALERT_2_G Number of Distance or Angle Restraints on AtSite         17 Note
PLAT003_ALERT_2_G Number of Uiso or Uij Restrained non-H Atoms ...         26 Report
PLAT176_ALERT_4_G The CIF-Embedded .res File Contains SADI Records          3 Report
PLAT187_ALERT_4_G The CIF-Embedded .res File Contains RIGU Records          1 Report
PLAT301_ALERT_3_G Main Residue  Disorder ..............(Resd  1  )        24% Note
PLAT720_ALERT_4_G Number of Unusual/Non-Standard Labels ..........         12 Note
PLAT860_ALERT_3_G Number of Least-Squares Restraints .............        235 Note
PLAT910_ALERT_3_G Missing # of FCF Reflection(s) Below Theta(Min).          2 Note
PLAT912_ALERT_4_G Missing # of FCF Reflections Above STh/L=  0.600         13 Note
PLAT978_ALERT_2_G Number C-C Bonds with Positive Residual Density.          1 Info
```

---

0 **ALERT level A** = Most likely a serious problem - resolve or explain
0 **ALERT level B** = A potentially serious problem, consider carefully

```
  4 ALERT level C = Check. Ensure it is not caused by an omission or oversight
 10 ALERT level G = General information/check it is not something unexpected

  3 ALERT type 1 CIF construction/syntax error, inconsistent or missing data
  3 ALERT type 2 Indicator that the structure model may be wrong or deficient
  4 ALERT type 3 Indicator that the structure quality may be low
  4 ALERT type 4 Improvement, methodology, query or suggestion
  0 ALERT type 5 Informative message, check
```

Datablock: 10

```
Bond precision:  C-C = 0.0024 A          Wavelength=1.54184

Cell:            a=18.2177(3)    b=9.00185(10)       c=17.2035(2)
                 alpha=90        beta=95.3841(12)    gamma=90
Temperature:     150 K

                 Calculated                  Reported
Volume           2808.81(6)                  2808.80(6)
Space group      P 21/c                      P 1 21/c 1
Hall group       -P 2ybc                     -P 2ybc
Moiety formula   C18 H42 N Na Si2, C7 H8     C25 H50 N Na Si2
Sum formula      C25 H50 N Na Si2            C25 H50 N Na Si2
Mr               443.83                      443.83
Dx,g cm-3        1.050                       1.050
Z                4                           4
Mu (mm-1)        1.358                       1.358
F000             984.0                       984.0
F000'            988.36
h,k,lmax         21,10,20                    21,10,20
Nref             5147                        5057
Tmin,Tmax        0.850,0.873                 0.514,1.000
Tmin'            0.762

Correction method= # Reported T Limits: Tmin=0.514 Tmax=1.000
AbsCorr = MULTI-SCAN

Data completeness= 0.983          Theta(max)= 68.221

R(reflections)= 0.0363( 4482)     wR2(reflections)= 0.1030( 5057)

S = 1.044                   Npar= 275
```

The following ALERTS were generated. Each ALERT has the format
test-name_ALERT_alert-type_alert-level.
Click on the hyperlinks for more details of the test.

Alert level C

```
PLAT241_ALERT_2_C High   'MainMol' Ueq as Compared to Neighbors of        Na1 Check
PLAT911_ALERT_3_C Missing FCF Refl Between Thmin & STh/L=    0.600         73 Report
```

Alert level G

```
PLAT042_ALERT_1_G Calc. and Reported MoietyFormula Strings  Differ     Please Check
PLAT764_ALERT_4_G Overcomplete CIF Bond List Detected (Rep/Expd) .       1.20 Ratio
PLAT910_ALERT_3_G Missing # of FCF Reflection(s) Below Theta(Min).          2 Note
PLAT912_ALERT_4_G Missing # of FCF Reflections Above STh/L=  0.600         15 Note
PLAT978_ALERT_2_G Number C-C Bonds with Positive Residual Density.         10 Info
```

---

0 **ALERT level A** = Most likely a serious problem - resolve or explain
0 **ALERT level B** = A potentially serious problem, consider carefully
2 **ALERT level C** = Check. Ensure it is not caused by an omission or oversight
5 **ALERT level G** = General information/check it is not something unexpected

1 ALERT type 1 CIF construction/syntax error, inconsistent or missing data
2 ALERT type 2 Indicator that the structure model may be wrong or deficient
2 ALERT type 3 Indicator that the structure quality may be low
2 ALERT type 4 Improvement, methodology, query or suggestion
0 ALERT type 5 Informative message, check

It is advisable to attempt to resolve as many as possible of the alerts in all categories. Often the minor alerts point to easily fixed oversights, errors and omissions in your CIF or refinement strategy, so attention to these fine details can be worthwhile. In order to resolve some of the more serious problems it may be necessary to carry out additional measurements or structure refinements. However, the purpose of your study may justify the reported deviations and the more serious of these should normally be commented upon in the discussion or experimental section of a paper or in the "special_details" fields of the CIF. checkCIF was carefully designed to identify outliers and unusual parameters, but every test has its limitations and alerts that are not important in a particular case may appear. Conversely, the absence of alerts does not guarantee there are no aspects of the results needing attention. It is up to the individual to critically assess their own results and, if necessary, seek expert advice.

Publication of your CIF in IUCr journals

A basic structural check has been run on your CIF. These basic checks will be run on all CIFs submitted for publication in IUCr journals (*Acta Crystallographica*, *Journal of Applied Crystallography*, *Journal of Synchrotron Radiation*); however, if you intend to submit to *Acta Crystallographica Section C* or *E* or *IUCrData*, you should make sure that full publication checks are run on the final version of your CIF prior to submission.

Publication of your CIF in other journals

Please refer to the *Notes for Authors* of the relevant journal for any special instructions relating to CIF submission.

PLATON version of 30/01/2018; check.def file version of 30/01/2018

Datablock 1 - ellipsoid plot

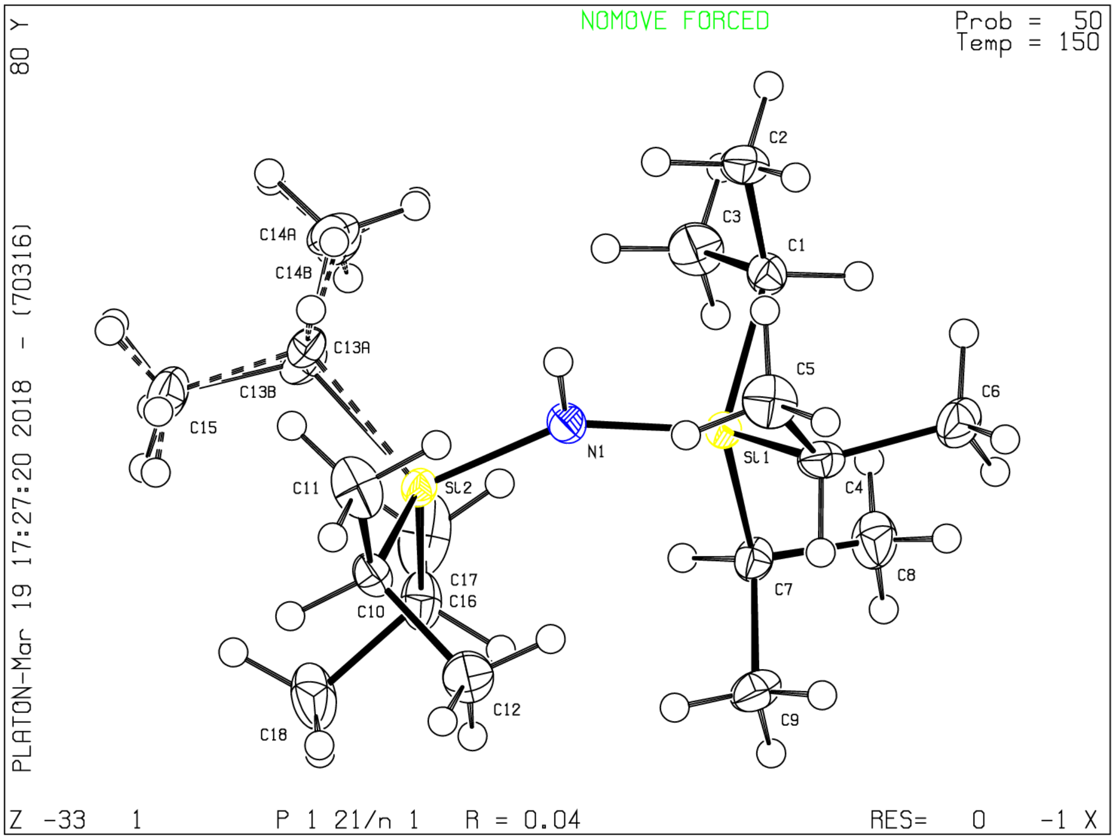

Datablock 2 - ellipsoid plot

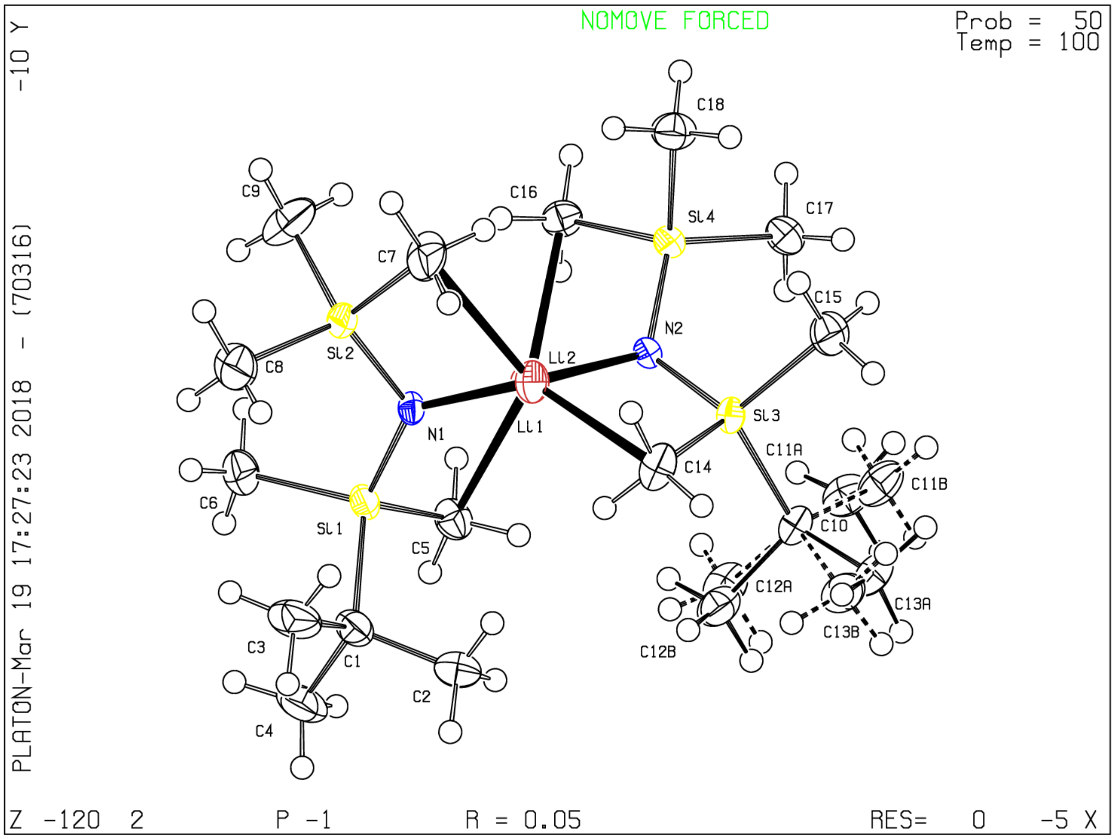

Datablock 3 - ellipsoid plot

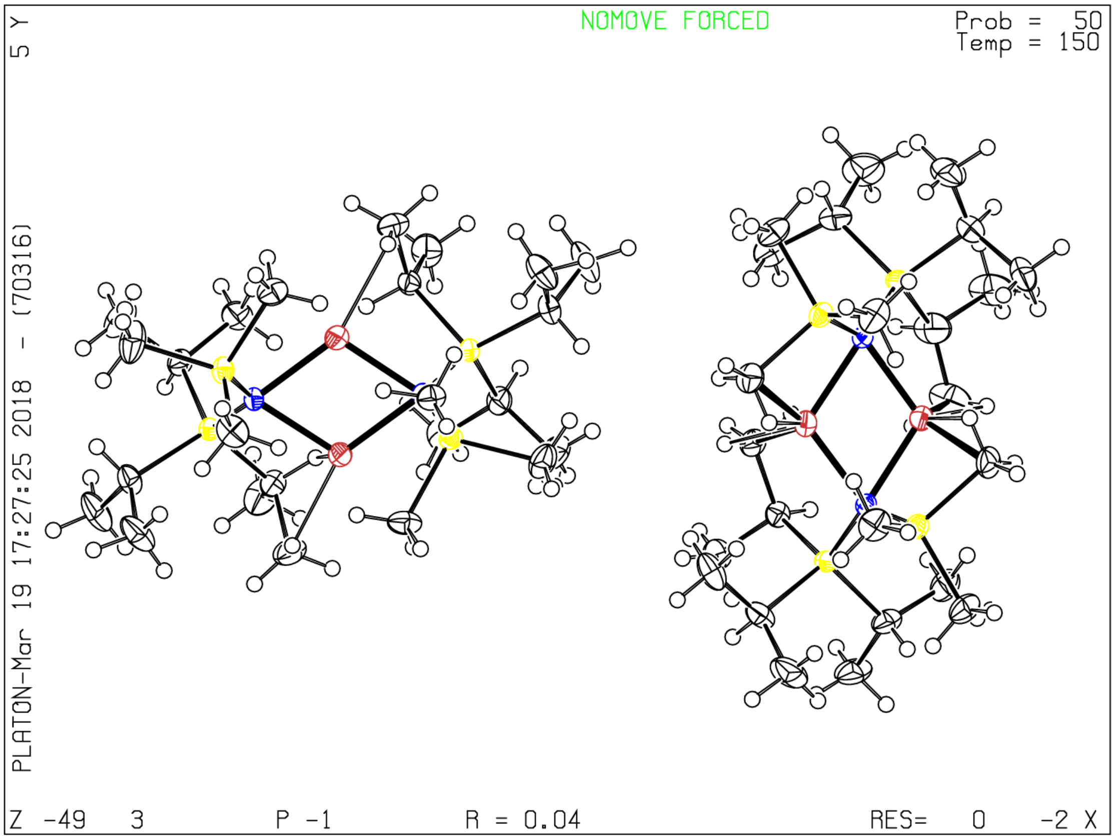

Datablock 4 - ellipsoid plot

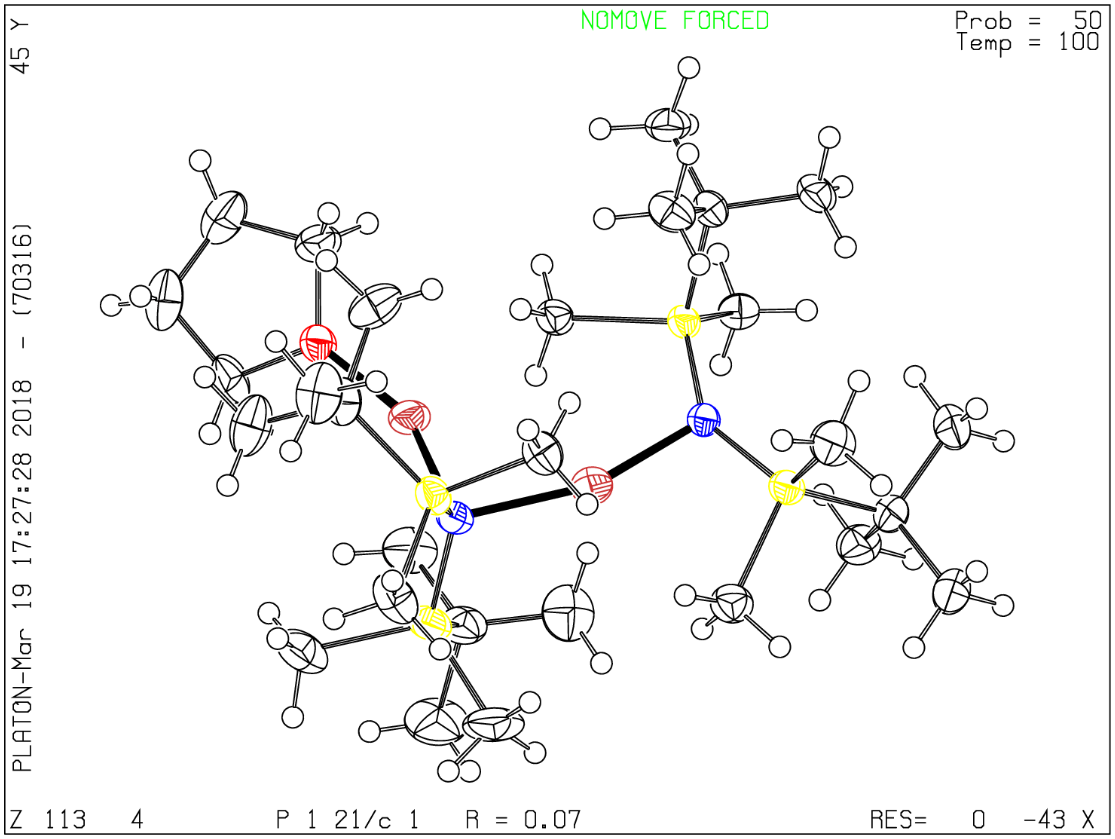

Datablock 5 - ellipsoid plot

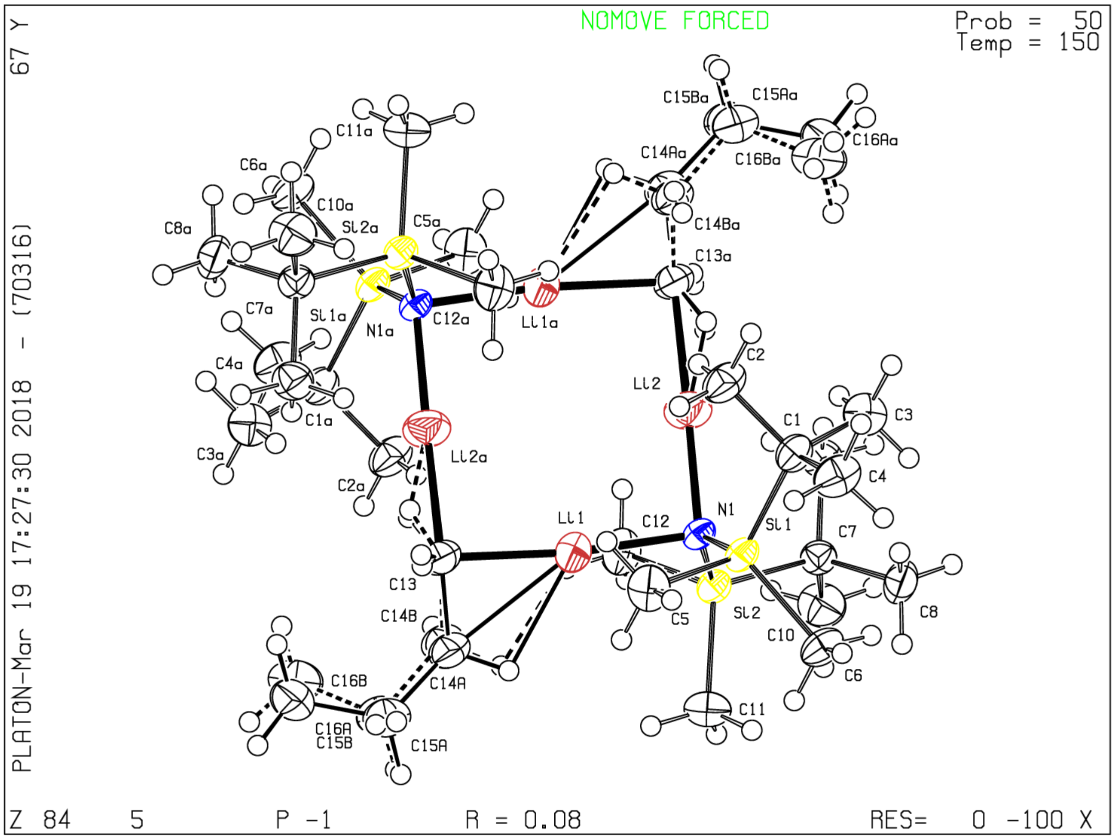

Datablock 6 - ellipsoid plot

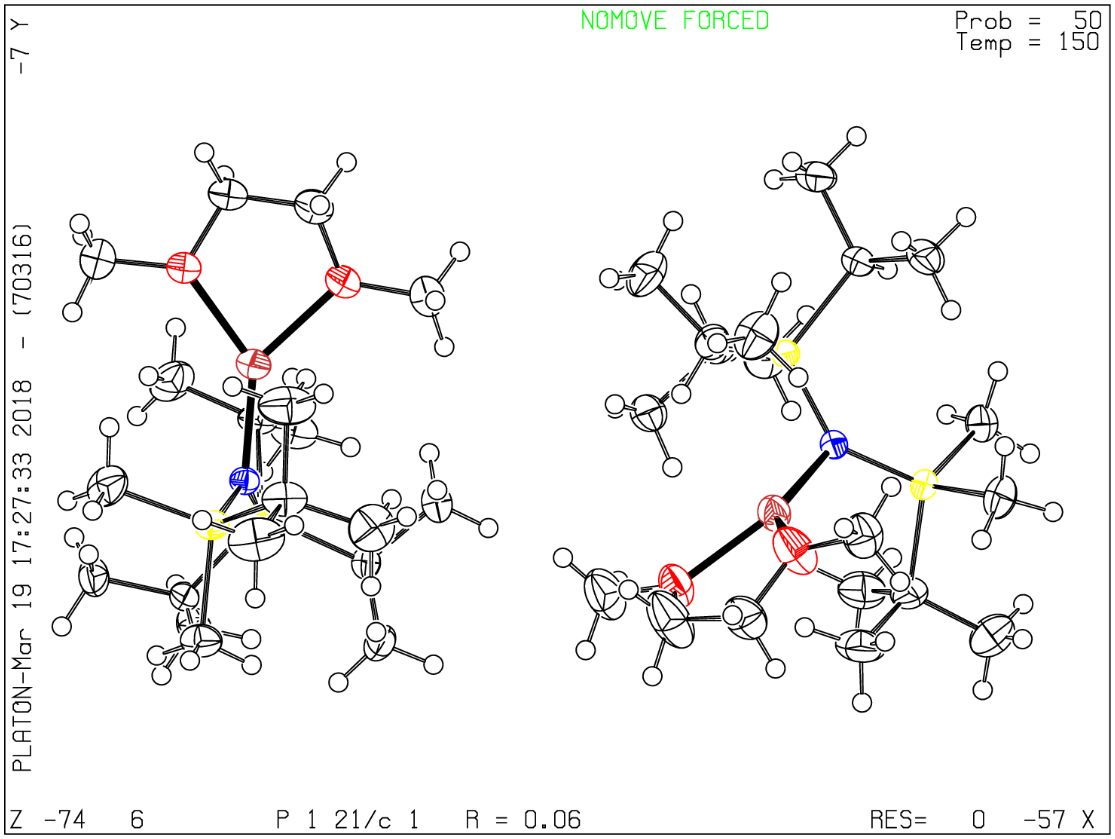

Datablock 7 - ellipsoid plot

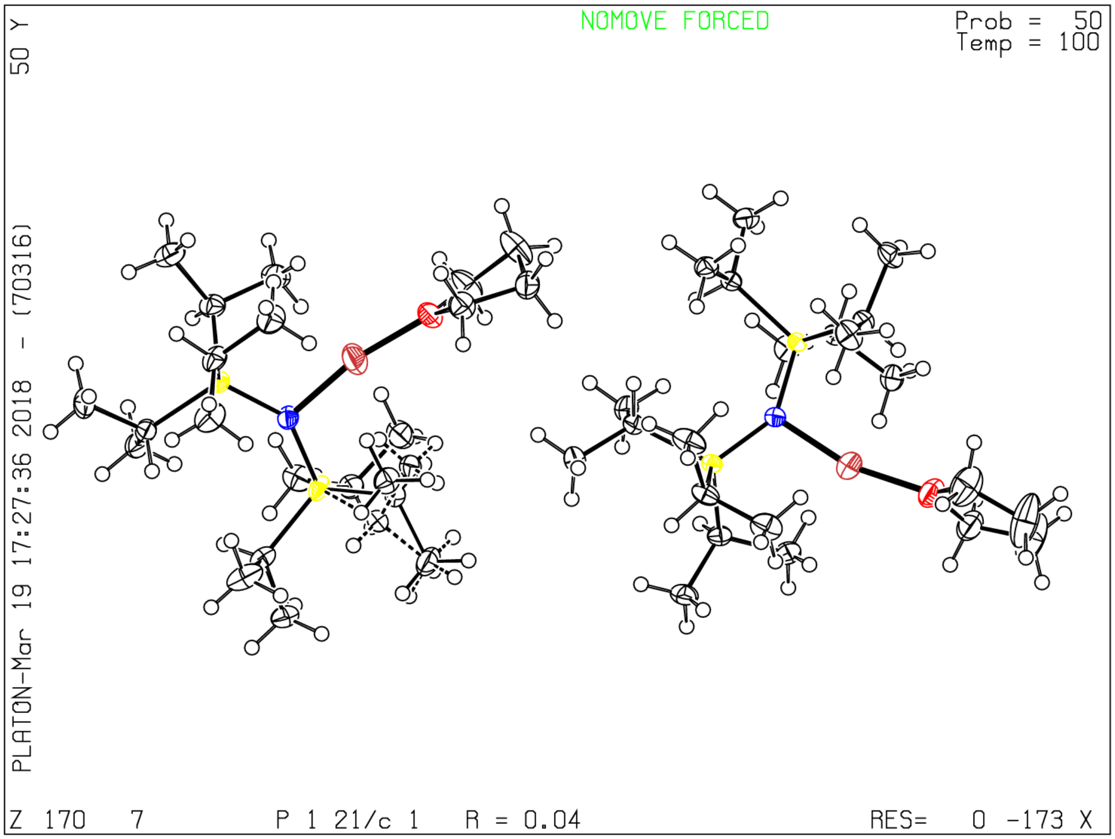

Datablock 8 - ellipsoid plot

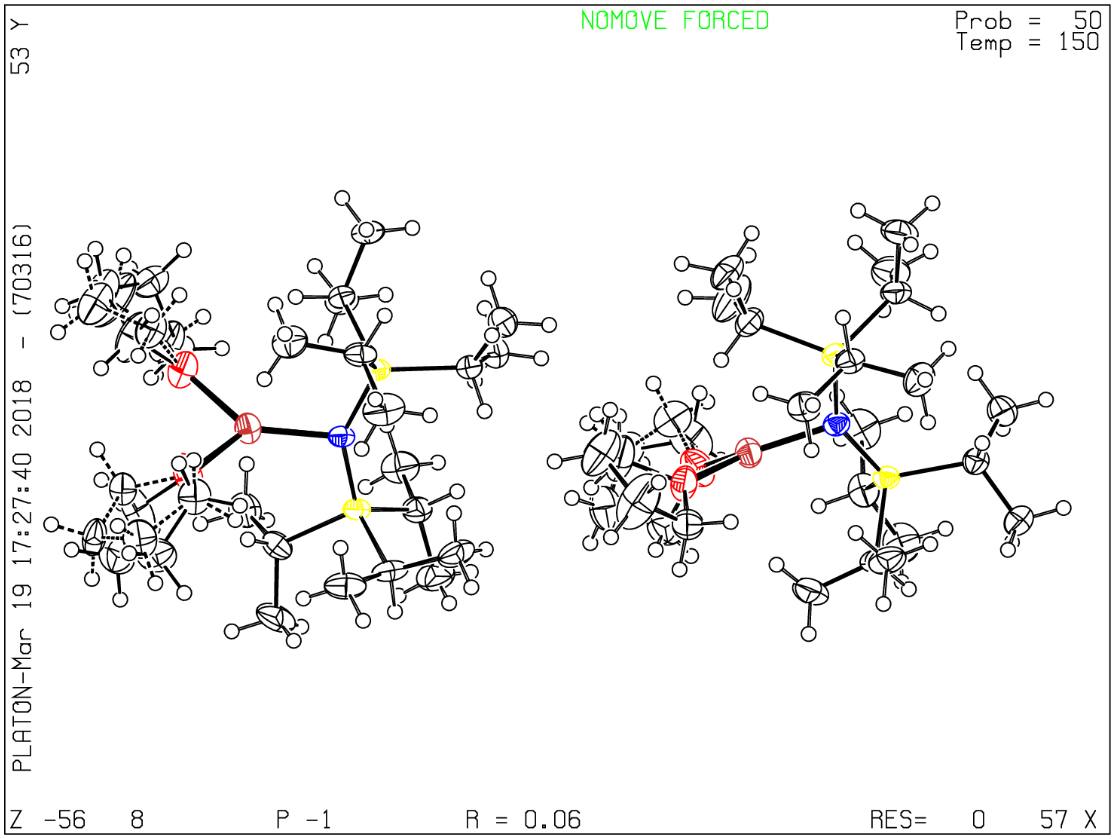

Datablock 9-THF - ellipsoid plot

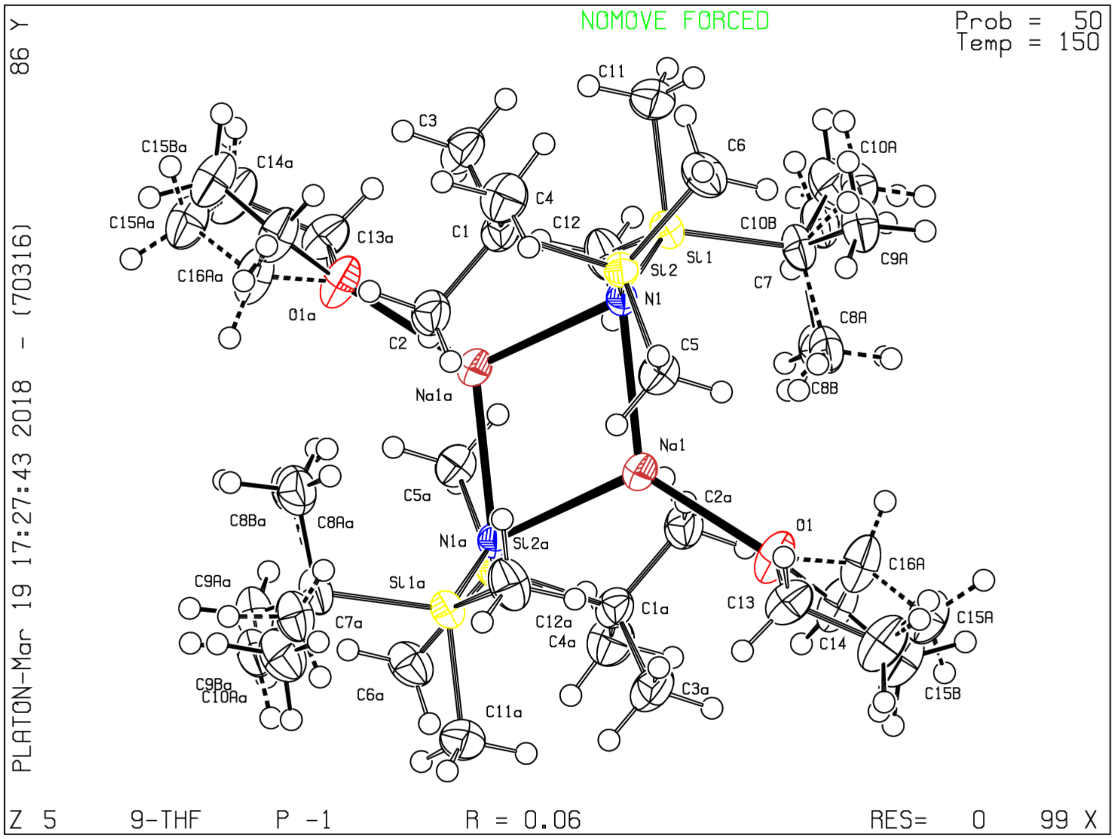

Datablock 10 - ellipsoid plot

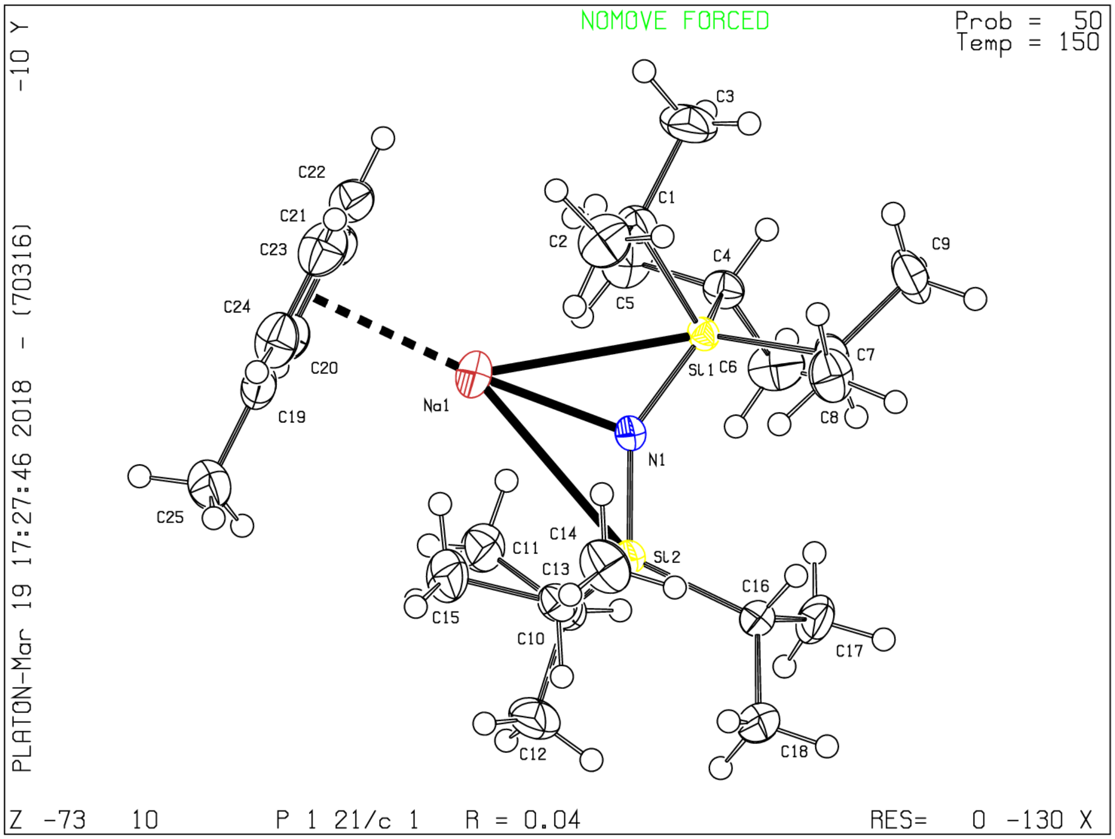

Supplement: Supplementary file 1 [file molecules-23-01138-s001.zip › BIGCIF_checkcif.pdf]
