# Supplementary material for: Structural Characterization of Lithium and Sodium Bulky Bis(silyl)amide Complexes
Source: Molecules. 2018 May 10;23(5):1138. doi: 10.3390/molecules23051138 (PMC6100173; doi:10.3390/molecules23051138)
Supplement: Supplementary file 1 [file molecules-23-01138-s001.zip › Mills_Mol_SupportingMaterials_submission.pdf]

Supplementary materials for

Structural Characterization of Lithium and Sodium Bulky Bis(silyl)amide Complexes

Hannah M. Nicholas[1], Conrad A. P. Goodwin[1], Jon G. C. Kragskow[1], Selena J. Lockyer[1], and David P. Mills[1]*

[1] School of Chemistry, The University of Manchester, Oxford Road, Manchester, M13 9PL, U.K.; hannah.nicholas@manchester.ac.uk; conrad.goodwin@manchester.ac.uk; jon.kragskow@student.manchester.ac.uk; selena.lockyer@postgrad.manchester.ac.uk

* Correspondence: david.mills@manchester.ac.uk; Tel.: +44-161-275-4606

Contents

1. **NMR spectra** **S2**
2. **FTIR spectra** **S13**
3. **X-ray crystallography** **S16**
4. **References** **S22**

1. NMR spectra

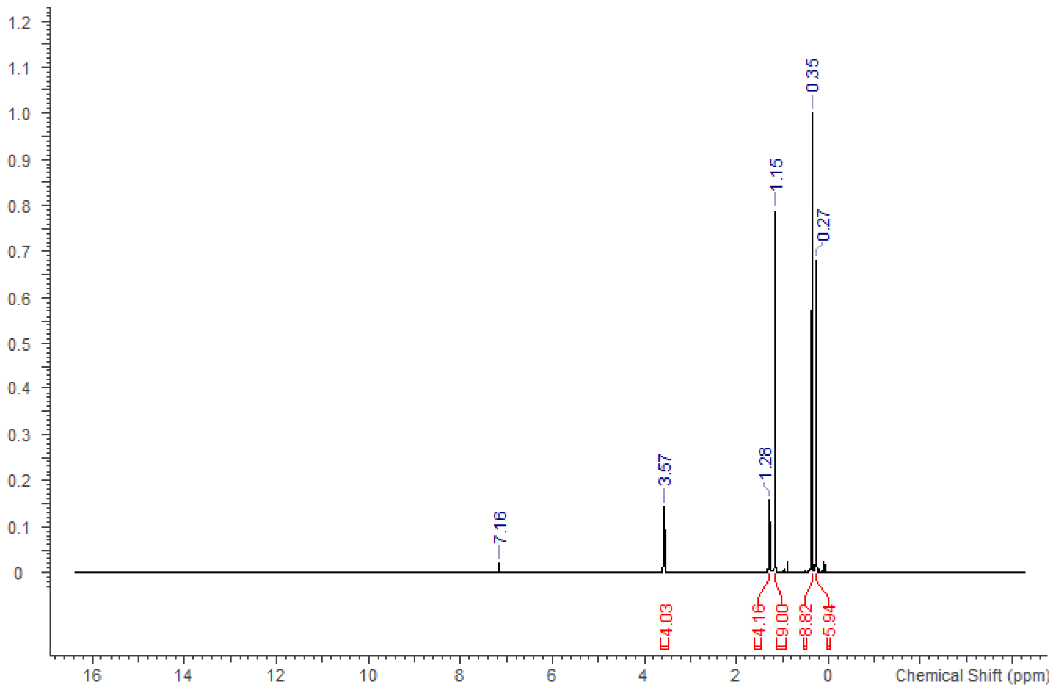

Figure S1. ^{1}H NMR spectrum of [Li{μ-N(Si^{t}BuMe$_2$)(SiMe$_3$)}(THF)]$_n$ (**2·THF**) in C_6D_6.

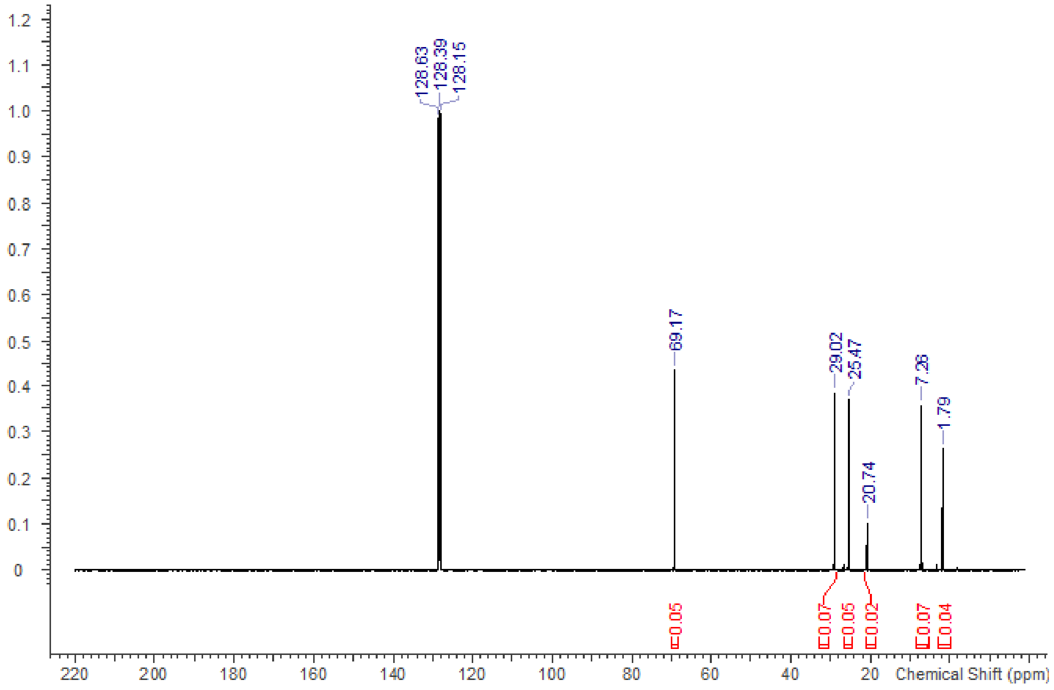

Figure S2. ^{13}C{^{1}H} NMR spectrum of [Li{μ-N(Si^{t}BuMe$_2$)(SiMe$_3$)}(THF)]$_n$ (**2·THF**) in C_6D_6.

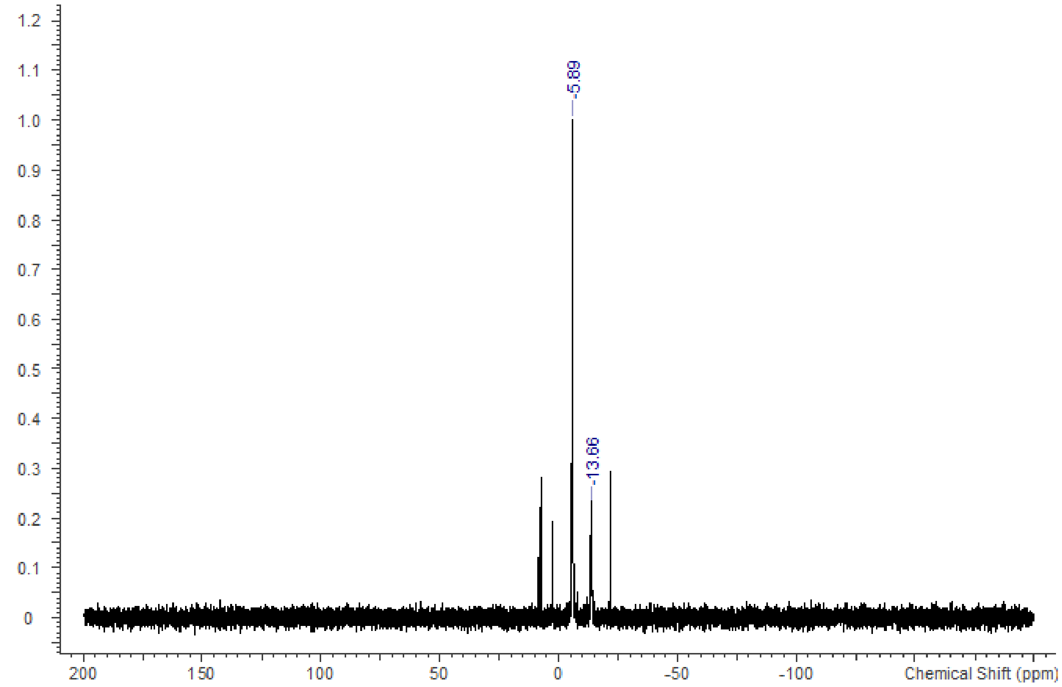

Figure S3. ^{29}Si{^{1}H} NMR spectrum of [Li{μ-N(Si^{t}BuMe$_2$)(SiMe$_3$)}(THF)]$_n$ (**2·THF**) in C_6D_6.

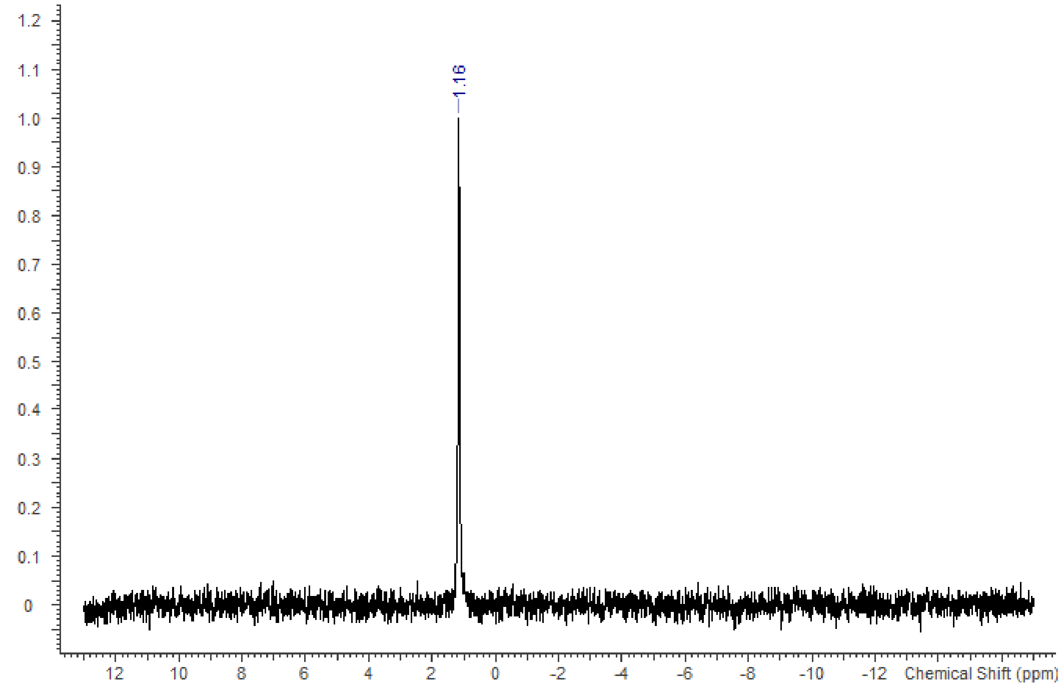

Figure S4. ^{7}Li{^{1}H} NMR spectrum of [Li{μ-N(Si^{t}BuMe$_2$)(SiMe$_3$)}(THF)]$_n$ (**2·THF**) in C_6D_6.

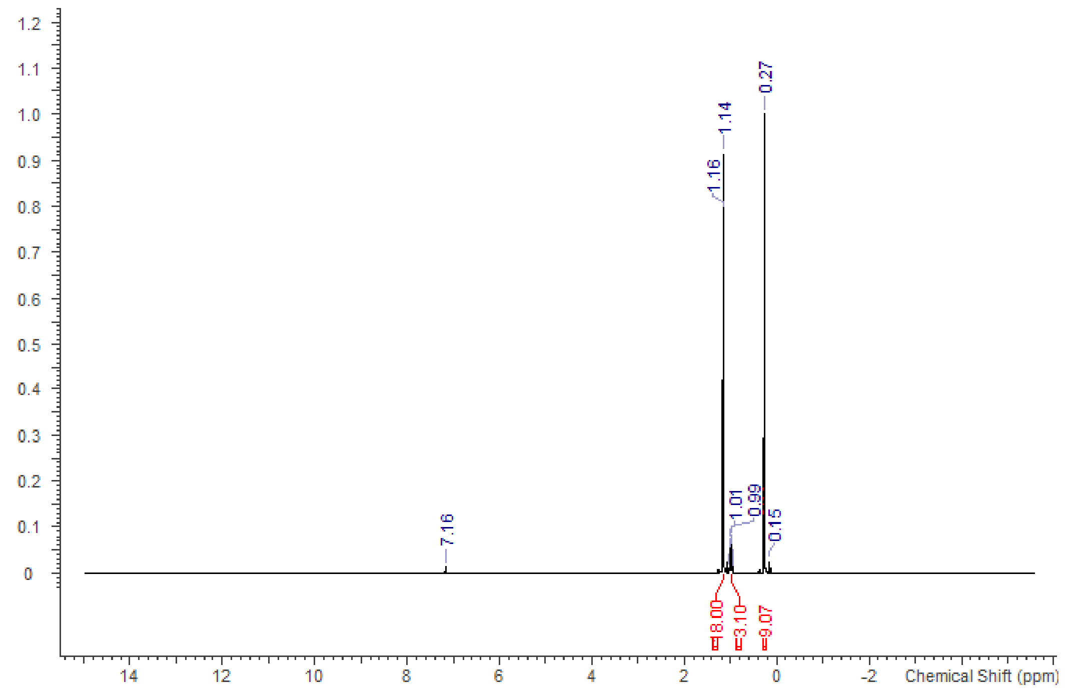

Figure S5. ^{1}H NMR spectrum of [Li{μ-N(Si^{i}Pr$_3$)(SiMe$_3$)}]$_2$ (**3**) in C_6D_6.

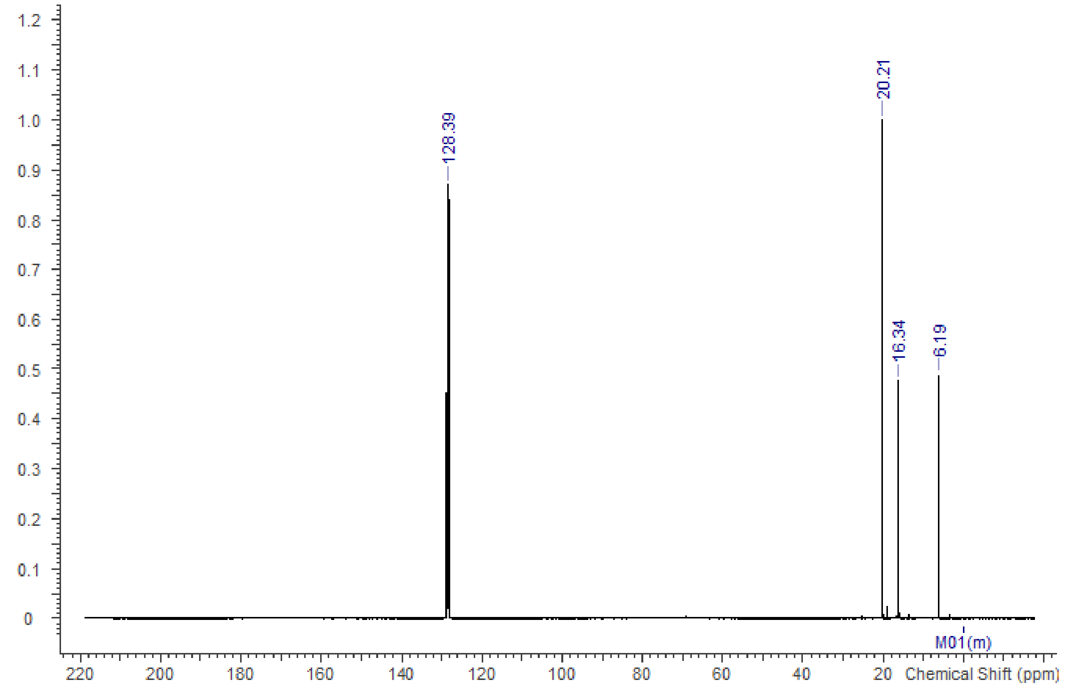

Figure S6. ^{13}C{^{1}H} NMR spectrum of [Li{μ-N(Si^{i}Pr$_3$)(SiMe$_3$)}]$_2$ (**3**) in C_6D_6.

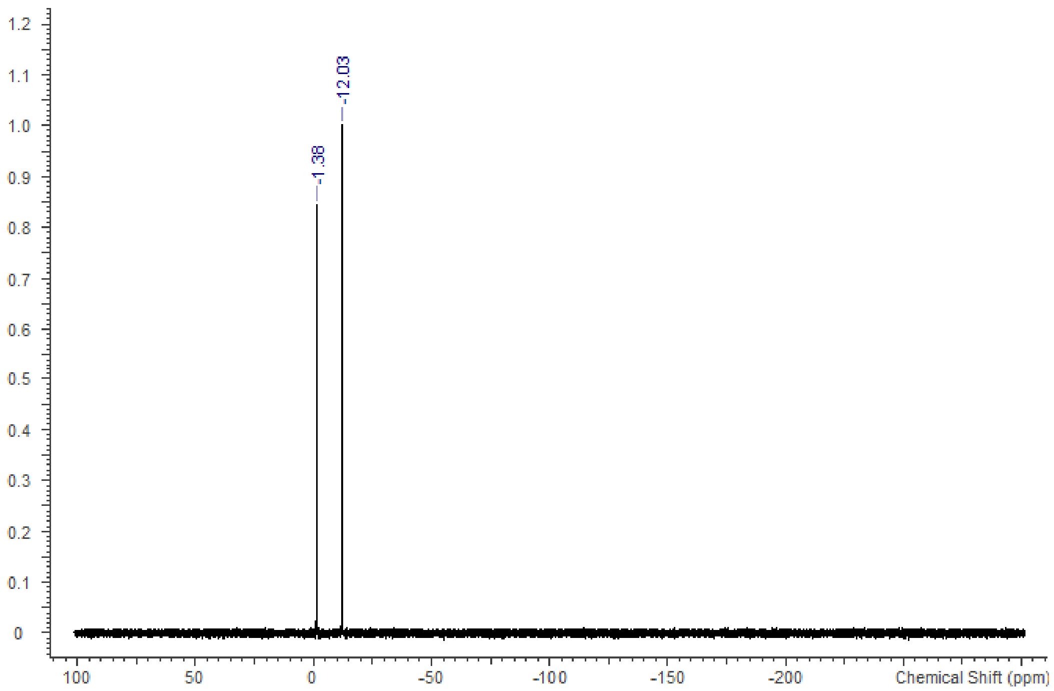

Figure S7. $^{29}Si\{^{1}H\}$ NMR spectrum of $[Li\{\mu\text{-}N(Si^{i}Pr_3)(SiMe_3)\}]_2$ (**3**) in C_6D_6.

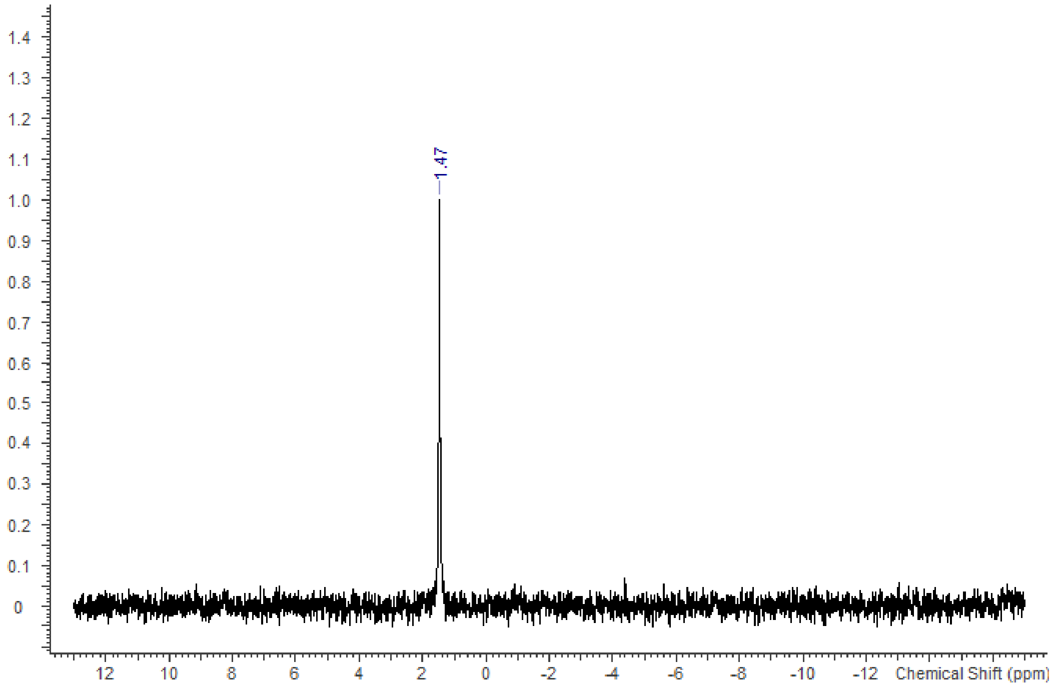

Figure S8. $^{7}Li\{^{1}H\}$ NMR spectrum of $[Li\{\mu\text{-}N(Si^{i}Pr_3)(SiMe_3)\}]_2$ (**3**) in C_6D_6.

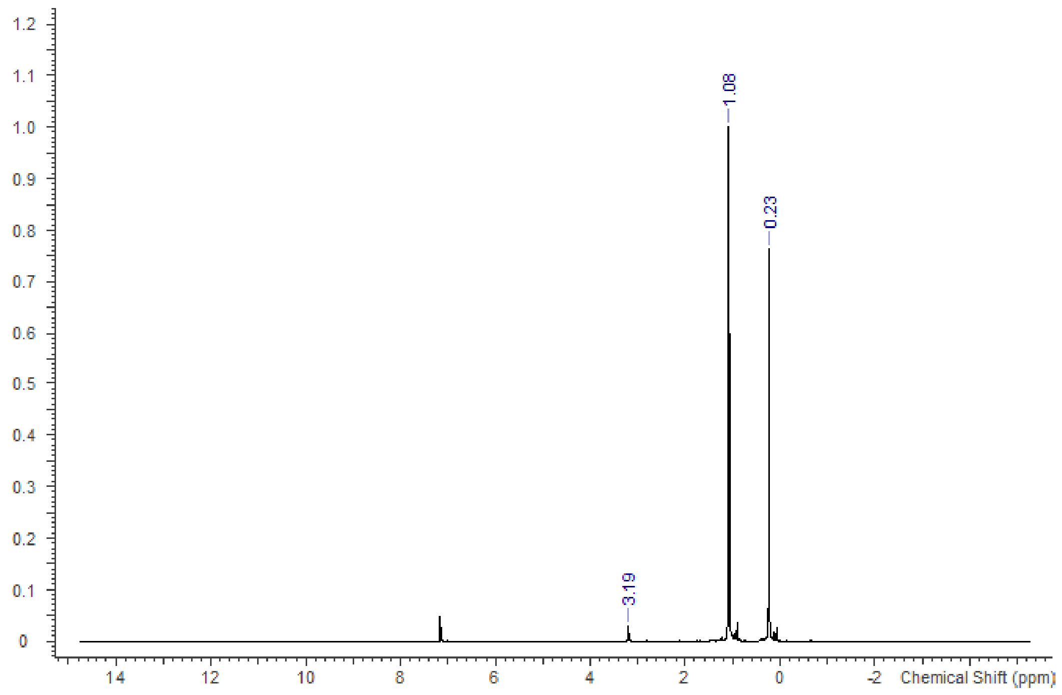

Figure S9. ^{1}H NMR spectrum of [Li{N(Si^{t}BuMe$_2$)$_2$}{μ-N(Si^{t}BuMe$_2$)$_2$}Li(THF)] (**4**) in C_6D_6.

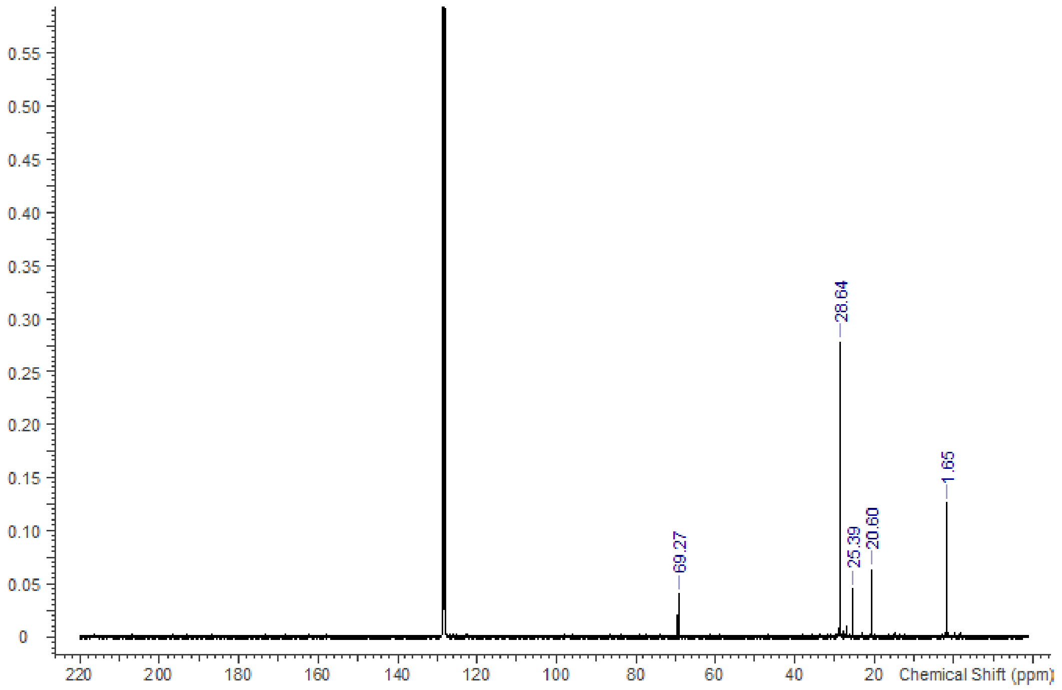

Figure S10. $^{13}C\{^{1}H\}$ NMR spectrum of [Li{N(Si^{t}BuMe$_2$)$_2$}{μ-N(Si^{t}BuMe$_2$)$_2$}Li(THF)] (**4**) in C_6D_6.

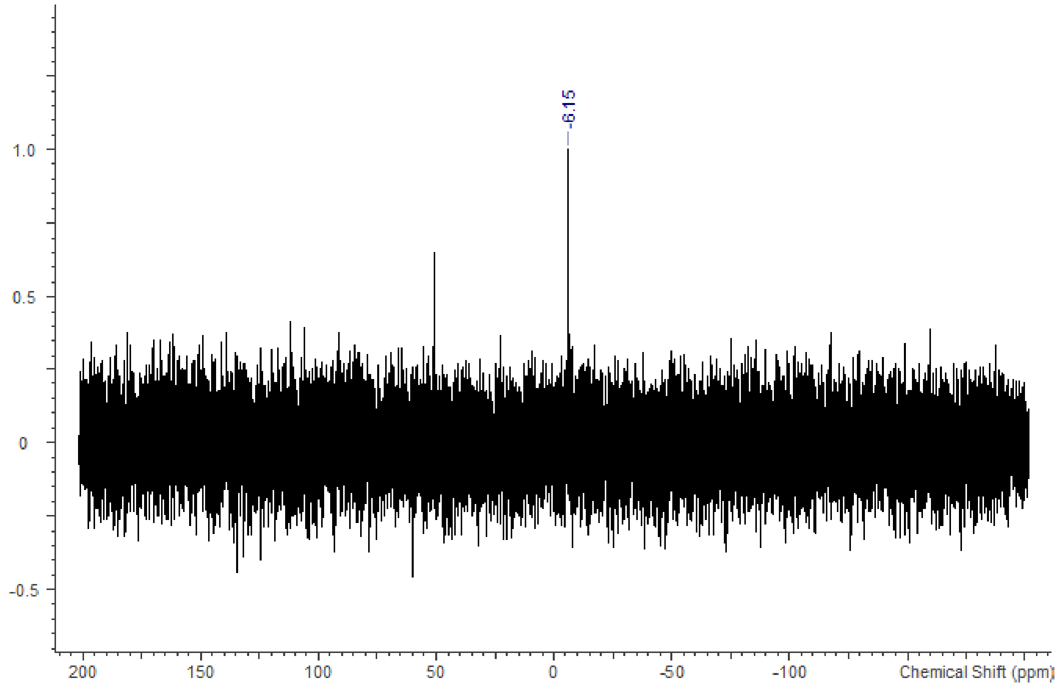

Figure S11. ^{29}Si{^{1}H} NMR spectrum of [Li{N(Si^{t}BuMe$_2$)$_2$}{μ-N(Si^{t}BuMe$_2$)$_2$}Li(THF)] (**4**) in C_6D_6.

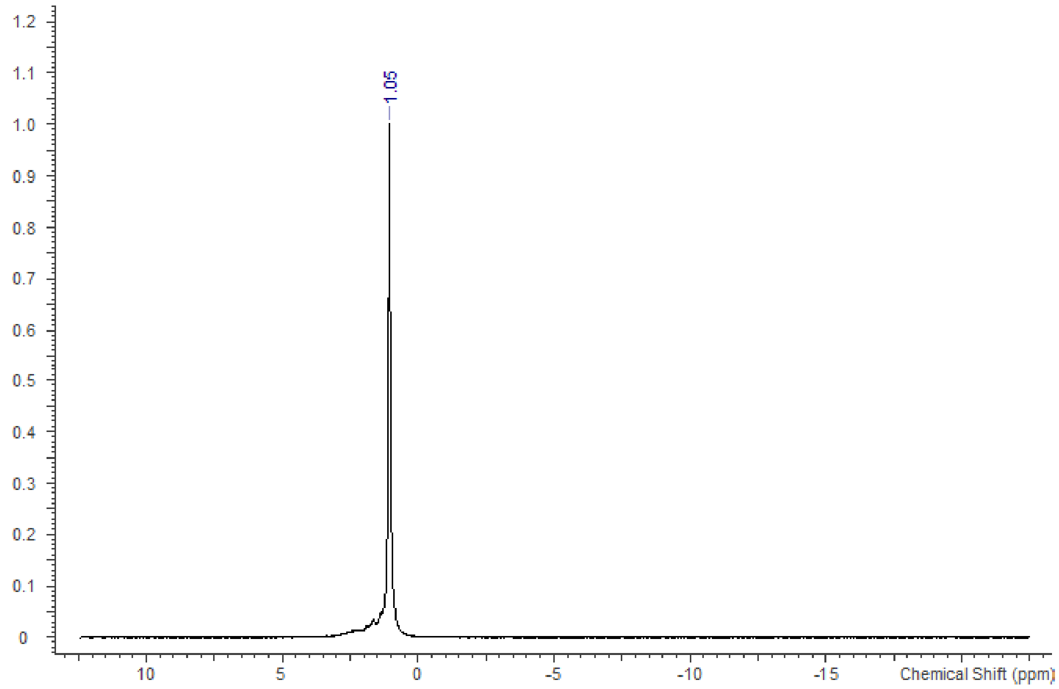

Figure S12. ^{7}Li{^{1}H} NMR spectrum of [Li{N(Si^{t}BuMe$_2$)$_2$}{μ-N(Si^{t}BuMe$_2$)$_2$}Li(THF)] (**4**) in C_6D_6.

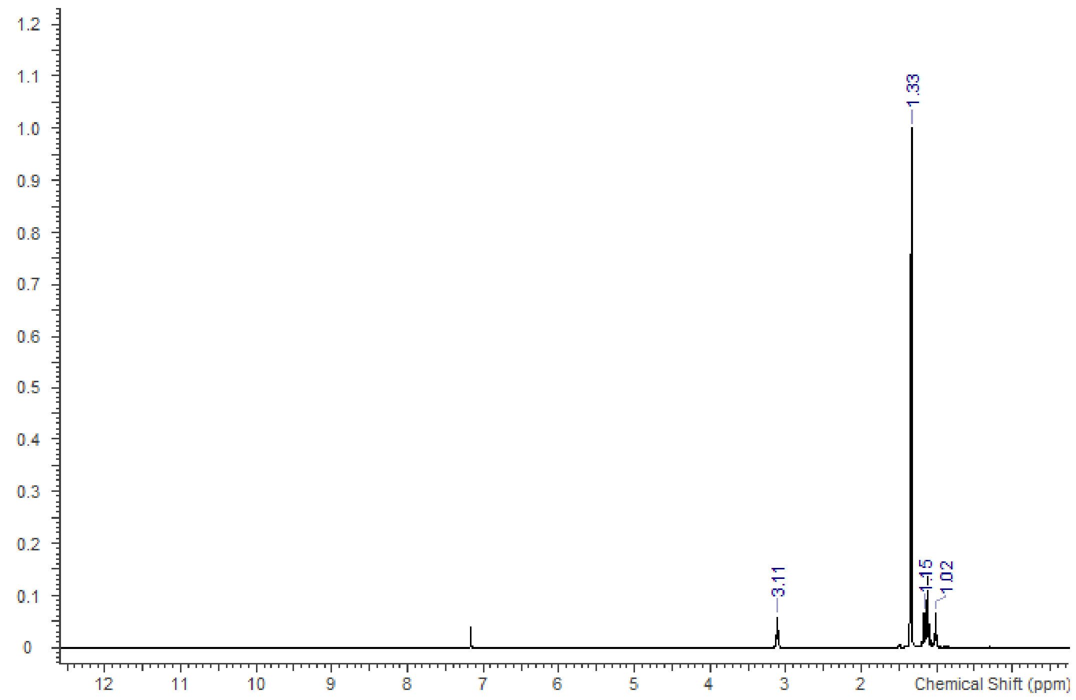

Figure S13. ^{1}H NMR spectrum of [Li{N(Si^{i}Pr$_3$)$_2$}(THF)] (**7**) in C_6D_6.

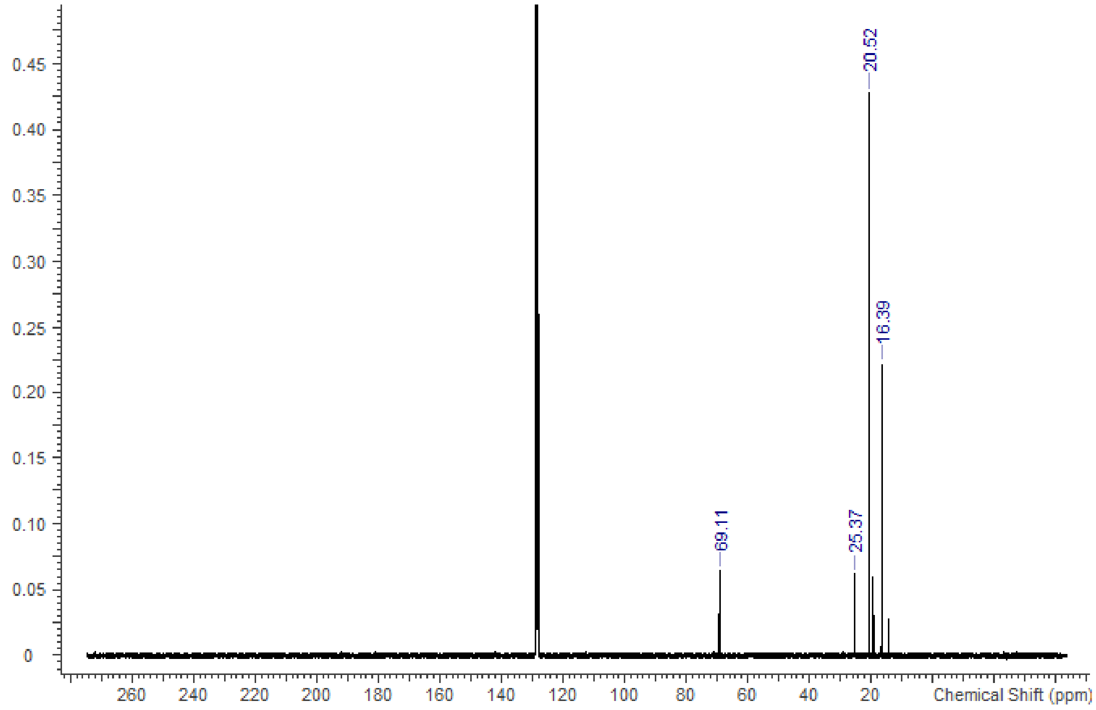

Figure S14. $^{13}C\{^{1}H\}$ NMR spectrum of [Li{N(Si^{i}Pr$_3$)$_2$}(THF)] (**7**) in C_6D_6.

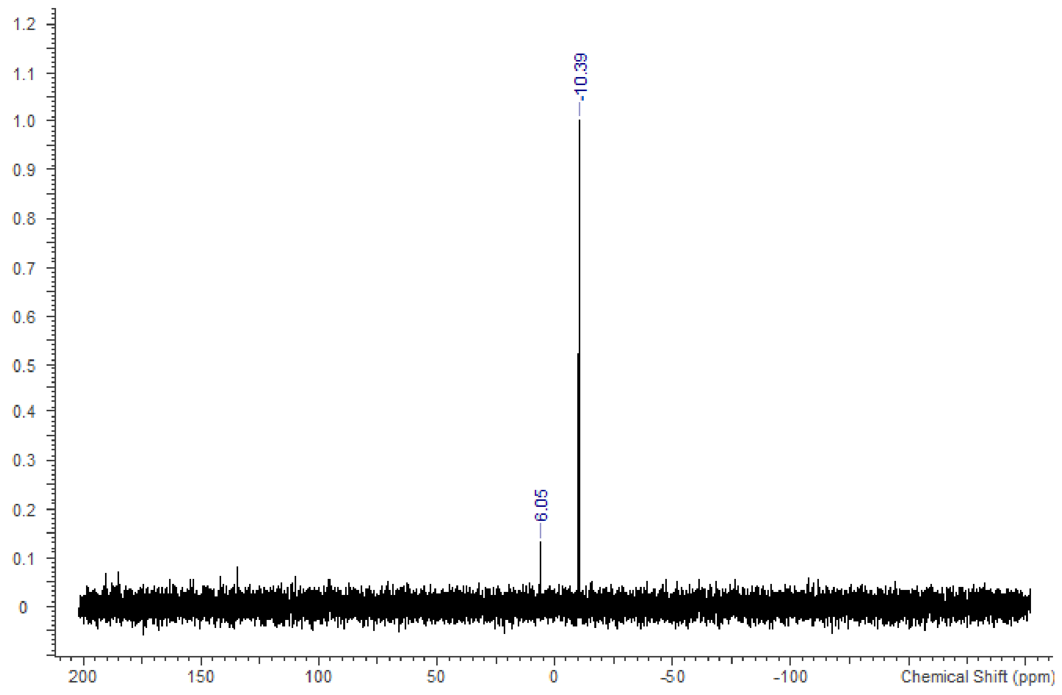

Figure S15. $^{29}Si\{^1H\}$ NMR spectrum of $[Li\{N(Si^iPr_3)_2\}(THF)]$ (**7**) in C_6D_6.

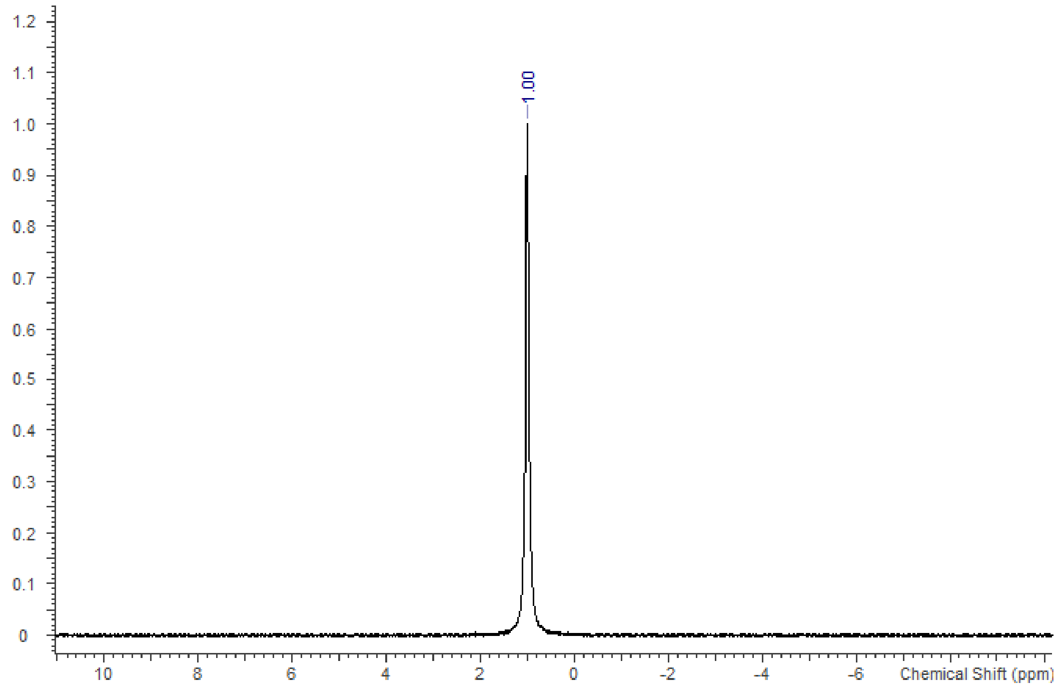

Figure S16. $^7Li\{^1H\}$ NMR spectrum of $[Li\{N(Si^iPr_3)_2\}(THF)]$ (**7**) in C_6D_6.

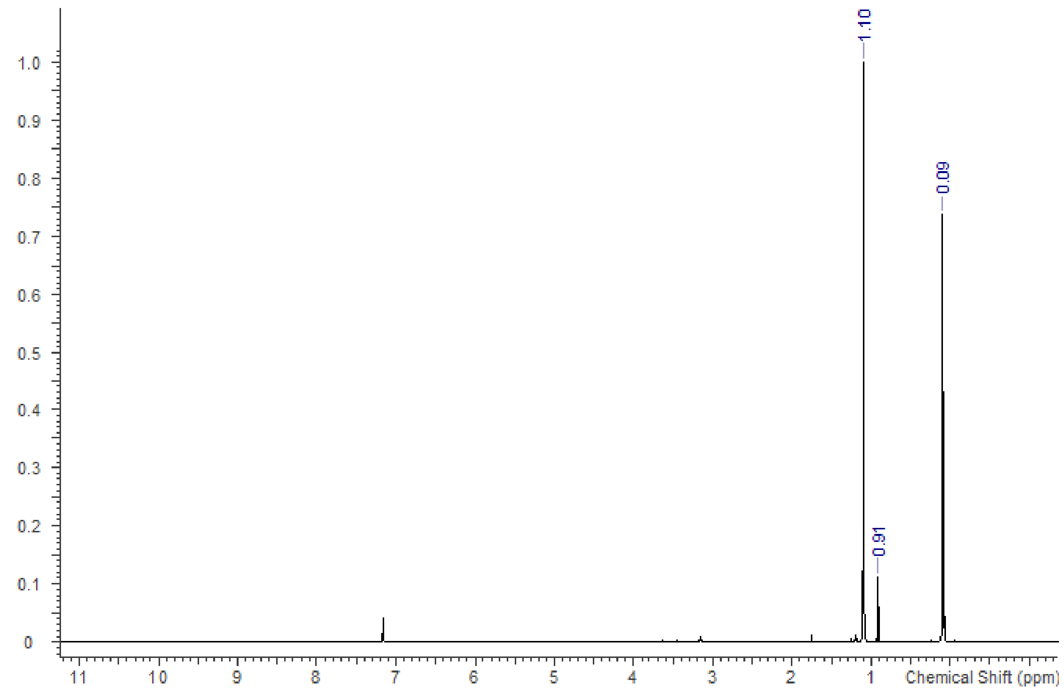

Figure S17. ^{1}H NMR spectrum of [Na{N(Si^{t}BuMe$_2$)$_2$}]$_n$ (**9**) in C_6D_6.

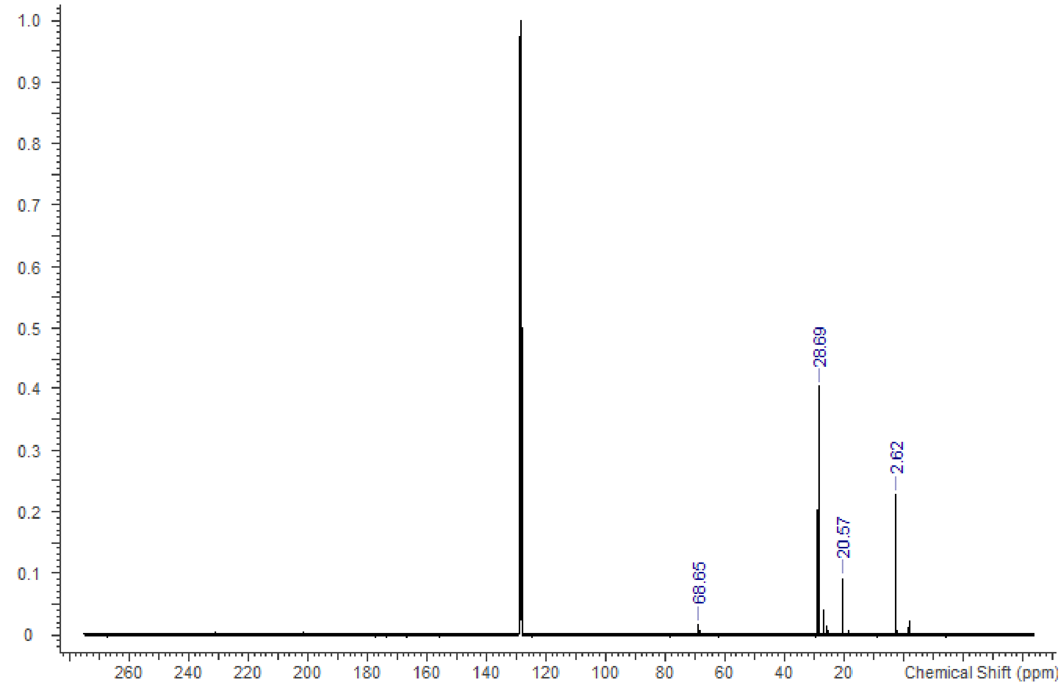

Figure S18. $^{13}C\{^1H\}$ NMR spectrum of [Na{N(Si^{t}BuMe$_2$)$_2$}]$_n$ (**9**) in C_6D_6.

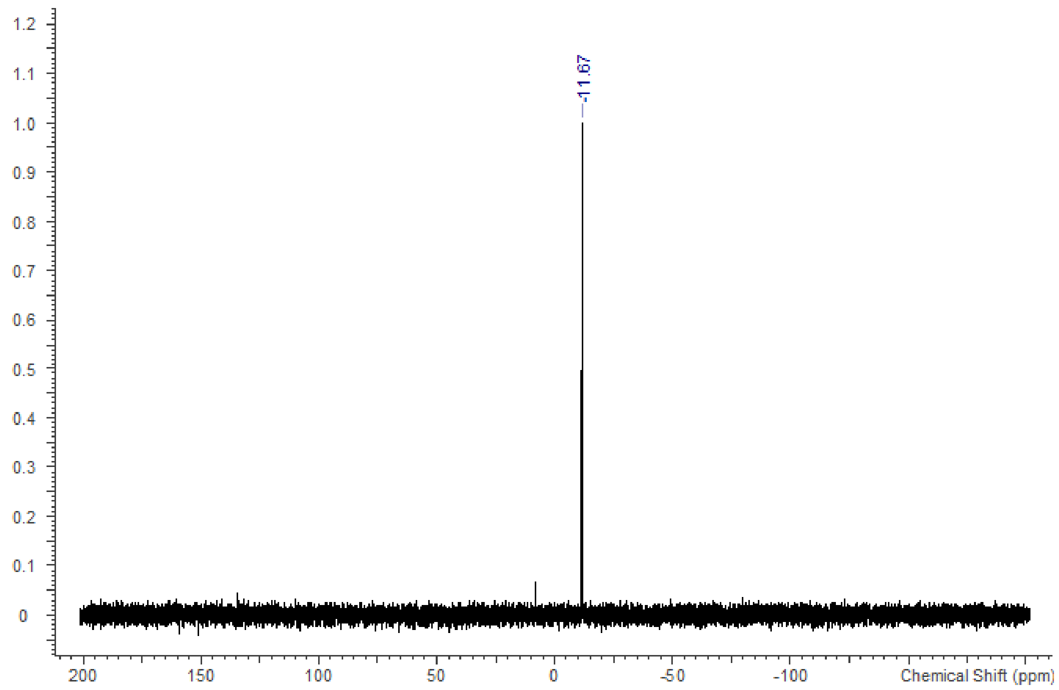

Figure S19. $^{29}Si\{^1H\}$ NMR spectrum of $[Na\{N(Si^tBuMe_2)_2\}]_n$ (**9**) in C_6D_6.

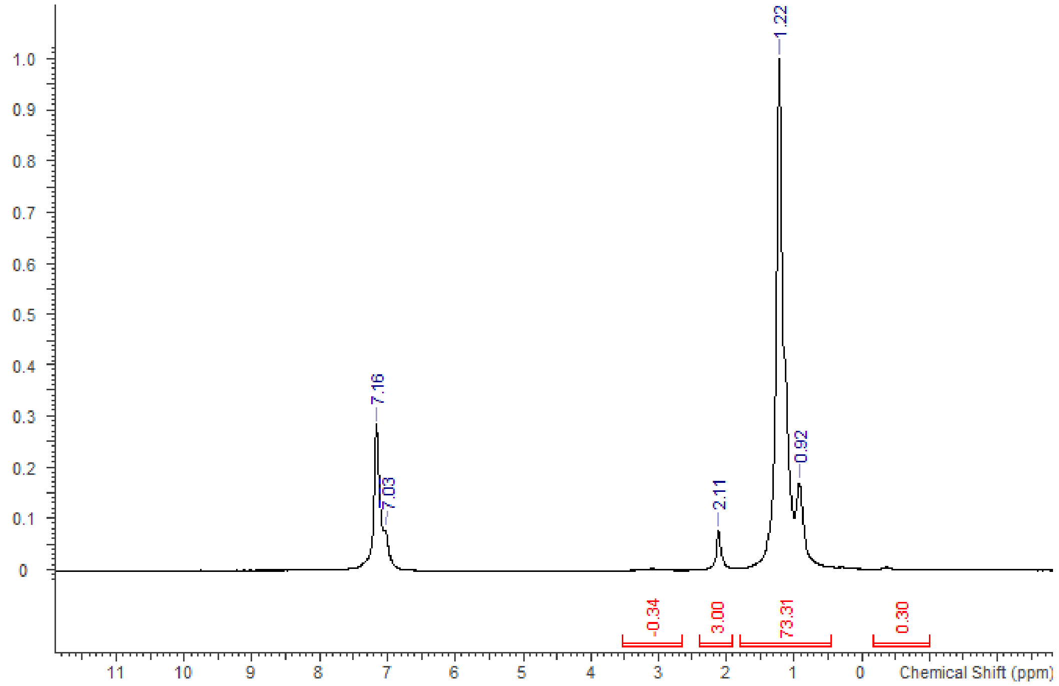

Figure S20. ^1H NMR spectrum of $[Na\{N(Si^iPr_3)_2\}(C_7H_8)]$ (**10**) in C_6D_6.

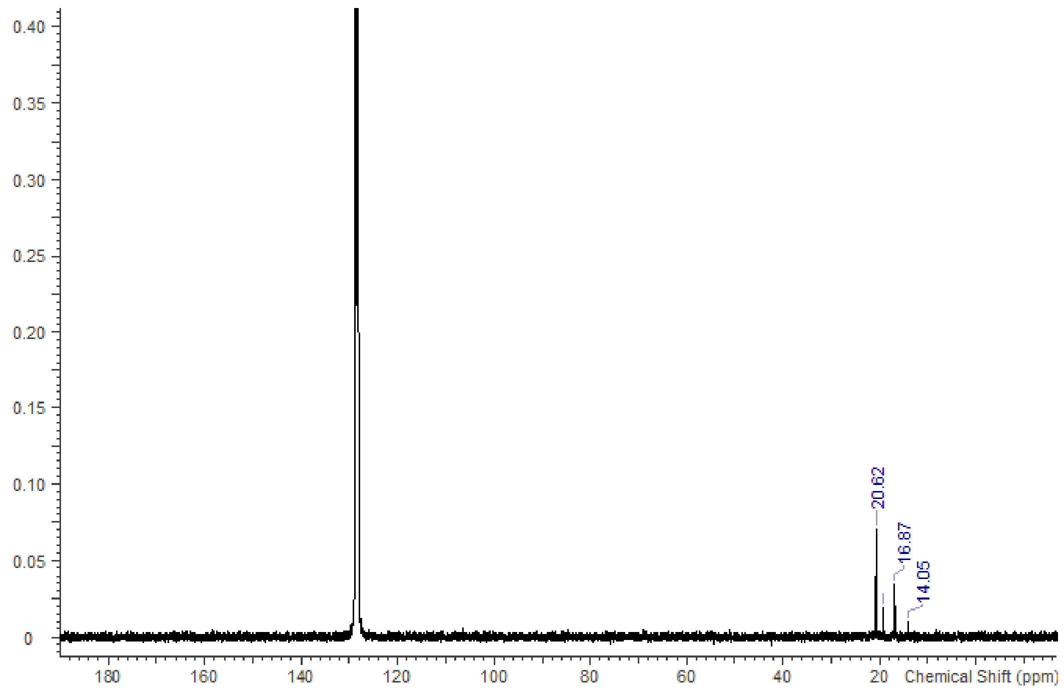

Figure S21. $^{13}C\{^{1}H\}$ NMR spectrum of [Na{N(Si^{i}Pr$_3$)$_2$}(C_7H_8)] (**10**) in C_6D_6.

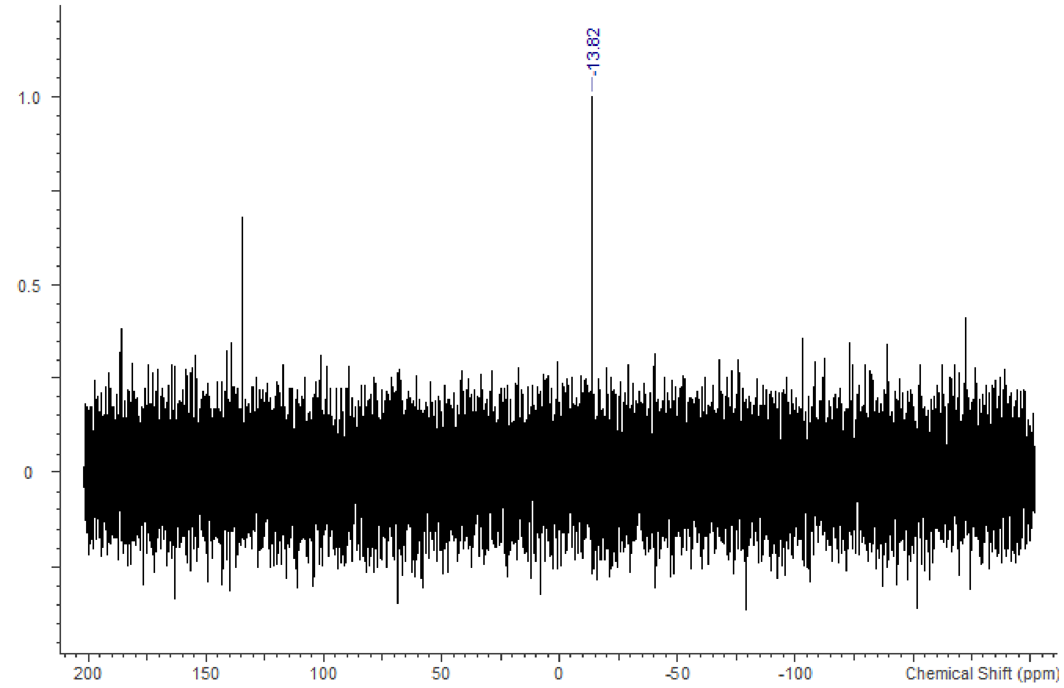

Figure S22. $^{29}Si\{^{1}H\}$ NMR spectrum of [Na{N(Si^{i}Pr$_3$)$_2$}(C_7H_8)] (**10**) in C_6D_6.

2. FTIR spectra

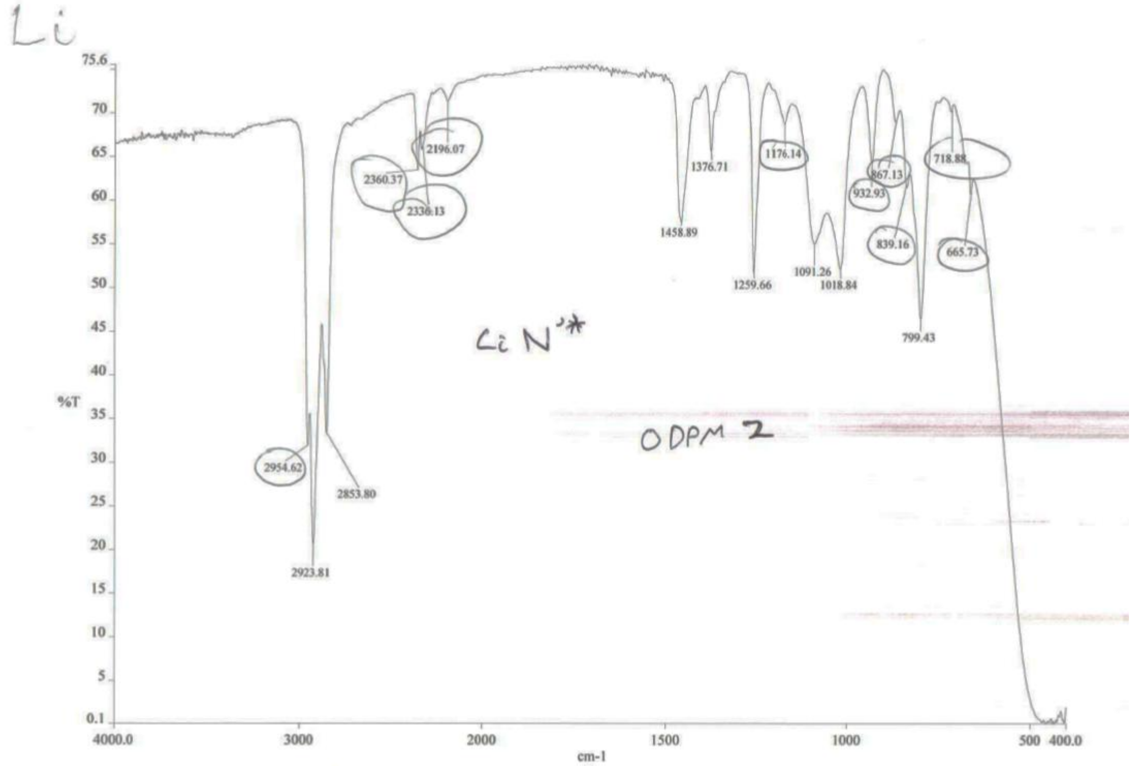

Figure S23. FTIR spectrum of $[Li\{\mu\text{-}N(Si^{t}BuMe_2)(SiMe_3)\}]_2$ (**2**) (Nujol mull).

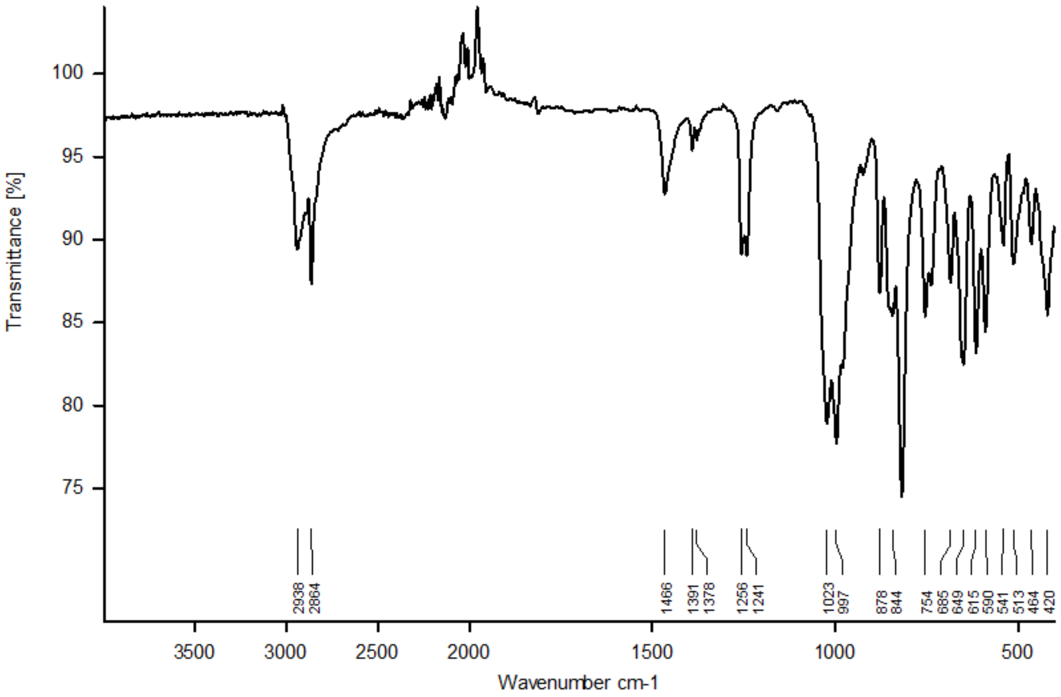

Figure S24. ATR-IR spectrum of $[Li\{\mu\text{-}N(Si^{i}Pr_3)(SiMe_3)\}]_2$ (**3**).

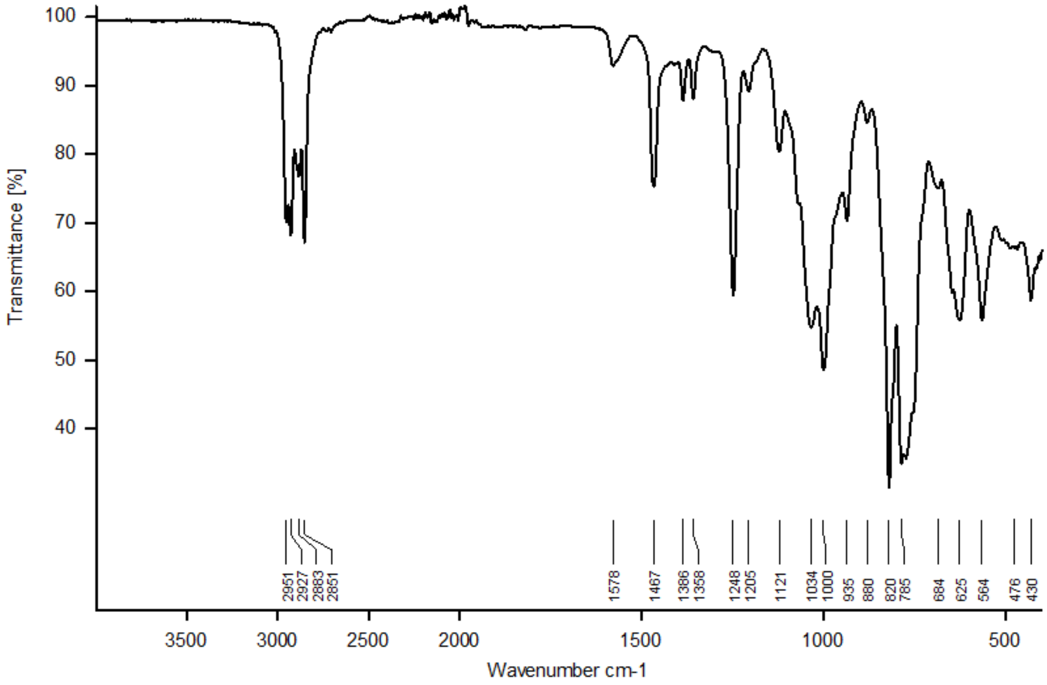

Figure S25. ATR-IR spectrum of [Li{N(Si^{t}BuMe$_2$)$_2$}{μ-N(Si^{t}BuMe$_2$)$_2$}Li(THF)] (**4**).

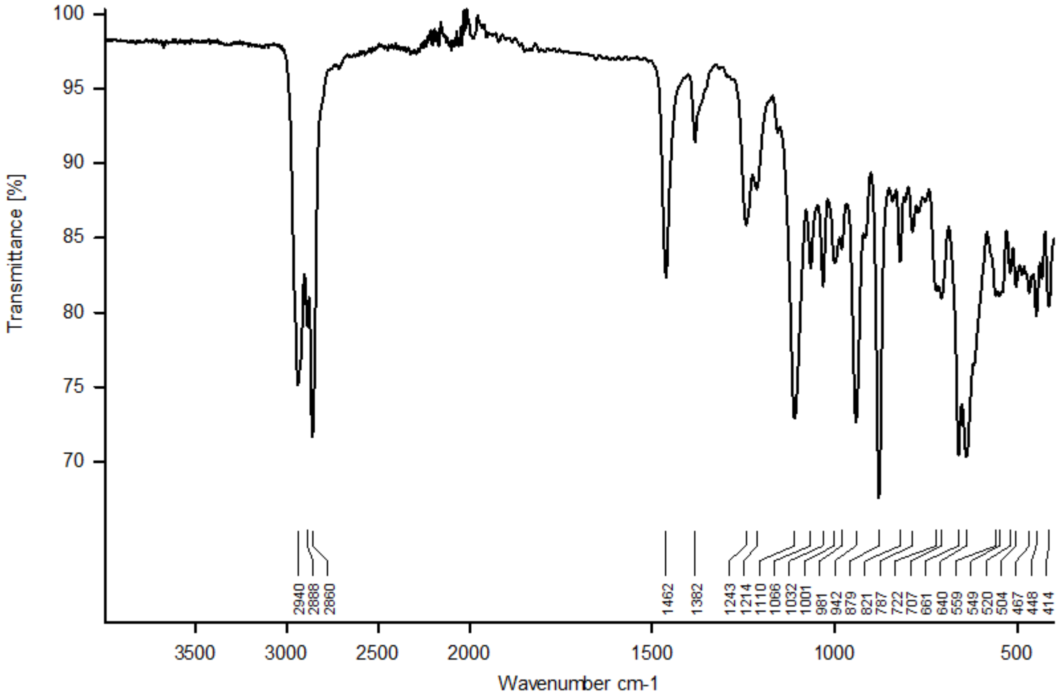

Figure S26. ATR-IR spectrum of [Li{N(Si^{i}Pr$_3$)$_2$}(THF)] (**7**).

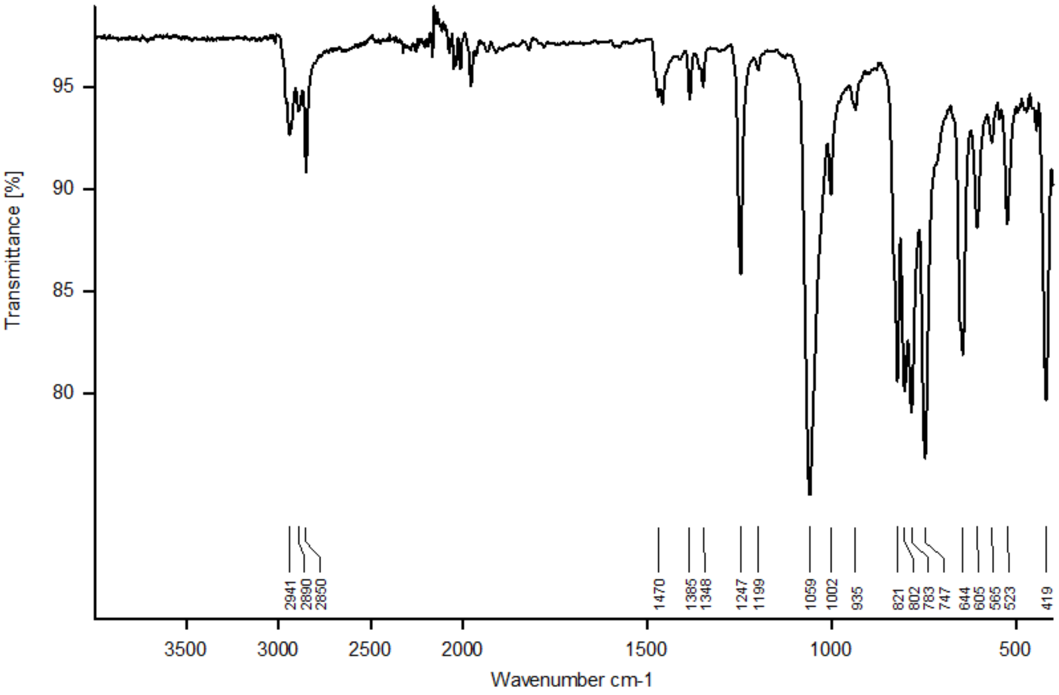

Figure S27. ATR-IR spectrum of [Na{N(Si^{t}BuMe$_2$)$_2$}]$_n$ (**9**).

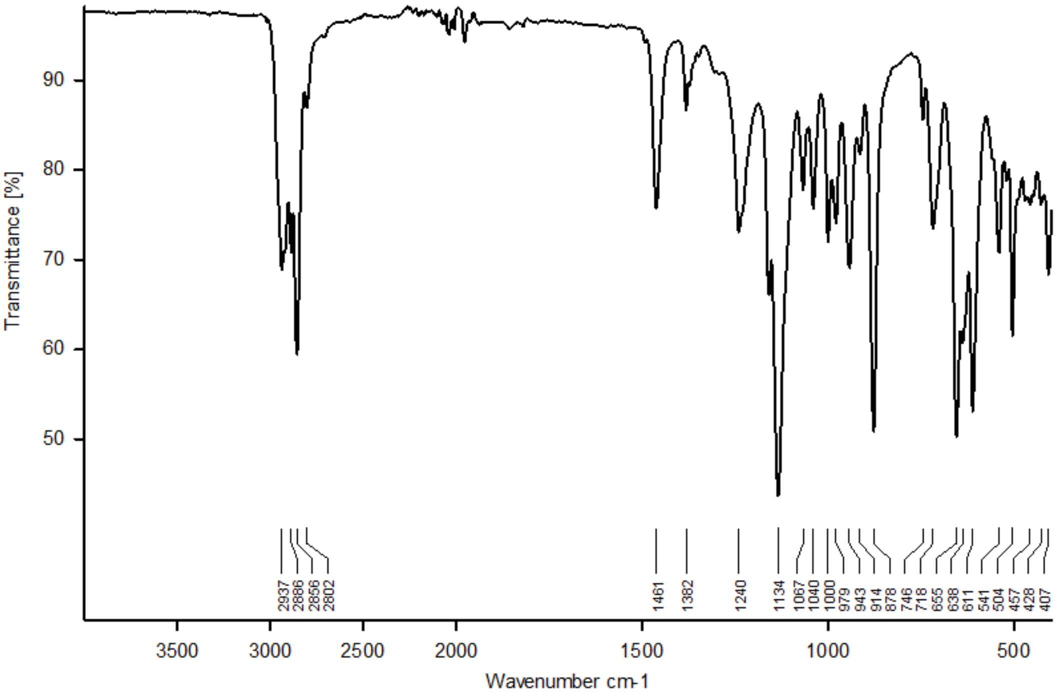

Figure S28. ATR-IR spectrum of [Na{N(Si^{i}Pr$_3$)$_2$}(C$_7$H$_8$)] (**10**).

3. X-ray Crystallography

Crystals of **1-10** were alternately examined on a Bruker Apex II diffractometer with a CCD area detector and a graphite-monochromated Cu Kα radiation (λ = 1.54178 Å) (**1** and **10**), a Rigaku Oxford Diffraction SuperNova CCD area detector diffractometer using mirror-monochromated Mo Kα radiation (λ = 0.71073 Å) (**3**, **5**-**6**, and **9-THF**), a Rigaku XtalLAB AFC11 diffractometer with a CCD area detector and graphite-monochromated Cu Kα radiation (λ = 1.54178 Å) (**7**) or an Oxford Diffraction Xcalibur diffractometer with a CCD area detector and a mirror-monochromated Mo Kα radiation (λ = 0.71073 Å) (**2**, **4** and **8**). Intensities were integrated from data recorded on 1° frames by ω and φ rotation in **10**; on 0.5° frames by ω and φ rotation in **1** and **7**; on 1.0° frames by ω rotation in **2**-**6**, and **8**; and on 0.9° frames by ω rotation in **9-THF**. Cell parameters were refined from the observed positions of all strong reflections in each data set. A Gaussian grid face-indexed absorption correction with beam profile modeling was applied in all instances through CrysAlisPro [1]. The structures were by intrinsic phasing in SHELXT [2] or direct methods in SHELXL [3] and the datasets were refined by full-matrix least-squares on all unique F2 values [**3**,3] with anisotropic displacement parameters for all non-hydrogen atoms, and with constrained riding hydrogen geometries; Uiso(H) was set at 1.2 (1.5 for methyl groups) times Ueq of the parent atom. The largest features in final difference syntheses were close to heavy atoms and were of no chemical significance. CrysAlisPro [1] was used for control and integration, and SHELX [3] was employed through OLEX2 [4] for structure solution and refinement. ORTEP-3 [5] and POV-Ray [6] were employed for molecular graphics. Supplementary crystallographic data for all complexes are compiled in Tables S1 and S2, whilst selected bond lengths and angles are compiled in Tables S3 and S4.

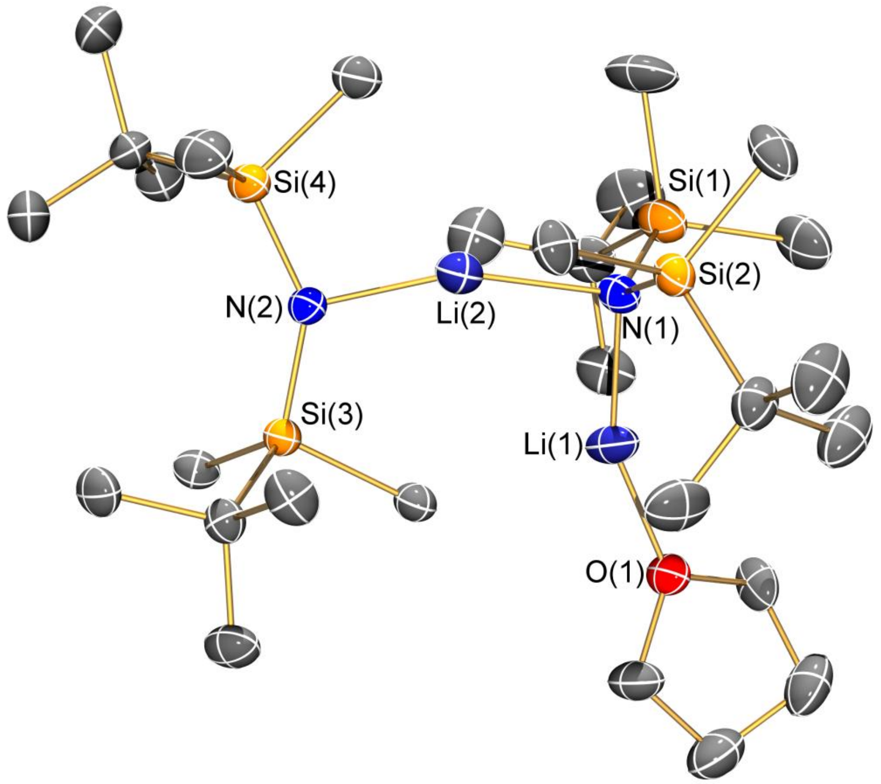

Figure S29. Molecular structure of [Li{N(Si^{t}BuMe$_2$)$_2$}{μ-N(Si^{t}BuMe$_2$)$_2$}Li(THF)] (**4**) with selective atom labelling. Displacement ellipsoids set at 30 % probability level and hydrogen atoms omitted for clarity.

Table S1. Selected crystallographic data for **1-5**.

	**1**	**2**	**3**	**4**	**5**
Formula	$C_{18}H_{43}NSi_2$	$C_{18}H_{48}Li_2N_2Si_4$	$C_{24}H_{60}Li_2N_2Si_4$	$C_{28}H_{68}Li_2N_2OSi_4$	$C_{32}H_{78}Li_4N_2Si_4$
Fw	329.71	418.82	502.98	575.08	631.08
Cryst size. mm	0.05 x 0.10 x 0.10	0.14 x 0.20 x 0.40	0.17 x 0.27 x 0.57	0.18 x 0.28 x 0.53	0.17 x 0.26 x 0.34
Crystal system	Monoclinic	Triclinic	Triclinic	Monoclinic	Triclinic
Space group	*P*21/*n*	*P*–1	*P*–1	*P*21/*c*	*P*–1
a, Å	8.81051(9)	10.2374(8)	12.4492(4)	16.2998(17)	9.1355(11)
b, Å	13.36759(16)	11.9719(8)	16.4469(6)	12.2405(7)	11.0906(18)
c, Å	18.58518(19)	12.6730(7)	17.6144(6)	20.6644(19)	12.644(2)
α, °	90	83.508(5)	67.932(3)	90	63.996(16)
β, °	99.1278(10)	76.177(6)	80.537(3)	112.441(12)	77.951(12)
γ, °	90	68.182(7)	89.926(3)	90	68.950(13)
V, Å^3	2161.16(4)	1399.69(18)	3289.6(2)	3810.7(7)	1072.5(3)
Z	4	2	4	4	1
ρ_{calcd}, g cm^3	1.013	0.994	1.016	1.002	0.977
μ, mm^{-1}	1.436	0.217	0.194	0.176	0.159
F(000)	744	464	1120	1280	352
No. of reflns (unique)	15671 (3938)	7336 (5102)	21307 (11975)	11902 (5777)	5957 (3865)
S^1	1.07	1.03	1.03	1.00	218
R_1(wR_2^{1}) (F^2>2σ(F^2))	0.0399 (0.1124)	0.0505 (0.1215)	0.0443 (0.1082)	0.0714 (0.1846)	0.0806 (0.2168)
R_{int}	0.024	0.024	0.021	0.076	0.033
Min/max diff map, Å^{-3}	–0.29, 0.44	–0.25, 0.40	–0.50, 1.03	–0.40, 0.69	–0.47, 0.51

[1] Conventional $R = \Sigma||F_o| - |F_c||/\Sigma|F_o|$; $R_w = [\Sigma w(F_o^2 - F_c^2)^2/\Sigma w(F_o^2)^2]^{1/2}$; $S = [\Sigma w(F_o^2 - F_c^2)^2/\text{no. data} - \text{no. params})]^{1/2}$ for all data.

Table S2. Selected crystallographic data for **6-10**.

	**6**	**7**	**8**	**9-THF**	**10**
Formula	$C_{19}H_{46}LiNO_2Si_2$	$C_{22}H_{50}LiNOSi_2$	$C_{26}H_{58}LiNO_2Si_2$	$C_{32}H_{76}N_2Na_2O_2Si_4$	$C_{25}H_{50}NNaSi_2$
Fw	383.69	407.75	479.85	679.28	443.83
Cryst size. mm	0.10 x 0.10 x 0.30	0.14 x 0.23 x 0.40	0.22 x 0.42 x 0.43	0.14 x 0.41 x 0.54	0.10 x 0.10 x 0.20
Crystal system	Monoclinic	Monoclinic	Triclinic	Triclinic	Monoclinic
Space group	*P*21/*c*	*P*21/*c*	*P*–1	*P*–1	*P*21/*c*
a, Å	17.6846(12)	16.30145(13)	10.5111(5)	9.9185(9)	18.2177(3)
b, Å	9.6929(6)	20.68971(16)	15.9508(6)	10.8375(7)	9.00185(10)
c, Å	30.5856(17)	16.01395(13)	18.7477(10)	11.0792(7)	17.2035(2)
α, °	90	90	85.086(4)	98.833(5)	90
β, °	104.603(7)	100.9135(8)	84.850(4)	103.526(6)	95.3841(12)
γ, °	90	90	83.646(4)	109.936(7)	90
V, $Å^3$	5073.5(6)	5303.38(7)	3102.2(3)	1052.45(15)	2808.81(6)
Z	8	8	4	1	4
ρ_{calcd}, g cm^3	1.005	1.021	1.027	1.072	1.050
μ, mm^{-1}	0.150	1.272	0.134	0.189	1.358
F(000)	1712	1824	1072	376	984
No. of reflns (unique)	18729 (10309)	39310 (9689)	19100 (1127)	7491 (4318)	14931 (5057)
S^1	1.02	1.05	1.02	1.03	1.04
$R_1(wR_2{}^1)$ ($F^2>2\sigma(F^2)$)	0.0571 (0.1419)	0.0395 (0.1080)	0.0576 (0.1545)	0.0594 (0.1561)	0.0363 (0.1030)
R_{int}	0.035	0.028	0.027	0.031	0.025
Min/max diff map, $Å^{-3}$	–0.56, 0.56	–0.53, 0.61	–0.37, 0.64	–0.41, 0.49	–0.30, 0.40

[1] Conventional $R = \Sigma||F_o| - |F_c||/\Sigma|F_o|$; $R_w = [\Sigma w(F_o^2 - F_c^2)^2/\Sigma w(F_o^2)^2]^{1/2}$; $S = [\Sigma w(F_o^2 - F_c^2)^2/\text{no. data} - \text{no. params})]^{1/2}$ for all data.

Table S3. Selected bond lengths (Å) and angles (°) for **1-5**.

	**1**	**2**	**3**	**4**	**5**
N(1)–Si(1)	1.7348(12)	1.701(2)	1.711(2)	1.712(4)	1.714(4)
N(1)–Si(2)	1.7370(12)	1.704(2)	1.707(2)	1.728(4)	1.713(5)
Li(1)–N(1)	-	2.015(5)	2.022(4)	1.974(10)	1.986(9)
Li(1)–N(2)	-	1.986(5)	2.032(4)	-	-
Li(2)–N(1)	-	1.982(5)	2.036(4)	2.026(8)	1.946(12)
Li(2)–N(2)	-	2.023(5)	2.030(4)	1.915(9)	-
N(2)–Si(3)	-	1.706(2)	1.708(2)	1.667(4)	-
N(2)–Si(4)	-	1.701(2)	1.705(2)	1.679(3)	-
Li(1)–O(1)	-	-	-	1.891(8)	-
Li(1)–C(13)	-	-	-	-	2.037(9)
Li ··C distances < 2.5 Å	-	2.378(6)	2.378(6)	-	-
	-	2.392(7)	2.392(7)	-	-
	-	2.393(7)	-	-	-
	-	2.427(6)	-	-	-
Si(1)–N(1)–Si(2)	145.43(8)	125.33(12)	127.91(11)	121.0(2)	131.0(3)
Si(3)–N(2)–Si(4)	-	125.29(13)	127.87(11)	144.2(2)	-
N(1)–Li(1)–N(2)	-	109.1(2)	110.1(2)	-	
N(1)–Li(2)–N(2)	-	109.0(2)	110.0(2)	160.2(4)	-
Li(1)–N(1)–Li(2)	-	71.0(2)	69.8(2)	97.6(3)	95.7(5)
Li(1)–N(2)–Li(2)	-	70.8(2)	69.5(2)	-	-
N(1)–Li(1)–O(1)	-	-	-	155.4(5)	-
N(1)–Li(1)–C(13)	-	-	-	-	171.9(5)
N(1A)–Li(2A)–C(13)	-	-	-	-	173.9(7)
Li(1)–C(13)–Li(2A)	-	-	-	-	91.7(4)

Table S4. Selected bond lengths (Å) and angles (°) for **6-10** (M = Li, **6-8**; Na, **9-10**).

	**6**	**7**	**8**	**9-THF**	**10**
N(1)–Si(1)	1.681(2)	1.6761(13)	1.686(2)	1.697(3)	1.6709(12)
N(1)–Si(2)	1.679(5)	1.6805(13)	1.681(2)	1.702(3)	1.6710(13)
M(1)–N(1)	1.894(13)	1.893(4)	1.953(5)	2.490(2)	2.2608(14)
M(1)–N(2)	-	-	-	-	-
M(2)–N(1)	-	-	-	-	-
M(2)–N(2)	-	-	-	-	-
M(1)–O(1)	1.984(5)	1.866(3)	1.987(5)	2.328(3)	-
M(1)–O(2)	1.958(3)	-	1.957(5)	-	-
Na(1) ··C(19)	-	-	-	-	2.912(2)
Na(1) ··C(20)	-	-	-	-	2.815(2)
Na(1) ··C(21)	-	-	-	-	2.801(2)
Na(1) ··C(22)	-	-	-	-	2.870(2)
Na(1) ··C(23)	-	-	-	-	2.935(2)
Na(1) ··C(24)	-	-	-	-	2.956(2)
Na(1) ··$Ph_{centroid}$	-	-	-	-	2.527(2)
Si(1)–N(1)–Si(2)	135.08(13)	142.07(8)	143.06(13)	124.18(14)	144.09(8)
N(1)–Na(1)–N(1A)	-	-	-	108.47(7)	-
Na(1)–N(1)–Na(1A)	-	-	-	71.53(7)	-
N(1)–M(1)–O(1)	143.4(3)	168.7(2)	132.5(3)	126.51(7)	-
N(1)–M(1)–O(2)	133.6(2)	-	130.7(3)	-	-
O(1)–M(1)–O(2)	82.8(2)	-	96.6(2)	-	-

4. References

1. *CrysAlis Pro*, Agilent Technologies: Yarnton, England, 2010.
2. Sheldrick, G.M. A short history of *SHELX*. *Acta Cryst., Sect. A.* **2008**, *64*, 112–122.
3. Sheldrick, G.M. Crystal structure refinement with *SHELXL*. *Acta Cryst., Sect. C.* **2015**, *71*, 3–8.
4. Dolomanov, O.V.; Bourhis, L.J.; Gildea, R.J.; Howard, J.A.K.; Puschmann, H. *OLEX2*: a complete structure solution, refinement and analysis program. *J. Appl. Cryst.* **2009**, *42*, 339–341.
5. Farrugia, L.J. *WinGX* and *Ortep* for Windows: an update. *J. Appl. Cryst.* **2012**, *45*, 849–854.
6. *POV-Ray*, Persistence of Vision Raytracer Pty. Ltd.: Williamstown, Australia, 2004.
